# Acknowledgment to the Reviewers of *Cancers* in 2022

**DOI:** 10.3390/cancers15030631

**Published:** 2023-01-19

**Authors:** Cancers Editorial Office

**Affiliations:** MDPI AG, St. Alban-Anlage 66, 4052 Basel, Switzerland

High-quality academic publishing is built on rigorous peer review. *Cancers* was able to uphold its high standards for published papers due to the outstanding efforts of our reviewers. Thanks to the efforts of our reviewers in 2022, the median time to first decision was 18 days and the median time to publication was 40 days. Regardless of whether the articles they examined were ultimately published, the editors would like to express their appreciation and thank the following reviewers for the time and dedication that they have shown *Cancers*:
A. Diana AndrushiaLeszek KrólickiA. Emre SayanLeticia Oliveira-FerrerA. R. M. Saifuddin EkramLetizia DeantonioA. Simon CarneyLetizia MezzasomaAaron M. UdagerLetizia PorcelliAaron MuthLeyre ZubiriAaron NilsenLi ChangAaron SarverLi HongAaron StevensLi JiangAaron Y. MochizukiLi LiAarti SharmaLi YangAasma ShaukatLi ZhangAbass AlaviLi ZhaoAbbey DiazLia VerhoefAbdelhakim BouyahyaLiane R. CampbellAbdelhalim EbaidLiang KangAbdelilah AboussekhraLiang LiuAbdollah AllahverdiLiang Ting LinAbdollahyan MaryamLiang WangAbdou Azaque ZouréLiang YanAbdul HannanLiang ZhangAbdul SirajLiang ZhongAbdulhameed Al-GhabkariLiang-Yo YangAbdulla Al-RashdanLianna MarksAbdullah Bin ShamsLianne M. HavemanAbdullah Saad Almalaise AlghamdiLianxin LiuAbeda JamadarLibo ZhangAbedalrhman AlkhateebLi-Ching ChangAbel JosephLiching ChenAbha GuptaLi-Chun HsiehAbhijeet PataskarLichun WeiAbhijit ChakrabortyLicun WuAbhijit KaleLidia GaffkeAbhik SahaLidia GattoAbhilash SamykuttyLidia StrigariAbhilasha SinhaLidija TodorovicAbhimanyu ThakurLido CaloriniAbhinav DubeyLigen YuAbhishek TyagiLi-Han HsuAbhishek VartakLihong HaoAbhishesh MehataLihong LiAbid H. BandayLili LiAbilash NairLili YangAbitha SukumaranLiliana ChemelloAbraham O. SamsonLiliana Łykowska-SzuberAbraham PouliakisLiliana MontellaAbraham TsitlakidisLiliana RogozeaAbu HazafaLily LaiAbu Hena Mostafa KamalLim Geok HoonAchilleas MitrakasLiming ChenAda FunaroLiming XieAdam A. FilipczykLimor HelpmanAdam BrentnallLin QiuAdam C. SmithLin WuAdam E. SnookLin YangAdam GolabLin YeAdam HaggLina BaranauskieneAdam J. KrysztofiakLina Bezdetnaya-BolotineAdam K. WalkerLina ZuccatostaAdam PłużańskiLinbo ZhaoAdam ReichLinchong SunAdam SperlingLinda Björkhem-BergmanAdam SzpechcińskiLinda HenricksAdarsh K. GuptaLinda SmitAdel El-NaggarLindsay B. AlcarazAdel SamsonLindsay KurokiAdela CañeteLindsay PuckettAdhemar Longatto-FilhoLindsey R. ConroyAdílson Jesus Aparecido De OliveiraLing GaoAdina Emilia CroitoruLing HeAditi BhattLing LiAditi JainLing YinAditi KulkarniLingeng LuAditi PrabhakarLingling MiaoAditya SarodeLingling ZhengAditya WaghLing-Wen DingAdrian Blanco-GomezLingzhi HongAdrian Elmi-TeranderLinli ZhouAdrian Kah Heng ChiowLinlin GuoAdrian P. WiegmansLinsler StefanAdrian Von WitzlebenLionel Fernel GamarraAdriana Corina HanganLionel LarueAdriana NeagosLior Z. BraunsteinAdriano AngelucciLip Wai LeeAdrienne L. WatsonLiqiang XiAe Jung HyunLisa BashoreAfsheen RazaLisa BauleoAfua Adjeiwaa MensahLisa CrawfordAfzal ShaikLisa CunninghamAgata GóźdźLisa I. WadiuraAgata Jabłońska-TrypućLisa L. LiuAgata Kabała-DzikLisa OomsAgata MarjańskaLisa PangAgata Poniewierska-BaranLisa Privette VinnedgeAglaia PappaLisa SevenichÁgnes EnyediLisa WebberÁgnes TantosLisa WiesmüllerAgnès V. TalletLisardo BoscaAgnese PoLise LecointreAgnieszka B. BialkowskaLisheng WangAgnieszka BiałekLishi XieAgnieszka Bojarska-JunakLisiane MeiraAgnieszka Dzikiewicz-KrawczykLiu LiuAgnieszka E. DacaLiubov BuchynskaAgnieszka GirstunLiudmila MatskovaAgnieszka Jagiełło-GruszfeldLiv Bolstad HysingAgnieszka KosowskaLiv ThommesenAgnieszka Lecka-AmbroziakLiverana LaurettiAgnieszka Mazur-BialyLivia ManzellaAgnieszka Owczarczyk-SaczonekLivia MarrazzoAgnieszka PiaseckaLivia RuffiniAgnieszka Piekiełko-WitkowskaLivnat Jerby-ArnonAgnieszka PotęgaLiwei LangAgnieszka ŚmieszekLiya XuAgnieszka Strzelecka-KiliszekLiye HeAgnieszka Tafelska-KaczmarekLiyong LinAgnieszka Wolnicka-GlubiszLi-Yuan BaiAgnieszka ZyromskaLjubica Glavas-ObrovacAgostino BucaloLloyd MackAguiari GianlucaLlucia AlosAgustín LahozLoic FeuvretAhmad JaliliLoic LemonnierAhmad SayasnehLois A. DaamenAhmed A. Abd El-MaksodLola Jade PalmieriAhmed A. El-GendyLone Winther LietzenAhmed El-FiqiLong Chiau MingAhmed ElkamhawyLong YuanAhmed ElnakibLong ZhengAhmed Hussieny ElbarbaryLongfei LiuAhmed I GhanemLongsheng LuAhmed IdbaihLoning FuAhmed Jibril AbdiLopez GianlucaAhmed M. Abu-DiefLoredana AmorosoAhmed MohsenLoredana BergandiAhmed MoustafaLoredana MarcuAhmed NoreldinLoredana Sabina Cornelia ManolescuAhmed OthmanLoredana UrsoAhmed S. MandourLoredana ZocchiAhmed Samir AlfaarLorelei Irina BrasoveanuAhmedova AnifeLoren D. FastAhmet Emre EskazanLorena DucaAiham QdaisatLorena TorroniAija LinēLorena UrbanelliAimilia GastouniotiLorenz BastianAino SiltariLorenza TrabalziniAirat BilyalovLorenzo AntonuzzoAirazat M. KazaryanLorenzo BianchiAisha FarhanaLorenzo CorsiAishwarya GurumurthyLorenzo D'AmbrosioAishwarya IyerLorenzo F. SempereAishwarya RavindranLorenzo FaggioniAitor BenedictoLorenzo FalchiAjay BhandariLorenzo G. LucianiAjay GuptaLorenzo LiviAjay KumarLorenzo MantiAjaybabu V. PobbatiLorenzo MonfardiniAjinkya KawaleLorenzo NeviAjit RoyLorenzo PredaAjmer Singh GrewalLorenzo SpaggiariAkhilendra Kumar MauryaLorenzo ZaffiriAki FurusawaLoreta StrumylaiteAki ManninenLori BanksAkihide YoshimiLouis BuscailAkihiko MiyanagaLouis J. VaickusAkihiro KuwahataLouis JustementAkiko TakahashiLouisa BolmAkiko TodakaLourdes A. FortepianiAkinobu OtaLourdes Cortes-DericksAkira KobayashiLourdes GimenoAkira MinouraLovorka StojicAkira SakamakiLu LengAkira SatoLu Ping TanAkira UmemuraLuana LuginiAkiyoshi KinoshitaLubna ChaudharyAkiyoshi TakamiLuc OllivierAkshara RaghavendraLuc WillemsAkshata NaikLuca BertolacciniAkshay PandeyLuca BurroniAlaa OthmanLuca CevolaniAla-Eddin Al MoustafaLuca CimaAlain Antoine MinaLuca Di GianfrancescoAlain MorelLuca FalzoneAlakananda BasuLuca FilippiAlan C. HalpernLuca GazziniAlan T. BlankLuca GiacomelliAlan WuLuca GiannellaAlana ThackrayLuca GianottiAlba CorellLuca Giovanni CampanaAlban GiraultLuca GrumolatoAlbert MoralesLuca IelasiAlbert Santamaria-MartínezLuca LazzariAlbert WölflerLuca MenichettiAlberta BergamoLuca MiloneAlberto AnelLuca MorandiAlberto BartoloméLuca MorelliAlberto BongiovanniLuca NespoliAlberto Di NapoliLuca NicosiaAlberto FelettiLuca OngaroAlberto Fernández-MedardeLuca PaciniAlberto FerrareseLuca PalazzoAlberto IngaLuca PasquiniAlberto IzzottiLuca PierelliAlberto LapiniLuca PossentiAlberto MaccariLuca SigalottiAlberto MittoneLuca TamagnoneAlberto PiccaLuca UrsoAlberto RagniLuca ValerioAlberto RighiLuca ViganoAlberto Rubén OsellaLucas D. DiasAlberto SaitaLucas DelmonicoAlberto SignoreLucas Ferrari De AndradeAlberto SinibaldiLucas MarquesAlbertus Theodorus HesselinkLucas Willian ThornbladeAlbino EccherLucia ContiAlciviadis-Constantinos CefalasLucía Fernández CasanovaAldesia ProvenzanoLucia KučerováAldo A. Rodrıguez-MenchacaLucia LisiAldo PaganoLucia MangoneAldo PezzutoLucia MonacoAldo RoccaLucia MorbidelliAldo VenutiLucia MusacchioAldona KasprzakLucia R. LanguinoAldona KowalskaLucia SacchettiAlec J. KacewLucia StančiakováAlejandra Gomez CadenaLucia TrevisiAlejandra PeraLucía Trilla-FuertesAlejandra Sanjuan PlaLuciana CorrêaAlejandro Herreros-PomaresLuciana Pereira RangelAlejandro SaettoneLuciana VintiAlejandro San Juan FerrerLuciano AndreozziAlejandro Schcolnik-CabreraLuciano FurlanettiAleksander BilewiczLucie SanceyAleksander ŚlusarczykLucija GosakAleksandr BugayLucillia BezuAleksandra A StefaniakLucio MangoAleksandra AsaturovaLucy Anne ParkerAleksandra Gilis-JanuszewskaLucy NorrisAleksandra GlogowskaLucyna KepkaAleksandra RodackaLudmiła Grzybowska-SzatkowskaAleksandra SwierczLudovic DumontAleksei A. StepanenkoLudovic FournelAleksei TikhonovLudovico CarboneAlena FurdovaLudwig J. DuboisAlena Ribeiro Alves Peixoto MedradoLu-Han LaiAlena SkálováLui NgAleš RozmanLuigi AlbanoAlessandra AmmazzalorsoLuigi BonavinaAlessandra ArcelliLuigi CerboneAlessandra BorgheresiLuigi CoppolaAlessandra CapuanoLuigi CormioAlessandra CinqueLuigi FattoreAlessandra PicardiLuigi FormisanoAlessandra RaimondiLuigi LoscoAlessandra RosatiLuigi MeccarielloAlessandra SinopoliLuigi NavasAlessandro AntonelliLuigi NoceraAlessandro BaisiLuigi PirtoliAlessandro BonardiLuigi RacioppiAlessandro CamaLuigi SansoneAlessandro CaputoLuigi SchipsAlessandro CarrerLuigi SpiazziAlessandro ClivioLuigia Stefania StucciAlessandro ComandoneLuis A. MartinezAlessandro Del GobboLuis A. Pérez-RomasantaAlessandro FanzaniLuis BujandaAlessandro FratiLuis ColomoAlessandro GambellaLuis De la Cruz-MerinoAlessandro GhelardiLuis Del Pozo-YaunerAlessandro GonfiottiLuis E. IbarraAlessandro GranitoLuis Enrique Juárez-VillegasAlessandro LiquoriLuis Felipe Jave SuarezAlessandro LoglioLuis Fernando Macedo Di CristofaroAlessandro MalobertiLuís Gafeira GoncalvesAlessandro Marco MinisiniLuis González-BayónAlessandro MassaroLuis LarreaAlessandro MaurielloLuis Leon MateosAlessandro MazzottaLuís LopesAlessandro MoiraghiLuis Mariano EstebanAlessandro MorabitoLuís Pacheco-FigueiredoAlessandro MussaLuis RaposoAlessandro OttaianoLuis RodrigoAlessandro PesceLuisa FiandraAlessandro PileriLuisa GuidiAlessandro PoggiLuisa PetroneAlessandro PratesiLuisa Vera MuscatelloAlessandro ProvenzaniLuka FlegarAlessandro PulsoniLukas Carl HeukampAlessandro RizzoLukas HegerAlessandro RussoLukas John HefermehlAlessandro SammarcoLukas KäsmannAlessandro SgambatoLukas KennerAlessandro StefanoLukas LacinaAlessandro TafuriLukas PeintnerAlessandro TelLukas StalpersAlessandro VaiŁukasz B. LewandowskiAlessandro WeiszŁukasz BołkunAlessia CarboniŁukasz KuncmanAlessia CozzolinoŁukasz LewandowskiAlessia LambertoniŁukasz MasiorAlessia PellerinoŁukasz NawackiAlessia RemiganteŁukasz ZapałaAlessio AlbaneseLuke PaterAlessio ArdizzoneLulu CaiAlessio BaccaraniLun Kelvin TsouAlessio ContiLunda ShenAlessio CortelliniLuo ZhenwuAlessio D'AlessioLuo ZhiguoAlessio Danilo ComisLusine H. DemirkhanyanAlessio GiubellinoLu-Ting KuoAlessio MorgantiLutz KretschmerAlessio PaladiniLutz Martin WeiseAlex AlfieriLuyao ZhouAlexa BodmanLuz Vazquez-MorenoAlexandar TzankovLydia GabaAlexander BrobeilLydia MederAlexander BunevLydia ScarfòAlexander CarpinteiroLydia VisserAlexander E. BerezinLynn McKeownAlexander GasparskiLynne BingleAlexander KabakovLynne HowellsAlexander KlegerLynnette M. JonesAlexander KleinM. Aiman MohtarAlexander KorthausM. Anwar IqbalAlexander KutikovM. Canan ArkanAlexander L. LazaridesM. CellinaAlexander LeichtleM. HassaballahAlexander MuacevicM. R. MozafariAlexander MuirM. S. MuthuAlexander OtahalMa. del Rocío Baños-LaraAlexander PemovMaaike C. G. BleekerAlexander PhilippMaarit AhtiainenAlexander QuaasMaarten C. BoslandAlexander S. MalogolovkinMaarten F. BijlsmaAlexander ShtilMaarten H. LequinAlexander StahlMaarten HoogenkampAlexander TamalunasMaarten L. DonswijkAlexander Thomas John LeeMaciej GagatAlexander ToliosMaciej Iek SkrzypczakAlexander WinterMaciej JaneczekAlexander WuMaciej MilanowskiAlexander WurzerMaciej SkrzypczakAlexander YakobsonMaciej TarnowskiAlexandra CaziucMaciej W. SochaAlexandra Cristina MunteanuMack K. RoachAlexandra GangiMadaswamy S. MuthuAlexandra KalogerakiMadhu OusephAlexandra M. MowdayMadhubanti MullickAlexandra M. PsihogiosMadhuja SamaddarAlexandra WinkelerMadhukar ThakurAlexandre BozecMadhup RastogiAlexandre BuffetMadhuri WadehraAlexandre EscargueilMadlen JentzschAlexandre Gaspar-MaiaMaedeh MohebnasabAlexandre MaréchalMaël HeibligAlexandre PeltierMaellaro EmiliaAlexandre T. J. MariaMafalda PintoAlexandros DaponteMafalda VideiraAlexandros G. SykarasMagali TermeAlexandros GeorgakilasMagda AbdellattifAlexandros LaiosMagda ZanelliAlexandros PapachristodoulouMagdalena BieniaszAlexandru A. SaboMagdalena Dutsch-WicherekAlexey A. LarionovMagdalena GörtzAlexey ChubarovMagdalena Mackiewicz-MilewskaAlexey ChurovMagdalena Markowicz-PiaseckaAlexey G. MittenbergMagdalena RatajskaAlexey KovalMagdalena RauschAlexey KuzmichMagdalena SkórzewskaAlexey V. PetukhovMagdalena StaniszewskaAlexey V. StepanovMagdalena SzopaAlexi KissMagdalini MigkouAlexis TalbotMagdolna SzantoAlfeu Zanotto-FilhoMaguy Del RioAlfizah HanafiahMahadev RaoAlfons LawenMahdieh MehrpouriAlfonso Blázquez-CastroMahendiran DharmasivamAlfonso W. AvolioMahendran SekarAlfredo BerrutiMahesh GokaraAlfredo BudillonMahesh KasthuriAlfredo ContiMahitosh MandalAlfredo Cruz-GregorioMahmood Al-khassawenehAlfredo MartínezMahmoud A. SenousyAlfredo NicosiaMahmoud AhmedAlfredo TartaroneMahmud HasanAlgimantas TamelisMai-Britt W. ØrntoftAlgirdas Edvardas TamošiūnasMaik LuuAli BahadurMailin GanAli Ekrem YesilkanalMailyn Pérez LivaAli JazirehiMaitane AsensioAli KhammanivongMaja CupicAli Muhamed AliMaja H. OktayAli S. HaiderMaja Herak BosnarAli ZarrabiMaja KrajinovicAlia D. GhoneumMaja NiksicAlice BoilèveMaja SabolAlice BongMaja T. TomicicAlice BonomiMajdy IdreesAlice CortesiMajid KhatibAlice MeroniMajid VahedAlice RossiMakbule TambasAlicia Rodríguez-BarberoMakito MiyakeAlicia Romero-LorcaMakoto ChumaAlicia RoqueMakoto EndoAlida F. W. Van Der SteegMakoto SakaiAline P. BeckerMalene Roland Vils PedersenAline Rangel-PozzoMalgorzata BajorAlireza MansouriMałgorzata BiałekAlireza ZarebidokiMałgorzata ChlabiczAlison BowersMałgorzata CzogałaAlison TaylorMalgorzata Czystowska-KuzmiczAlizadeh HussainMałgorzata KlimekAlkmini T. AnastasiadiMałgorzata Kotula-BalakAlla B. BucharskayaMalgorzata KrawczykAllison M. MartinMalgorzata KucinskaAllison N. SchorzmanMałgorzata KujawskaAllyson CochranMalgorzata Maria JerzakAlmudena PorrasMałgorzata Natalia MojsakAlok K. DwivediMałgorzata PasekAlok KhandelwalMalgorzata PieniazekAlok MishraMałgorzata Polz-DacewiczAlonso Martinez-CanabalMałgorzata PrzybyłoAlper TokerMalgorzata SkibinskaAltaf MohammedMałgorzata SochaÁlvaro GonzálezMalgorzata Sokolowska-WojdyloAlvaro Otero RodríguezMałgorzata Trofimiuk-MüldnerAlvin A. HolderMałgorzata WierzbickaAlvin ChandraMalik HamaidiaAlvise SernicolaMalin ÖstenssonAmadeus DobrescuMalin WickströmAman SharmaMalini SurapaneniAmancio CarneroMalka Cohen-ArmonAmanda C. SharkoMamatha GarigeAmanda GuthMame Daro FayeAmanda J. CraigMamoru HaradaAmanda M. ClarkMamoru KusakaAmanda WasylishenMamoru TakenakaAmaniel KefleyesusMamoru TanakaAmantini ConsueloMamta GuptaAmaresh PandaManabu KatoAmarish YadavManabu NatsumedaAmblessed OnumaManabu YamazakiAmbra BuschiazzoManal S. FawzyAmbra GrollaMandy BeyerAmeer Fawad ZahoorManel Juan OteroAmeet A. ChimoteManendra Babu LankadasariAmelia Maria GămanManfred GesslerAmelia PalermoManfred SchmidtAmelia RizzoMangalathu S. RajeevanAmelia SmitManinder K. AhluwaliaAmelia V. García-LuengoManish AdhikariAmélie RebillardManish CharanAmélie TrinquandManish DhawanAmer AhmedManish R. PatelAmina BolatkanManishkumar PatelAmir AshoorzadehManjit KaurAmir SabetManoj AmrutkarAmir SohrabiManoj GargAmira AbdellatefManoj KumarAmirata Saei DibavarMansoor HussainAmirhossein BahreyniManu JainAmirmasoud AhmadiManu PrasadAmit D. GujarManuel BandeAmit KhotManuel Durán PovedaAmit KumarManuel MoralesAmit SethiManuel PeruchoAmit TripathiManuel Rodríguez-PerálvarezAmitava MukherjeeManuel StruckAmmad Ahmad FarooqiManuela A. HoffmannAmmar BaderManuela Adelinde AschauerAmnon BrzezinskiManuela CipollettiAmol SuryawanshiManuela CurcioAmos KirilovskyManuela Klingler-HoffmannAmos SakweManuela MatesanAmr AminManuela PiazziAmr MahranManuela RabaglioAmr MohamedMao Qiang WangAmrita CheemaMaoquan ChuAmutha RamadasMara CarsoteAmy BodyMara MazzoniAmy McCart ReedMara P. SteinkampAmye J. TevaarwerkMara Pavel-DinuAn CoosemansMarc BeltràAn LiuMarc Benjamin HahnAn XuMarc BernaAna Alfonso PiérolaMarc BilodeauAna Aurora Díaz-GavelaMarc De La RocheAna Bela Sarmento-RibeiroMarc Jeffrey GollubAna Belén HerreroMarc OlivaAna Belen Perez-OlivaMarc OsterlandAna C. Martins CavacoMarc PocardAna C. XavierMarc Schmidt-SupprianAna CaruntuMarc StemmlerAna Cristina QueirósMarcel HörningAna D. MartinsMarcel NijlandAna DekanicMarcel SchmidtAna E. Rodríguez-VicenteMarcela LizanoAna Florencia Vega-BenedettiMarcela Manrique MorenoAna Godoy OrtizMarcella BarbarinoAna Guerra-LibreroMarcella MassiminiAna Isabel Alvarez-MercadoMarcello Di MartinoAna Lucía De PaulMarcello ManfrediAna Luísa De Sousa-CoelhoMarcello PersicoAna Luísa SilvaMarcello TiseoAna M. CardenasMarcelo A. CarvalhoAna M. Carmona-RibeiroMárcia Marília Gomes Dantas LopesAna Maria Da Costa FerreiraMarcin A JedrykaAna OsorioMarcin BalcerzykAna Patrícia CardosoMarcin IwanickiAna Podolski-RenićMarcin JaneckiAna PretoMarcin JozwikAna RamosMarcin NicośAna Ruiz-SáenzMarcin OstrowskiAna Sofia Leitão CarvalhoMarcin SajekAna TadijanMarcin SurmiakAna Teresa G. FernandesMarcin ZemanAnabel SorollaMarcin ZiętekAnamaria BrozovicMarco AgostiniAnamarija ŠestakMarco Antonio Meraz-RíosAnand RamanathanMarco BaralleAnand RotteMarco BiffoniAnand SharmaMarco BocchettiAnand SinghMarco BruschiAnao ZhangMarco CalvarusoAnas ShamsiMarco CerranoAnastasia A. PonomaryovaMarco CroccoAnastasia De LucaMarco De MartinoAnastasia KarlsonMarco De SioAnastasia MpakaliMarco E. M. PelusoAnastasia PapachristodoulouMarco FalascaAnastasios GkountakosMarco G. AlvesAnastasios KoulaouzidisMarco GaviraghiAnastasios MarkopoulosMarco GuzzoAnastassios PhilippouMarco HerlingAnat BiegonMarco InamaAnca Chelariu-RaicuMarco KrengliAnca ColitaMarco LocatelliAnca FranziniMarco Lorenzo BonùAncel JulienMarco LucchiAn-Chi TienMarco ManfrediniAncuta JurjMarco MarcascianoAncy JosephMarco MascittiAnders Christian LarsenMarco MontorsiAnders MellemgaardMarco MorselliAnders ÖrbomMarco MoschettaAnders Rosendal KorshoejMarco NisiAnders StrömMarco OrecchioniAnderson RyanMarco PaggiAndi CaniMarco PalancaAndra PiciuMarco PicardiAndrás DérMarco PonzettiAndras PiffkoMarco SalvatoreAndrás SzászMarco ScatizziAndraz DovnikMarco SchiaritiAndraẑ DovnikMarco SegattoAndre GoyMarco Stefano DemarchiAndré KhalilMarco TomasettiAndré Luís MoreìraMarco TonelloAndre MenkeMarco TrerotolaAndré VisMarco V. CorniolaAndrea AlexandreMarco VivarelliAndrea AntonuzzoMarcos Roberto de OliveiraAndrea BacigalupoMarcus BahraAndrea BelliMarcus CzabankaAndrea BiccariMarcus GoncalvesAndrea BilgerMarcus HollenbachAndrea BolognesiMarcus HorstmannAndrea BordoniMardelle AtkinsAndrea BottiMarek KowalczykAndrea CaraiMarek MinarikAndrea CarugnoMarek MuriasAndrea CassoniMarek RusinAndrea CiavattiniMargalida Torrens-MasAndrea de VitoMargaret Eleanor CruickshankAndrea DekanicMargaret FitchAndrea Emanuele GueriniMargaret OttavianoAndrea FrillingMargaret T. KasnerAndrea FurkaMargarete OdenthalAndrea GallaminiMargarida Julia-SapeAndrea GalliMargarita MaurerAndrea GalosiMargarita RomanenkoAndrea GianniniMargarita ZaytsevaAndrea GiniMargherita CortiniAndréa Gutierrez MariaMargherita EufemiAndrea Hawkins-DaarudMargherita GhisiAndrea JaníkováMargherita SerraAndrea L. KleinMargherita SistoAndrea LaurenziMarguerite VignonAndrea LauterioMaria A. LoizidouAndrea M. PotocnyMaría A. Pérez-HerreroAndrea ManniMaria AdamakiAndrea MartiniMaria Alba SorollaAndrea MessoriMaria Alexandra PapadimitriouAndrea MinerviniMaria AlievaAndrea MorrioneMaria Al-MatarnehAndrea MulliriMaria AmbrosioAndrea NicoliniMaria AnastasiouAndrea PapaitMaria Angela Zaccarelli-MarinoAndrea PelosoMaria Antonia FrassanitoAndrea PiccinMaria Antonietta GambacortaAndrea RoccaMaria Antonietta MazzeiAndrea RomaniMaria Antonietta RagusaAndrea SaladinoMaria Aparecida NagaiAndrea SambriMaria Assunta ZoccoAndrea SistiMaria BabakAndrea ŠoltýsováMaria BartosovaAndrea SpecialeMaria BugalhoAndrea SpiniMaria BuoncervelloAndrea VolpeMaria C. KatapodiAndrea WeissMaria CaffoAndrea ZanelloMaria Candida Barisson Vilares FragosoAndreas F. MavrogenisMaria Carmela PiccirilloAndreas Ioannis PapadakisMaría Carmen MarquésAndreas JosefssonMaria Carolina MendesAndreas KarakatsanisMaria Caterina TurcoAndreas KriegMaria Cecilia Mendonca-TorresAndreas LambertzMaria Chiara BruneseAndreas LundqvistMaria Christina CoxAndreas MahnkenMaria Cristina CuriaAndreas PolydorouMaria Cristina DechecchiAndreas RoidlMaria Cristina SiniAndreas RösslerMaria Czyzyk-KrzeskaAndreas VitsosMaria De FalcoAndreas WeberMaria de Fátima Monginho BaltazarAndreas WeinhäuselMaria De La Luz Garcia-HernandezAndreea BorleaMaria DiabAndrei ColițăMaria DietrichAndrei FodorMaria Do Céu CostaAndrei HavasiMaría Dolores Chiara RomeroAndrei I. KhlebnikovMaría Dolores Llobat BordesAndrei LeitãoMaria E. MycielskaAndreia C. De MeloMaria E. R. Garcia-RenduelesAndréia De Melo PorcariMaria Elena MelicaAndrés Bueno-CrespoMaría Elena Padín-IruegasAndrés E. QuesadaMaria Felice BrizziAndrés García MonteroMaria FioreAndrés TittarelliMaria FortofoiuAndrew BatemanMaria Francesca BaiettiAndrew ByrnesMaria FrantziAndrew C. BirkelandMaria Fuglsang JensenAndrew ChanMaria Gabriela RasoAndrew ChantryMaria GhitaAndrew D. FrugéMaria Giovanna ScioliAndrew D. RedfernMaria Giulia BattelliAndrew DalbyMaria Giulia CangiAndrew DonsonMaria Gomes Da SilvaAndrew E. BlumMaria Grazia BorrelloAndrew G. ShumanMaria Grazia BottoneAndrew J. MurphyMaria Grazia CattaneoAndrew KatsifisMaria Grazia CerritoAndrew L. HongMaria Grazia TozziAndrew LissMaria GreabuAndrew M. FribleyMaria GuarinoAndrew M. FryMaria HawkinsAndrew M. PickeringMaria Hermínia DiasAndrew M. ScottMaría Isabel Del Olmo GarcíaAndrew M. WatersMaria Isabel Martinez MaciasAndrew N. HoldingMaría Isabel RodríguezAndrew NisbetMaría Jesús Alvarez-CuberoAndrew PageMaría Jesús Núñez-IglesiasAndrew QuestMaria João RamalhoAndrew R. MarleyMaría José de Jesús ValleAndrew RenehanMaría José García-PolaAndrew RoblesMaría José Molina-GarridoAndrew SulaimanMaría José Pérez LacastaAndrew W. MaksymiukMaria KamenetskyAndrew WotherspoonMaria Karoline AndersenAndrey ElchaninovMaria Laura AllendeAndrey FinegershMaría Laura GutiérrezAndrey SudarikovMaria LavdanitiAndrey V. KustovMaria LehmannAndroulakis IoannisMaria Lina TorneselloAndrzej DeptałaMaria Lorena AbateAndrzej KomorowskiMaria Lucia Hirata KatayamaAndrzej KoniecznyMaria Luisa BrandiAndrzej M. KurylcioMaria Luisa GarreAndrzej NowickiMaria Luiza S. MelloAndrzej SemczukMaría Luz MoralesAndrzej SkorekMaria Maddalena MarrapodiAndrzej SkrętMaria MonsalveAndrzej SlominskiMaria MorrisseyAndrzej StepulakMaria MudryjAndy Wei-Ge ChenMaria OrdonezAneta Cymbaluk-PłoskaMaria OtthAneta Szudy-SzczyrekMaria Paola ParonettoAnette Gjörloff WingrenMaria PapadoliopoulouAngel AlvarezMaria PapaioannouÁngel Hernández-HernándezMaria PapatsirouÁngel Luis García-OtínMaria Patrizia StoppelliÁngel MonteroMaría Pérez-CarriónÁngela Ayén-RodriguezMaria Pia ProttiAngela BentivegnaMaria PicchioAngela BriegerMaría Ramos-MonserratAngela Dalia RicciMaria RodriguezAngela GalloMaria Rosa CirioloAngela Georgia CaticMaria Rosa MazzoniAngela Johana Espejo-MojicaMaria Rosaria De MiglioAngela MastronuzziMaria Rosaria GaldieroÁngela RojasMaria Rosaria RuggieroAngela RussoMaría Ruiz LinaAngela SolanoMaria RussoAngela SpanuMaria SamaraAngélica FigueroaMaria Saveria Gilardini MontaniAngelika B. RiemerMaria Serafina RistaldiAngelika MuchowiczMaria SmolleAngelika MühlebnerMaría Stefania InfanteAngelique StalmachMaria StrazzulloAngelo CastelloMaria SundvallAngelo Di IorioMaria Teresa EspositoAngelo NaselliMaria Teresa InzitariAngelo VaccaMaria Teresa VietriAngelos KoutrasMaria TrovatoAngelos PapaspyropoulosMaria TsoliAnikó BozsikMaría Val Toledo LoboAnil K. GiriMaria VelegrakiAnil K. YadavMaría Villa MoralesAnil Kiran ChokkallaMaría Virginia CroceAnil KumarMaría Virumbrales-MuñozAnil SinghMaria ZellnerAnilchandra AttaluriMariachiara ZuccariniAnilkumar GopalakrishnapillaiMariaconsiglia FerrieroAnindit MukherjeeMarialaura PetroniAnindya Sundar RayMarialuisa LugaresiAnirban ChakrabortyMarialuisa PiccoloAnirban KarMariamena ArbitrioAnirban RoychowdhuryMarian GradeAnirudh GaurMariana FragosoAnish K. RayMariana MatiasAnish MukherjeeMariangela De RobertisAnita BakraniaMariangela GarofaloAnita Chudecka-GłazMariangela ManciniAnita Kloss-BrandstätterMariangela MassaccesiAnita Somborac-BačuraMariángeles DíazAnja Borgmann-StaudtMarianna Alunni-FabbroniAnja HarderMarianna AprileAnja KarlstaedtMarianna MartinelliAnja Kathrin WegeMarianna MasperoAnja Katrin BielinskyMarianna SillettaAnja M. BoosMarianna TzanoudakiAnja SmitsMarianna YakubovskayaAnja WesselyMarianne EwertzAnjali CremerMarianne HjermstadAnjali GeethadeviMarianne M. LeeAnjali KusumbeMarianne TinguelyAnjan Kumar PradhanMariano BizzarriAnke van den BergMariano Francesco CaratozzoloAnkie Tan CheungMariano García-ArranzAnkit GangradeMariano Martinez-VázquezAnkit ManglaMariarita SantiAnkit SrivastavaMarie C. BénéAnkur NaqibMarie DuhamelAnkur SharmaMarie E. BecknerAnn HopkinsMarie Louisa AntoniAnna Adamiok-OstrowskaMarie LudvikovaAnna AureliMarie Øbro FosbølAnna BajekMarie Pierre SunyachAnna Bednarska-CzerwińskaMarie SébertAnna BrzeckaMarie-Catherine VozeninAnna CarranoMarie-Claude JaurandAnna CieślińskaMarie-Hélène David-CordonnierAnna Cleta CroceMariella TeránAnna DarabiMarielle YoheAnna DupréeMarie-Louise KampmannAnna E. LokshinMarie-Thérèse ForsterAnna EiringMariia D. IvanovaAnna ErmundMarija GamulinAnna Esteve-CodinaMarija Grdić RajkovićAnna FakhardoMarija MilovanovicAnna G. SoraceMarija TakicAnna Giulia NappiMarijana PetkovićAnna GrendaMarike W. Van GisbergenAnna Grzywa-CelińskaMariko Okada-HatakeyamaAnna HymosMariko OkamotoAnna J. MilewskaMarilène FilbetAnna KakehashiMarília Arruda Cardoso SmithAnna KałużaMarilia BarrecaAnna Karin ElfMarilyn A. ArosemenaAnna KleczkaMarilyn HuangAnna KozłowskaMarilyne PoireeAnna L. GharibyanMarina AunapuuAnna L. SchwartzMarina BacciAnna LaurenzanaMarina Consuelo VitaleAnna Makuch-KockaMarina De RosaAnna Małgorzata CzarneckaMarina E. CazzanigaAnna Maria D'UrsiMarina K. IbragimovaAnna Maria TestiMarina MartelloAnna MarkiewiczMarina MencingerAnna MarkowskaMarina MuzzaAnna Martina BattagliaMarinela BostanAnna Mucha-MałeckaMario A. BernalAnna N. YaroslavskyMario AmmiratiAnna OdoneMario BilićAnna Paola CarrecaMario ChiarielloAnna PiekarskaMario Del RossoAnna RaciborskaMario GorenjakAnna RiesterMario GruppoAnna Rita Franco MigliaccioMario LevisAnna Rita MigliaccioMario LuppiAnna RoschkeMario MarchettiAnna RossettoMario MondelliAnna SantarsieroMario Pérez-SayánsAnna SebestyènMario PrestiAnna SobiepanekMario PretiAnna SokalskaMario TiribelliAnna StarzyńskaMario Wolfgang KramerAnna SuredaMario ZorattiAnna VähärautioMărioara ȘimonAnna ValentinoMariola BorowskaAnna VignatiMariola NapiórkowskaAnna WawruszakMarion R. CurtisAnna WuMarios E. FroudarakisAnnabel S. BerthonMarios SpanakisAnnalicia VaughanMarisa MenaAnnalisa ArcariMarisa R. BuchakjianAnnalisa AstolfiMarisa ShiinaAnnalisa Di CelloMaristella MaggiAnnalisa MasiMariusz FleszarAnnalisa QuattrocchiMariusz HartmanAnna-Maria BarciszewskaMariusz KlenckiAnnamaria BrioliMariya VorobyevaAnnamaria ColaoMariza VorsterAnnarita NappiMarjan BoermaAnnarosa ArcangeliMarjan MohammadiAnnat RaiterMark A. GlennAnne E. CressMark A. KleinAnne Harduin-LepersMark A. LaBargeAnne HerrmannMark ApplebaumAnne McLeer-FlorinMark BarokAnne PradinesMark BuzzaAnne Régnier-VigourouxMark C. GurkAnne RodallecMark E. SobelAnne Vibeke LænkholmMark G. H. ScottAnne W. BeavenMark G. WaughAnne W. HamburgerMark GorrellAnne-Josée GuimondMark HannibalAnnekathryn GoodmanMark I. van Berge HenegouwenAnnekatrin CoordesMark J. RoefAnnelieke DamenMark K. FarrugiaAnnelies M. C. Mavinkurve-GroothuisMark Ka Heng ChanAnne-Marie Cleton-JansenMark LabrecqueAnne-Sophie MehdornMark ParascandolaAnne-Sophie WoznyMark S. CondonAnnette Fisseler-EckhoffMark Ter LaanAnnette MollMark TitusAnnette PfahlbergMark V. SchaverienAnnette S. KimMark W. DonoghoeAnnette StaeblerMark W. KonijnenbergAnneza YiallourouMarko Č. BarjaktarovićAnnibale VersariMarkus AlbertsmeierAnnica K. B. GadMarkus C. FleischAnnika AuranenMarkus ChristmannAnnika HeuerMarkus DiefenhardtAnnika LindblomMarkus J. AnkenbrandAnnika MalmströmMarkus MaeurerAnshika GoenkaMarkus NotterAnshu AgrawalMarkus S. JördensAnshuman PandaMarkus SchäferAnthony AudinoMarkus SchirmerAnthony CheungMarkus TiemannAnthony CostalesMarkus TschurtschenthalerAnthony EliasMarkus Vähä-KoskelaAnthony GloverMarkus Von DeimlingAnthony J. BerdisMarkus WilhelmiAnthony J. DixonMarlen HaderleinAnthony L. KovacMarlena GodlewskaAnthony M. C. BrownMarlene CervantesAnthony Michael HunterMarlene SantosAnthony TaylorMarley J. BinderAnthony TurpinMarlid Cruz-RamosAnthoula GaigneauxMarna E. EricsonAntien L. MooyaartMarry Van Den Heuvel-EibrinkAntimo MigliaccioMarshall E. KadinAntje GohlaMarshall WilliamsAntoine DurrbachMarta ArczewskaAntoine MarçaisMarta CostaAntoine NaemMarta Diaz-DelcastilloAntoinette HollestelleMarta DueñasAntonella ArgentieroMarta GomarascaAntonella BrunelloMarta I. LomnytskaAntonella CacchioneMarta KepinskaAntonella CusimanoMarta OpalińskaAntonella D'AnneoMarta OsrodekAntonella FarsettiMarta R. RomeroAntonella GozziniMarta ScorsettiAntonella ManniniMarta StarnoniAntonella PapaMarta Teixeira PintoAntonella RivaMarta TremoladaAntonella RosiMarta TruffiAntonella StravatoMarta WłodarczykAntonella TeramoMarta ZerunianAntonella TomassettiMartha Patricia Gallegos-ArreolaAntonello VidiriMartial GuillaudAntoni BenitoMartin AndrsAntoni M. SzczepanikMartin BakhtAntoni WiedlochaMartin BornhaeuserAntonia BusseMartin BöttcherAntonia Dimitrakopoulou-StraussMartin CorstenAntonia FollenziMartin DyerAntonia MancusoMartin EichlerAntonia ReimerMartin FaehlingAntonietta D’ErricoMartin FischerAntonino De PaoliMartin GaillardAntonino NatalelloMartin GonzalezAntonio AntónMartin GriesshammerAntonio AvalloneMartin HoheneggerAntonio BarileMartin HübnerAntonio BiondiMartin HufbauerAntonio CuneoMartin J. BakerAntonio CurtiMartin K. AngeleAntonio d'AmatiMartin KortümAntonio De NicolaMartin Kristian ThomsenAntonio FacciorussoMartin LehnertAntonio G. SolimandoMartin LeuAntonio GalanteMartin LovečekAntonio GonzalezMartin M. MortazaviAntonio IeniMartin MischAntonio Lopez-BeltranMartin MüllerAntonio M. Picó AlfonsoMartin MynarekAntonio MaccioMartin PéčAntonio ManentiMartin PrusinkiewiczAntonio MaraverMartin R. GillAntonio MatroneMartin R. HiggsAntonio MazzellaMartin RafteryAntonio MelcarneMartin Roelsgaard JakobsenAntonio MusioMartin RuthardtAntonio Pérez MartínezMartin Schmidt-HieberAntonio Piñero-MadronaMartin SchostakAntonio RaffoneMartin StannulaAntónio Roma-TorresMartin VojtekAntonio RussoMartin WeissAntonio SaccoMartin YaffeAntonio SalarMartin ZachariasAntonio ScilimatiMartin ZenkeAntonio SermekMartina AlvarezAntonio Simone LaganàMartina Anja BroglieAntonio TravaglinoMartina C. Herwig-CarlAntonio TufanoMartina FerioliAntonio ZanghiMartina KleberAntonios TzortzakakisMartina MaggiAntonis KourtidisMartina Messing-JüngerAntti Ilmari KoivusaloMartina MoriniAnudishi TyagiMartina SalákováAnuhya KommalapatiMartine BerliereAnuj AhujaMartine E. D. ChamuleauAnuja PandeMartino IntronaAnum MasoodMartyna Krzykawska-SerdaAnup KunduMarwa GodaAnup PathaniaMarwan KwokAnupam DhasmanaMary C. Farach-CarsonAnupam KotwalMary Garland-KledzikAnupam MukherjeeMary HendrixAnupama MunshiMary Lou CutlerAnuraag BoddupalliMary Lynn TrawickAnurag K. SinghMary McLeanAnurag ParanjapeMary SevignyAnurag SarafMaryam DaneshpourAnusha AdityaMaryam FarooquiAnusha AngajalaMaryna Krawczuk-RybakAnushka D. DongreMarzia Bruna GariboldiAnutosh GangulyMarzia Di DonatoAnvesh Reddy DasariMarzia OgnibeneAo ZhangMarzia PalmaAparna ShindeMarzieh SalimiAphrodite NonniMasaaki ItoApostolos C. AgrafiotisMasaaki KonishiApostolos Georgiou PappasMasabumi ShibuyaApostolos PapachristosMasaharu HataApostolos ZaravinosMasahide HiyoshiApril L. RisingerMasahide SaitoArantxa Carrasco-LeónMasahiro HiasaArantxa TaberneroMasahiro ImamuraAránzazu SánchezMasahiro ItonagaAranzazu VillasanteMasahiro JinzakiArcangelo SebastianelliMasahiro KagabuArco J. TeskeMasahiro NakayamaArdigo MarcoMasahiro YamamotoArghya RayMasahiro YashiArgiris S. SymeonidisMasahiro YasunagaArgyro MaziotiMasahito NakanoAria BaniahmadMasahito ShimojoArianna PalladiniMasakazu NotsuAriel JaffeMasaki KaiboriAriel Ramírez-LabradaMasaki KuwataniAriela NoyMasaki OhmurayaArif KhanMasaki ShiotaArijita SarkarMasako NakanishiArindam DattaMasakuni NoguchiArindam PramanikMasamitsu HyodoAris TsalouchosMasanao NakamuraAristeidis ChiotellisMasanori AbeAristidis TsatsakisMasanori FukushimaAristotelis BamiasMasanori KawakamiArjen R. MesenkampMasanori SaitoArkadiusz GertychMasao SaitohArkadiusz MiazekMasaru MiyazakiArko SenMasaru WakatsukiArman RahmanMasashi FukayamaArmand AbergelMasashi NinomiyaArmando Andres Roca SuarezMasataka KikuyamaArmando De VirgilioMasato YanagiArmando GenazzaniMasato YanoArmando PatrizioMasatomo KanekoArmando Pérez TorresMasatoshi WatanabeArmin PychaMasayo KagamiArmita BahramiMasayuki FujiwaraArnab BasuMasayuki KurosakiArnaldo StanzioneMasayuki MiyasakaArnaud AlvesMasayuki NoguchiArnaud GuilléMasayuki TsunekiArnaud JacquelMasoud AzodiArnaud MilletMasoud MadadelahiArnaud PasquerMassimiliano BerrettaArndt StahlerMassimiliano CretaArne WestgaardMassimiliano De PaolisArno GermondMassimiliano Del BeneArno KornbergMassimiliano Di PietroArnold PaulinoMassimo AgliettaArnoldo RiquelmeMassimo BarberisArnon NaglerMassimo BergerArnoud J. TempletonMassimo BonoraArora ShilpaMassimo BrogginiArosio MauraMassimo Di MaioArpita ChatterjeeMassimo FasanoArrigo CiceroMassimo GeunaArseniy E. YuzhalinMassimo La DedaArthi ShridharMassimo MaccagnoArthur FoulonMassimo OffidaniArthur FrankMassimo PapaleArthur RobertsMassimo RomaniArthur WagnerMassimo RuggeArtur BeberokMassimo TerzoloArtur LemińskiMassimo VicentiniArtur RebeloMassimo VitaleArtur SlomkaMassinissa YahiaArtur Y. PrilepskiiMassoud MirshahiArturs MeijersMasumi IshibashiA-Ru KimMasumi SuzuiArun AzadMateusz BujkoArun DharmarajanMateusz JankowskiArun SreekumarMateusz PsurskiAruna Kumar ChelluboyinaMateusz WierdakArvids IrmejsMathew BloomfieldArvind NegiMathew CherianArwin GroenewoudMathew PorunelloorArya AshokMathias KranzAsad UllahMathias TessonAsem SalmaMathias WorniAsgar ZaheerMathieu ChocryAshish AgrawalMathieu GautierAshish KabraMathilde C. M. KouwenhovenAshish ManneMathilde JalvingAshley M. EckelMatias A. AvilaAshok KumarMatias A. BustosAshton TheakstoneMatilde E. LleonartAsif RazaMatilde Yung FolloAsif ShajahanMatjaz RokavecAsit PaulMats EnlundAsma Ismail MahmodMats JohanssonAsmaa Fahmy KhafagaMatt SpickAsri AdisasmitaMatteo A. RussoAssunta TorneselloMatteo BarberisAsta JuzenieneMatteo BaucknehtAsterios PatsiaourasMatteo BolcatoAstrid GosewischMatteo CadossiAstrid LièvreMatteo CrippaAsunción Fernández-BarralMatteo De PastenaAtefeh ZarepourMatteo DonadonAthanasios ArmakolasMatteo FermiAthanasios G. PapavassiliouMatteo GhilliAthanasios T. PapadasMatteo RenzulliAthanasios TragiannidisMatteo RiccòAthina N. MarkouMatteo TruccoAtif Ali AhmedMatteo TurettaAtique Uddin AhmedMatteo VirdisAtit SilsirivanitMatthew A. TylerAtsushi FujimuraMatthew AhearneAtsushi MakimotoMatthew BeneschAtsushi MasamuneMatthew BottomleyAtsushi MotegiMatthew CasteloAtsushi SanoMatthew D. StachlerAtsushi TakaiMatthew DevallAtta NawabiMatthew E. CallAttila ZalatnaiMatthew H. RigbyAude JaryMatthew IbbsAudi Francesca SetiadiMatthew LewisAudrey Cougnard-GrégoireMatthew McCordAudrius DulskasMatthew McMillinAugustinas BausysMatthew P. GiannettiAung NaingMatthew PainschabAurelie DutourMatthew S. PainschabAurelie GarantMatthew SchlumbrechtAurélie RondonMatthew Stuart McKinneyAurélien VenaraMatthew T. HoudekAurora MaurizioMatthias GaestelAurora SavinoMatthias GoebelerAustin J. IovoliMatthias KapischkeAvik DuttaMatthias KraemerAvilala JanardhanMatthias LauthAvinoam NevlerMatthias MulazzaniAwatef KelatiMatthias NeesAxel GräwingholtMatthias S. LeisegangAxel KarowMatthias ScheweAxel PagenstecherMatthias SteinertAya T. ShalataMatthias StopeAyako MatsudaMatthias WilmAyaz ShahidMatthias WirthAydin EresenMatthieu FaronAydin GülsesMattia D’AgostinoAyelén ToroMattia Falchetto OstiAyelet ShaiMattia LauriolaAyman El-BazMattia RiefoloAymen IdrisMattia Russel PantaloneAyodeji Emmanuel IyandaMattias AnderssonAyse Günes-BayirMattias K. AnderssonAyse U. AkarcaMatus DurdikAysegul YildizMaud FriedenAyslan Castro BrantMaud ToulmondeAyuob AghanejadMaulik VyasAyush DograMaura MassiminoAzhar AliMaura MiccoAzhar ImranMaura PoliAzhar RasulMauricio J. ReginatoAzhar ZamMauricio MoraisAzra RazaMauricio Rodriguez-DorantesB. Ten HakenMaurício Temotheo Temotheo TavaresBabak NamiMaurizio AricòBabita Kumari VermaMaurizio CosimelliBachir BenarbaMaurizio MartiniBadr KiafMauro BelliBahil GhanimMauro BorzioBahriye AktasMauro CastroBailin ZhaoMauro ColucciaBalakrishna (Bal) L. LokeshwarMauro Giuseppe MastropasquaBalakrishna KoneruMauro PatroneBalamayooran TheivanthiranMauro Salvatore Alessandro AlaibacBalawant KumarMauro ValenteBalazs AcsMawieh A. HamadBalázs MargitMax M. WattenbergBalázs MayerMax NobisBálint TamaskovicsMax PetersBalraj SinghMax PiffouxBanghe ZhuMax SeidenstickerBanghyun LeeMax ZimmermannBanu ArunMaxim L. BychkovBaomin WangMaxime CahuzacBappaditya ChandraMaximilian BurgerBaptiste LouveauMaximilian ChristopeitBarak RosenzweigMaximilian DietrichBarani Kumar RajendranMaximilian Franz Schulze-HagenBarbara A. NiemeyerMaximilian J. MairBarbara BottazziMaximilian SelesBarbara BrunettiMaximilian WenigerBarbara BuldiniMaximiliano Tamae KakazuBarbara CanonicoMaxwell MartinBarbara CardinaliMay Ee PngBarbara DarišMaya SalehBarbara DziegielewskaMayank ChoubeyBarbara Garmy-SusiniMayumi KomineBarbara HardingMayur VirarkarBarbara JarząbMayura MeerangBarbara KoflerMazen A. JuratliBarbara LipińskaMazène HochaneBarbara MavroidiMaziar NikbergBárbara Paranhos CoelhoMcKale MontgomeryBarbara Ruszkowska-CiastekMd Afjalus SirajBarbara SeeligerMd Mamunur RahamanBarbara Sosnowska-PasiarskaMd MohiuddinBarbara SteccaMd Shariful IslamBarbara TestoniMd Zahid AkhterBardia YousefiMd. Hakimul HaqueBaris BoyrazMd. Imtaiyaz HassanBarry MilavetzMd. MushtaqueBart GhesquiereMd. RizwanullahBart NeynsMd. Sanower HossainBart Roelf Jan Van DijkenMeena JaggiBartek SzostakowskiMeena SakharkarBartosz BakMeenal DattaBartosz MalkiewiczMeera MohanBartosz PułaMeerambika MishraBartosz SzmydMegan A. MullinsBashir LawalMegan BeetchBassem HibaMegan DelisleBeata Filip-PsurskaMegan HitchinsBeata JabłońskaMegan SpurgeonBeata Kasztelan-SzczerbińskaMegha SinghalBeata MarciniakMeghan C. McCormickBeata PajakMeghan Caitlin ThompsonBeata SzymańskaMehdi BrahmiBeatrice AraminiMehdi KhaledBeatrice Gabriela IoanMehdi MontazerBeatrice MalacridaMehdi NassiriBeatrice ScazzocchioMehdi SyedBeatrice WindmöllerMehedi MasudBeatriz CarvalhoMehmet OzturkBeatriz GonzálezMehmet SarierBeatriz Perez-VillamilMehraju LoneBeatriz TaviraMehraneh D. JafariBegoña CánovasMehrdad JalaliBehnoush Abedi ArdekaniMehrdad RakaeeBehrooz H. YousefiMeicun YaoBehrus PuladiMeifang LiBejoy AbrahamMeijun DuBéla KajtárMeinrad BeerBela OzsvariMeiyun FanBelén LloverasMeizhu BaiBelgin SeverMejía CarmenBelinda BartonMelania BalasBelinda CampbellMelanie C. LangheinrichBelinda L. SunMelanie DeutschBen Chung-Lap ChanMélanie Di BenedettoBen SlotmanMelanie J. SekeresBen WylieMelanie MorrisonBenedetta GuaniMelanie PowisBenedict SeoMelanie WinkleBénédicte Brounais-Le-RoyerMelchor Sanchez-MartinezBenedikt FeuereckerMeliha EkinciBenedikt LinderMelinda FogarasiBengt GlimeliusMelinda PaholcsekBenita TamraziMelissa A. ReimersBenjamin AnsaMelita VidakovicBenjamin B. KastenMellar P. DavisBenjamin BeckMelpomeni KalofonouBenjamin BitlerMeng-Che HsiehBenjamin C. P. LeeMengze LyuBenjamin Chidi OnyeaguchaMeral YüceBenjamin DoornweerdMercedes GarayoaBenjamin FreyMercedes Medio-SimónBenjamin HeymanMercedes Mitjavila-CasanovasBenjamin KruppkeMerry L. TetefBenjamin M. Heyman HeymanMetka NovakBenjamin PinedaMeyling CheokBenjamin PurowMeysam KeshavarzBenjamin RybickiMhairi MorrisBenjamin SoibamMi Kyung ParkBenjamin TeplyMiao FeiBenjamin VerretMiao-Fen ChenBenjamin W PurowMichael A. DaneshvarBennett JulieMichael A. JacksonBenno WeigmannMichael A. JacobsBenny BjörkblomMichael A. KennedyBenoît MiottoMichael A. KieblerBenoit PaquetteMichael A. LeaBenoit ViolletMichael Anton BauerBerengere Pradet-BaladeMichael AreggerBergamino Sirven Milana ArantzaMichael BackBernard GallezMichael BartelsBernard HaendlerMichael BermanBernd KoelschMichael BlankBernhard B. SingerMichael BrownBernhard BiersackMichael C. SoulenBernhard G. ZimmermannMichael CangkramaBernhard GebauerMichael ChoiBernhard H. RauchMichael D. DiamantidisBernhard MeyerMichael DeanBernhard Michael PolzerMichael DeCuypereBernhard SchreflerMichael EwerBernhard WernlyMichael FleischhackerBertram WiedenmannMichael FrühwaldBertrand BaujatMichael GötzBertrand LiagreMichael GreenBertrand RoutyMichael GruschBethany N. SmithMichael HahneBettina WinzelerMichael HausmannBeverly LongMichael HottigerBhakti PatelMichael HundemerBhalchandra MirlekarMichael I. KoukourakisBharani Krishna Mynampati ArunadithyaMichael J. EbleBharath Kumar VelMichael J. KaplanBharath RamaswamyMichael J. MonumentBhavna MuraliMichael J. NathensonBhawna DevMichael J. WagnerBhoomika PatelMichael KahnBhupal P. BhetwalMichael KnitschkeBhuvana SettyMichael KoukourakisBhuvanesh Sukhlal KalalMichael KrachtBhuvic PatelMichael KrainerBiagio BaroneMichael KuehlBianca BrixMichael L. WangBianca GoemansMichael LadomeryBianca Maria ZaniMichael LathamBianca NitzscheMichael LeeBianca PolloMichael LerchBiancastella CereserMichael MichaelBibha ChoudharyMichael MintsBijun ZengMichael MorganBikash PattnaikMichael MuetherBilal A. SiddiquiMichael N. DworzakBilal HafeezMichael P. MarkeyBiliana NikolovaMichaël PérèsBin GuMichaël PerraisBin QiuMichael PfeilstöckerBin S. TehMichael PinkawaBin SongMichael PoellmannBin WangMichael R. CragerBing XiaMichael R. DeyhleBingdi ChenMichael R. PoulsonBinglan YuMichael RiedBingsheng HuangMichael RoseBingyun SunMichael S. JacksonBinita ShresthaMichael SchmittBinlin WuMichael SchwakeBinu Kandathilparambil SasiMichael ShurinBiplab DasguptaMichael SimBirgit LohbergerMichael SpinellaBirgitte OlsenMichael T. KoltzBirija Sankar PatroMichael TaylorBishal PaudelMichael TellierBiswarup SahaMichael TomassonBjörn GyllingMichael V. OrtizBjørn NaumeMichael VoulgarelisBlanca Estela García-PérezMichael W. BishopBlanca Lumbreras LacarraMichael WeichhausBlanka KubesovaMichael WelshBlas LeandroMichael WyattBlas Lotina-HennsenMichael YingBo WangMichaela RamserBoban M. ErovicMichail KostaresBobby DasariMichail SorotosBobin ChenMichal AndrlikBocheng WuMichał BorysBofan SongMichał DąbrowskiBogdan KazmierczakMichal DmowskiBogdan PinteaMichal GoldbergBogdan Silviu UngureanuMichal JurášekBogdan SoceaMichal KolářBogusław MikaszewskiMichał KulusBogusz KulawiakMichal Laufer-PerlBojan MitićMichal OrdakBojan MusilMichal SchweigerBojan ZaricMichal Shoshkes-CarmelBojana MilutinovicMichal SmahelBoleslaw T. KarwowskiMichal ŠmídaBonald FigueiredoMichal WozniakBoon Huat BayMichał ZałȩckiBoris B. NiraulaMichal ZarobkiewiczBoris BilcikMichał ŻorniakBoris GuiuMichał ŻurekBoris GuyotMichalis LiontosBoris SepesiMichel C. Henry-AmarBoris SlobodinMichel GonzalezBoris TenchovMichel RigaudBorislav A. AlexievMichel ToledanoBorislav AngelovMichela CorsiniBorja Herrero De La ParteMichela FerrucciBoštjan MarkelcMichela PozzobonBožena Ćurko-CofekMichele AizenbergBozena KaminskaMichele BartolettiBożena Romanowska-DixonMichele Christian KlymiukBradley R. CorrMichele Dei CasBradley TurnerMichele Di CosolaBrandon G. SmagloMichele FinottiBrandon M. HuffmanMichele FioreBranka Petrić-MišeMichele GraciottiBrankica FilipicMichele GuidaBranko CuglievanMichele KlainBrant G. WangMichele LonghiBrendon CoventryMichele ManganelliBrent A. StanfieldMichele MarchioniBrent WeinbergMichele MazzantiBrian BoothMichele MazzolaBrian BrownMichele NavarraBrian D. AdamsMichele PaganoBrian GabrielliMichele PietragallaBrian KøsterMichele RussoBrian R. DavidsonMichele S. RedellBrian Van TineMichele SamajaBrianl BadgwelMichel-Edwar MickaelBrie KezlarianMichelle A. EspyBrigette Buig Yue MaMichelle AfkhamiBrigida MaioranoMichelle CoriddiBrigitta G. BaumertMichelle GhertBrigitte F. M. SlangenMichelle GreeneBrigitte GatterbauerMichiel J. P. SedelaarBrigitte HantuschMichiel KroesenBrigitte MarianMichiel N. KerstensBrigitte WilczekMichihiro MutohBrijesh KumarMichio OtsukiBritta KleinMicol Silic-BenussiBrittany DewdneyMielnik MichalBrittany MurphyMiguel Angel Berciano-GuerreroBrittney S. HarringtonMiguel Angel DiazBruce M. BomanMiguel Ángel JapónBruce RumboldMiguel Angel Molina VilaBruce SmithMiguel Ángel Rodríguez-MillaBruna PelliniMiguel Ángel Suárez-MuñozBruna ScaggianteMiguel E. Aguado-BarreraBruno António Caetano CardosoMiguel González MolesBruno ClémentMiguel Martin-CaraballoBruno FiondaMiguel Mascarenhas SaraivaBruno FuchsMiguel Quintela-FandinoBruno Hebling VieiraMiguel Sánchez ÁlvarezBruno José Nievas SorianoMiguel SanzBruno MärklMihaela Flavia AvramBruno PontesMihaela MocanBruno R. B. PiresMihai CapilnaBruno RaynardMihai DanciuBryan WongMihai Dorin VartolomeiBuckley McCallMihai LupuBuddhadev LayekMihai-Teodor GeorgescuBulat RamazanovMihaly CserepesBurkhard BrandtMihaly MezeiBurkhard KleuserMi-Heon RyuBurra MadhukarMihir ShettyByung Soo KimMihnea DragomirByungwook MinMihnea-Alexandru GămanC. Chris YunMiho KawaidaC. James ChouMiikka LehtonenCaecilia H. C. SukowatiMikael ErikssonCai M. RobertsMikael S. LindströmCălin CăinapMikalai BudzevichCalogero CasàMike-Andrew WesthoffCalogero VetroMikhail N. BerlovCamelia CoadaMikhila MahendraCamelia QuekMikio ShimadaCameron BrackenMikko J. LoukovaaraCameron K. TebbiMilad RasouliCameron KochMilda RudzianskieneCamilla PecoraroMilena CavicCamille VerryMilena DeptułaCan Fahrettin KoyuncuMilena GeorgievaCaner SayginMilena NicolosoCara B. GonzalesMilena ŚwitońskaCara-lynne SchengrundMilica MarkovicCaridad Marín-HernándezMilica PešićCarina DehnerMiloslav MachacekCarina Lundh HagelinMilton KanashiroCarl FisherMin Heui YooCarl Friedrich ClassenMin PengCarla De GiovanniMin ShiCarla Di StefanoMin WuCarla FinkielsteinMina ZeinaliCarla H. Van GilsMinakshi GandhiCarla LoretoMineko TeraoCarla Maria Batista Ferreira PiresMineyoshi AoyamaCarla MucignatMing Hsien ChanCarla PalmaMing YangCarla PisaniMing YaoCarla RaggiMingang HaoCarla Solé CañadasMing-Chao TsaiCarlie De VriesMing-Chin YuCarlo Alberto MaroneseMingchung ChouCarlo Antonio BaroneMing-Derg LaiCarlo Cosimo CampaMing-Fen LeeCarlo GaniniMingfu ChenCarlo GrecoMing-Hsien ChanCarlo IraceMing-Hsin YangCarlo LajoloMing-Jen LeeCarlo MessinaMingjian James YouCarlo MorassoMingli LiCarlo PerisanoMing-Lung YuCarlo RodellaMingsheng ChenCarlo RodolfoMing-Shiou JanCarlo RonsiniMingte Tegh HuangCarlo SaittaMingyi ChenCarlo TrombettaMing-Yii HuangCarlo V. CatapanoMinhhuyen T. NguyenCarlos Alberto ScrideliMinhong ShenCarlos BarreroMin-Hsien ChiangCarlos CaroMinjun YangCarlos CasianoMinna PiipponenCarlos De NoronhaMinoru NagayamaCarlos Eduardo Fonseca-AlvesMinsong CaoCarlos GalanMinsu KwonCarlos L. Cespedes AcuñaMiodrag GužvićCarlos MarcuelloMi-Ran ChoiCarlos Martin LlorenteMiran RadaCarlos MartinezMircea-Catalin FortofoiuCarlos Martínez-BoubetaMirco CastoldiCarlos Martinez-CampaMirco MasiCarlos Navarro CuéllarMirco SchapherCarlos P. RubbiMireia Crispin-OrtuzarCarlos P. SosaMireia OlivanCarlos Pardo-PastorMirella GiovarelliCarlos Perez-StableMirella TanoriCarlos PlacerMiriam ButtacavoliCarlos Plaza-SirventMiriam CuatrecasasCarlos Rodriguez-NogalesMiriam GaggianesiCarlos S. MorenoMiriam Keltz PomeranzCarlos SebastianMiriam MarquésCarlotta GiorgiMiriam MenzelCarmela De SantoMiriam PotronyCarmelina C. ZirafaMirjam GerwingCarmelo CaldarellaMirjana KesslerCarmelo FerraiMirko H. H. SchmidtCarmelo MilitelloMirko MaturiCarmen AbateMirna Mourtada-MaarabouniCarmen BerasainMiroslav ChovanecCarmen DomínguezMiroslaw GiurgCarmen Fiuza-LucesMiroslawa CichorekCarmen Griñán-LisónMiroslawa PuskulluogluCarmen HöglingerMirunalini RavichandranCarmen Lacramioara ZamfirMirza PojskicCarmen NeamtuMirza PojskićCarmen Ramírez-CastillejoMischa De RidderCarmen RuizMistuhisa TakatsukiCarmen Sorina MartinMitchell FaneCarmine PiconeMitchell KamravaCarmine PintoMitesh PatelCarmine SelleriMithalesh Kumar SinghCarminia Maria Della CorteMitomu KioiCarol C. ShouldersMitsuaki IshidaCarol ImbrianoMitsuko MasutaniCarol LeungMitsumasa OsakabeCarol SartoriusMitsuru NishiyamaCarol WadhamMizue TeraiCarola Lütgendorf-CaucigMizuho NishioCarole BouleucMizuho Sato-DahlmanCarole HelisseyMizuki AzumaCarole SousaMladen ParadžikCarolin KastnerMohamad H. QariCarolin KnebelMohamad M. Al-RahawanCarolina Alonso-GonzálezMohamed A. TammamCarolina D. SchinkeMohamed AbdelgiedCarolina MensiMohamed Abou El-GharCarolina NylenMohamed Faisal ChevidikunnanCarolina SalvadorMohamed GadelkarimCarolina SandlerMohamed HassanCarolina SimioniMohamed Raafat El-GewelyCarolina SolomonMohamed SallaCarolina TorresMohamed Z. GadCarolina UngMohammad A. SaadCaroline BessonMohammad AliCaroline C. C. HulskerMohammad AlkhaleefahCaroline GronnierMohammad AlWahshCaroline HertlerMohammad AlzrigatCaroline HimbertMohammad Amin MoosaviCaroline L. HollowayMohammad BagherniyaCaroline LindemansMohammad DabaghiCaroline M. Den HoedMohammad Esmaeil AkbariCaroline PietteMohammad Faujul KabirCaroline StokkeMohammad Hassan HodrojCaroline TruntzerMohammad Imran KhanCarolyn HeckmanMohammad JavanbakhtCarolyn NessimMohammad KamranCarolyn OwenMohammad KeilaniCarpén TimoMohammad KhalidCarrie HouseMohammad MohammadianpanahCarsten CarlbergMohammad Mohiminul IslamCarsten FriedrichMohammad Naghavi-BehzadCarsten StephanMohammad SouriCasey LangdonMohammad TariqueCasimiro GerarduzziMohammad Usman AhmadCasimiro Luca GigliottiMohammed AlaswadCasper Van EijckMohammed AlbatanyCassandra J. VandenbergMohammed El-MowafyCassiano Felippe Gonçalves-de-AlbuquerqueMohammed H. MohammedCataldo PignatelliMohammed HawashCatalin BulaiMohammed Kashani-SabetCătălin Ioan VladMohammed Muqtader AhmedCatalin MarianMohd Ahmar RaufCatarina Luís SilvaMohd FarhanCatarina Roma-RodriguesMohd ImranCaterina BartolacciMohit Kumar JollyCaterina BonfiglioMohit SharmaCaterina MarchiòMohsen KeshavarzCatherine ApplegateMohsen YazdanianCatherine CoombsMohsin MaqboolCatherine GodfraindMohsina KhanCatherine J. SinnottMoiz VoraCatherine TomasettoMojca PavlinCátia DominguesMojun ZhuCatia GiovanniniMolishree Umesh JoshiCatia MorelliMolly L BristolCecilia AriciMomcilo JankovicCecilia BindaMona BatishCecilia MariniMona El-BahrawyCecilia Matilde Fernández PonceMona K. JomaaCecilia PozziMona Kamal SaadeldinCédric DelevoyeMona P. TanCédric PoleselloMona ShehataCedric TremblayMonde NtwasaCeleste CagnazzoMonglien WangCeleste Caruso BavisottoMonia LenziCeleste ManfrediMonia MarchettiCelestial T. YapMonica BalzarottiCelina Su-Ping AngMonica FedeleCéline S. GonçalvesMónica Lamas MaceirasCelso Dario RamosMonica Livia GheorghiuCelso ReisMónica Martínez-FernándezCemil ColakMonica NigerCen WuMonica TerenzianiCen ZhangMonica YabalCésar López-CamarilloMonika BarathovaCeshi ChenMonika HämmerleChai Hong RimMonika Klinkhammer-SchalkeChakraborty SohiniMonika KujdowiczChamini J. PereraMonika LejmanChandra MishraMonika Pazgan-SimonChandrama MukherjeeMonika PiweckaChandramani PathakMonika PizonChandrasekhar YadavalliMonika PrendeckaChandrashekhar HonraoMonika RuszałaChandrika GowdaMonika Sakowicz- BurkiewiczChang Dong YeoMonika ScheerChang GongMonika SobiechChang Hyun KangMonika SzeligaChang Ik YoonMonika UlamecChang Ok SuhMonique J. RoobolChangchuan JiangMonique Petrousjka Van Den TolChang-Han ChenMonireh Sadat SeyyedsalehiChang-Hoon ChoiMonish Ram MakenaChang-I ChenMonsurat LawalChang-Shen LinMontserrat MaríChangwei LinMontserrat ReyesChangying JiangMontserrat SolerChanpreet SinghMoonkyoo KongChan-Yen KuoMoonsun JungChao LiangMoran AmitChao WangMoran GadotChao ZhangMorgan MclemoreChaofan LiMorgane Dos SantosChaopu TiMorito KurataChaowalit YuajitMoritz F. EissmannChao-Yang ChenMoritz JesinghausChao-Yie YangMorohoshi TakaoChao-Yu ChenMorten LadekarlChara StavrakaMorteza KafshgariCharalambos AndreadisMoshe ElkabetsCharalampos ThomasMoshe GattCharis LiapiMostafa A. AbooufCharles A. PowellMostafa DianatinasabCharles BaileyMouadh BarbirouCharles DumontetMoumita DattaCharles EberhartMourad ZerfaouiCharles GiardinaMoutih RafeiCharles GuoMubarak MashrahCharles L. WilkinsMudassar Iqbal ArainCharles LawrieMueez WaqarCharles NemeroffMuhamad Noor Alfarizal KamarudinCharles RosserMuhammad A. RahmanCharles S. CobbsMuhammad Ayaz AnwarCharles TheilletMuhammad Fazal IjazCharles TongMuhammad IqhrammullahCharles TruilletMuhammad KhanCharles-Henri MalbertMuhammad KibriyaCharlotta WadstenMuhammad Muzzammil EdhiCharlotte GrégoireMuhammad N. MahmoodCharlotte Kelley-JonesMuhammad Nadeem AslamCharlotte LawsonMuhammad R. HaqueCharlotte MaulatMuhammad SaleemCharmaine A. Ramlogan-SteelMuhammad Umair AliCharu GuptaMuhammad UmerCharu KothariMuhammad Yasir AsgharCharupong SaengboonmeeMuhammet SayanChava PerryMu-Kuan ChenChe-Chang ChangMukul K. DivatiaChe-Hsin LeeMukund BhandariChe-Hsueh YangMumtaz AnwarChellappagounder ThangavelMumtaz RojianiChe-Ming Jack HuMunendra TomarChen Guo KerMunirathinam GnanasekarChen-Ching LinMurali JanakiramCheng GuoMurali MamidiCheng Hsien WuMurali MunirajuCheng WangMuralidharan ManiCheng XueMurat AladaǧChengcheng ZhangMurat GökdenCheng-Chia LeeMurat SaparbaevChengfeng YangMuriel Le RomancerCheng-Hsu WangMuriel MathonnetCheng-Hsun HoMusa YilmazChenghui LiMustafa JalalChenghung LinMustafa S. AschaCheng-Maw HoMustafa Zelal MuallemChengquan ZhaoMuşturay KarçaaltıncabaCheng-Yang ChouMutamed AyyashCheng-Ying ChuMuthukumaran VenkatachalapathyChengyue WuMuyun XuChenhan ZhongMuzamil WantChenhua LiuMyriam CuadradoChenjian GuMyron R. SzewczukChen-Xiong HsuN. Scott LitofskyCherie-Ann NathanNaama KanarekChhanda BoseNabil A. HasonaChi Chiu WangNabil NicolasChi Chun WongNada ChaoulChi Kong LiNada HamadChi MaNadège KindtChi Man TsangNader K. FrancisChi Tung ChoyNader MeskinChiachen KuNadezhda GermanChia-Feng LuNadia BrancatiChia-Hung HsiehNadia Di MuzioChia-Hung YenNadia Judith Jacobo-HerreraChia-Hwa LeeNadia KhanChia-Jung LiNadia Sawicka-GutajChiaki TakahashiNadine GervoisChia-Li HanNadine HouédéChiao-En WuNadine MallakChiara AdamiNadine S. AguileraChiara AgostinisNadine WiesmannChiara CarlomagnoNadja EngelChiara CastelliNadya I. TarasovaChiara DoniniNae-Cherng YangChiara GiraudoNaeem SaleemChiara MassaNaga Babu ChinnamChiara MazziottaNagavendra KommineniChiara MoltrasioNagesh KolishettiChiara NapoletanoNageswari YarravarapuChiara PaganelliNagi B. KumarChiara QuarnetiNagy AkosChiara SpadazziNahid AliChiara VitaleNaiba NabievaChiara VolpiNaihan XuChia-Yen HuangNai-jung ChiangChi-Chang YuNai-Ki MakChie InomotoNaila BobyChie KojimaNaila Francis Paulo De De OliveiraChieko AzumaNai-Yun SunChien-Chang KaoNalu Navarro-AlvarezChien-Chen LaiNam Deuk KimChien-Chin ChenNam-gu HerChien-Feng LiNamratha SheshadriChienhung ChenNan SethakornChien-Hung ChenNan ZhangChien-Hung LinNancy Adriana Espinoza-SánchezChien-Min LinNancy GavertChihaya KoriyamaNand RoyChih-Cheng LaiNandaraj TayeChih-Chin YangNandini KrishnamoorthyChi-Heum ChoNan-Shan ChangChih-Hsi Scott KuoNao FujimoriChih-Hsing ChouNao KakizawaChih-Hsiung HsuNaofumi AsanoChih-Jen YangNaohiko SekiChih-Ping HsuNaohiro FujimotoChih-Yang WangNaoki FujitaChi-Ju KimNaoki FuruyaChileung ChiangNaoki ItanoChi-Long ChenNaoki KanekoChi-Ming WongNaoki KawagishiChin Ann Johnny OngNaoki TeradaChin-Chen ChuNaoki UmemuraChin-Chou WangNaoki YamamotoChi-Neu TsaiNaomi FujiokaChing-Chung LinNaoya NakamuraChing-Feng ChiuNaoya ShikazonoChinghsiung LinNaoya TadaChing-Sheng HsuNaoyuki KataokaChin-Mu HsuNarayan ShivapurkarChinten J. LimNardin SamuelChin-Yo LinNarendar DudhipalaChi-Ping DayNarendranath EpperlaChiranjeevi PadalaNaresh PoondlaChiranji Lal ChowdharyNarikazu BokuChit TamNaris NilubolChitra JosephNarongchai AutsavaprompornChiung-Fang ChangNassim Ghaffari-Tabrizi-WizsyChiung-Ting WuNatacha D. EmersonChi-Wen LuoNatacha Entz-WerléChong-Chi ChiuNatalia A. IgnatenkoChonlaphat SukasemNatalia AbramenkoChoong Yong UngNatália AraújoChoong-kun LeeNatalia Díaz-ValdiviaChris H. J. TerhaardNatalia DrabińskaChris MontenNatalia FilippovaChris PlanqueNatalia GenereChris W. SuttonNatalia GrubaChrissa SiokaNatalia KasicaChristel HedmanNatalia MotasChristelle BouchartNatalia RakislovaChristian A. GutschowNatalia Riobo-Del GaldoChristian BaillyNatalia SimionescuChristian BeltzerNatalia Yanguas-CasásChristian BerthouNatalie GroverChristian GrohéNatalija FilipovićChristian KerstenNataliya MarChristian KosanNatalya A. GloushankovaChristian L. LaboisseNatalya V. YunusovaChristian LaboisseNatapol PornputtapongChristian M. SchürchNatarajan SuganthyChristian MawrinNatasa MilicChristian PirichNatasa PavlovicChristian ScheiweNatasa ZarovniChristian SchindlbeckNatasha Pillay SmileyChristian Von BuchwaldNatasha T. HillChristian WirajaNatércia ConceiçãoChristiane A. OpitzNathalie AndréChristin B. DeStefanoNathalie Auphan-AnezinChristina GrimmNathalie Baeza-KalleeChristina HensonNathalie BockChristina J. GutowskiNathalie Britzen-LaurentChristina MarneyNathalie TheretChristina MullinsNathan BergerChristina SignorelliNathan GluckChristine BezombesNathan H. ParkerChristine BlattnerNathan LanningChristine E. ShefferNathaniel WeygantChristine GillesNatsumi FujiwaraChristine Le RoyNattika SaengkritChristine MarosiNava Siegelmann-DanieliChristine ToedebuschNaveena Basa JanakiramChristo KoleNavid AbedpoorChristof VulstekeNavid RazmjooyChristoph AlexiouNavid SobhaniChristoph Ammer-HerrmenauNavin ViswakarmaChristoph GrimmNayoung KimChristoph HoellerNayun KimChristoph JochumNazefah Abdul HamidChristoph KornauthNebojsa S. ManojlovicChristoph N. SeubertNeda Haj-HosseiniChristoph ReissfelderNeda SladeChristoph RölligNeeladrisingha DasChristoph RomaninNeenu SinghChristoph StallmannNeeraj ChauhanChristoph ThomssenNeeraj JainChristoph WohlmuthNeeti SwarupChristophe DeroanneNeetu SinghChristophe DesterkeNegroiu GabrielaChristopher BallmannNeha DesaiChristopher BoehlkeNeha NandaChristopher BonifaceNeil A. BhowmickChristopher DarrNeil D. MerrettChristopher E. WeeNeil H. BanderChristopher GrothNeil McRobertsChristopher HeaphyNeil R. MillerChristopher I. AmosNela MalatestiChristopher J. PinardNelida Ines NogueraChristopher J. RickettsNelofer SyedChristopher J. YoungNelson TangChristopher John PepperNelson YeeChristopher KobierzyckiNemany HanafyChristopher LucasNemat AliChristopher M. JesseNessr RachedChristopher ManssonNéstor MartínezChristopher MoertelNethaji MunirajChristopher N. HahnNeville HackerChristopher Nevala-PlagemannNezahat HunterChristopher PlaisierNg Chau Hsien MatthewChristopher R. J. WoodhouseNgu TrinhChristopher R. SheaNguyen Minh DucChristopher S. WilliamsNibedita PatelChristopher T. WilkeNic M. GillingsChristos ChouaidNic OrsiChristos K. KontosNiccola FunelChristos MikropoulosNiccolò GallioChristudas MoraisNicholas BrownChuangyu WenNicholas J. ShortChuanjin WuNicholas PeakeChukwuka EzeNicholas R. HumChun YuNicholas W. FischerChun-Chia ChengNicholas WallaceChun-Chih TsengNicholas ZorkoChundong YuNick KouttabChung-Chun WuNicklas Heine StaunstrupChung-Jung LiuNico RuprechtChun-Han ChenNicol StrakováChunhong YanNicola AnnelsChun-Hou LiaoNicola ArmstrongChunhui HanNicola Bougen-ZhukovChunkai LiaoNicola CarterChun-Liang ChenNicola Di RenzoChunming ChengNicola FuscoChun-Ru ChienNicola IngramChunsheng KangNicola LongoChun-Song YangNicola Luigi BragazziChun-Te WuNicola MaggialettiChunxiao LiuNicola MarottaChunyan DongNicola MaureaChunyan WangNicola MicaleChunyi WuNicola MontemurroChunying LiNicola NicolaiChunyu CaiNicola PederneschiChun-Yu LinNicola PersoneniChun-Yu LiuNicola SgherzaChunyuan JinNicola Stefano FracchiollaChutima KunacheewaNicola TinariChyong-Huey LaiNicola VeroneseCiara O’FlanaganNicola WaddellCicone FrancescoNicola ZingarettiCiesla MaciejNicolae BacalbaşaCihan AyNicolae GicaCindy KörnerNicolai E. SavaskanCindy YangNicolas AlbinCinzia AntognelliNicolas ChapelleCinzia BagalàNicolas ClereCinzia GarofaloNicolas DulphyCinzia GiacomettiNicolas DumazCinzia LanziNicolas ForayCinzia OrtegaNicolas GolseCiprian TomuleasaNicolas JonckheereCiriaco CarruNicolas SayeghCiro ImbimboNicolas TorresCiro RinaldiNicole A. SeebacherClæs E. MerckeNicole BrossierClaes MerckeNicole C. SchmittClaire AcquavivaNicole D. BouvyClaire D. JamesNicole SalazarClaire DelbridgeNicole TerpolilliClaire F. VerschraegenNicole Van GriekenClaire FalandryNicole WittwerClaire M. DuboisNicoleta AntonClaire R. PeghaireNicoletta CordaniClaire RoddieNicolò CardobiClaire RooneyNidhi SharmaClaire WalczakNiels F. M. KokClara Cieza-BorrellaNiels ReinmuthClare L. ScottNienke E. Van TrommelClaro BichoNieves PeltzerClaude Caron De FromentelNigel J. AndersonClaude Van CampenhoutNik TsotakosClaudia AfferniNikee AwastheeClaudia ArndtNikhil P. JoshiClaudia CantoniNikhil VasdevClaudia CavaNiki ChristouClaudia CortiNiki M. Zacharias MillwardClaudia GravekampNikita NikitaClaudia HaferlachNikki CopelandClaudia MaletzkiNiklas Von SpreckelsenClaudia MiniciNiklas WesthoffClaudia Ollauri-IbáñezNiko NjiricClaudia PeitzschNikol SulloClaudia PivonelloNikola AnđelićClaudia ScottiNikola BesicClaudia SorbiNikola JovanovićClaudia TestaNikola PanićClaudia TregnagoNikola SkoroClaudia VolpiNikolai SimonovClaudine KiedaNikolai TimchenkoClaudio FeoNikolaos A. AfratisClaudio GambardellaNikolaos AndreatosClaudio LuchiniNikolaos E. PatsoukisClaudio MaiaNikolaos KatzilakisClaudio PedoneNikolaos MachairasClaudio SpinelliNikolaos NazirisClaudio TabolacciNikolaos PapandrianosClaudiu MorgovanNikolaos PistamaltzianClay J. CockerellNikolay MehterovClélia CoutzacNikolay Ul'yanovskiyClemens B. TempferNikolina StojanovićClemens KaiserNikos NikolettosClemens RosenbaumNiloufer KhanClemens ZwergelNils LudwigClément MorgatNimi MarcelClément ThomasNimi VashiClement YuenNimrat ChatterjeeCleo-Aron WeisNina CorteseCleverton De AndradeNina GaleClodia OsipoNina KurrleCoco C. H. De KoningNina MilevojCodruta SoicaNina ModerauCody NesvickNina MontikCody PeerNina RembiałkowskaCoen R. N. RaschNina ReuvenColin H. QuinnNina Rosa NeuendorffColin KennyNina ZidarColin LindsayNing JinColin SteeleNing YangColin WattsNing-Ning LiColm A. O'MorainNir Ben-ChetritColm O'RourkeNiraj K. NiralaConcetta AltamuraNiraj LodhiCondorelli GerolamaNirmala MavilaCong PengNirmalya SahaConsiglia PacelliNisar Ul KhaliqConstantin N. BaxevanisNisha D'SilvaConstantina AggeliNishanth NairConstantino Carlos Reyes-AldasoroNithyananthan SubramaniyamConstantinos StathopoulosNitikaConstantinos T. SofocleousNitin RoperConsuelo GuardiolaNitin ShiroleConsuelo TorriniNitin TelangCorina Kim-FuchsNitish GulveCorinna SeligerNityanand SrivastavaCornelia AmalineiNitzan ZoharCorrado CasliniNnennaya KanuCorrado PedrazzaniNobuaki HigashiCorrado TinterriNobuhiro OobaCosimo CumboNobukazu FujimotoCosimo SpertiNobuo KondohCosmin EneNobuyoshi FukumitsuCostica CaizerNobuyoshi HiraokaCourtney FultonNobuyuki HoritaCraig DerkayNobuyuki KuribayashiCraig ErkerNoélia CustódioCraig HorbinskiNoemi LaproviteraCraig MeyersNoemí PuigCraig N. Craig RobsonNoemi ScarpatoCrescenzo D’AlterioNoha M. ElemamCrismita DmelloNokitaka SetsuCristian AxenieNoopur ThakurCristian FurauNoor Al-Huda Ali A. H. SaeedCristian I. SurcelNootan PandeyCristian Ionut OrasanuNor Eddine SounniCristian ManzoniNora KatabiCristian MirvaldNorbert GrafCristian PersuNorbert KissCristian Prieto-GarciaNorbert MarschnerCristian ScheauNoriaki SasaiCristian TorresNoriaki SunagaCristiana CarnitiNoriaki TomuraCristiana. BellanNoriaki YoshidaCristian-Radu JecanNorifumi NakamuraCristina AndreaniNorihiko NaritaCristina AndreoliNorikazu MasudaCristina B. HebedaNoriko KishiCristina CapatinaNoriko TakegaharaCristina CaramesNorio MiyoshiCristina CasalouNorio NonomuraCristina De Oliveira Massoco Salles GomesNorio SogawaCristina FalciNoriyoshi SawabataCristina FillatNoriyuki OkonogiCristina FornagueraNorma F. KanarekCristina GhiciucNorma-Aurea Rangel-VázquezCristina GluhovschiNoureddine IssaouiCristina JoaoNunzia MatroneCristina L. RonchiNupur DasCristina Lirio RomeroNur ZeinomarCristina LópezNuria KoteckiCristina MaccalliniNuria Maria NovoaCristina MambetNuria Mulet-MargalefCristina Martin-SabrosoNury Pérez-HernándezCristina Montiel-DuarteNyall LondonCristina PanuzzoOana Cristina ArghirCristina PenasOana-Maria ThomaCristina SegoviaOctavian EnciuCrnogorac Marija DjordjicOctavian NeagoeCsaba SzaboOfer Nathan GofritCsilla PesznyákOhmori YoshihiroCsongor KissOke GerkeCtirad HofrOkyaz EminagaCushla McKinneyOladapo BakareCyril SobolewskiOlaf GefellerCyrille ConfavreuxOlaf HardtCyrille De JoussineauOlaf Johan Hartmann-JohnsenCyrus MotamedOlaf MerkelD. S. PrabakaranOlav YriDaan DierickxOleg BlyussDadgar MeysamOleg ShuvalovDae Ho LeeOleg TimofeevDaekyu SunOleksii TyshchenkoDafna BenayahuOlga A. GuryanovaDafne Campigli Di GiammartinoOlga AbiánDagmar RiemannOlga AnatskayaDagmar StoiberOlga BegouDagmar Von BubnoffOlga BrovkinaDagmara Szmajda-KrygierOlga BureninaDahang YuOlga CherkasovaDai ChiharaOlga D. ZakharovaDaishi YamakawaOlga GoloubevaDaisuke HokutoOlga GordeevaDaisuke HoshinoOlga KorczeniewskaDaisuke KawaharaOlga KovalevaDaisuke KomuraOlga KrizanovaDaisuke MiyoshiOlga Lilia Garibay-CerdenaresDaisuke OgiyaOlga MalyshevaDaisuke TakahashiOlga S. FedorovaDaisuke TakayanagiOlga Stenina AdognraviDaisuke YoshidaOlga VinogradovaDaitoku SakamuroOlga Warszawik-HendzelDakang XuOlga Y. KorolkovaDaley S. MoreraOliver BatheDalia Baršytė-LovejoyOliver BouchéDa-Liang OuOliver GimmDalin ZhangOliver GrauerDalsan YouOliver HaaseDaly AvendanoOliver HakenbergDamiano CaputoOliver HanemannDamiano CarusoOliver KeppDamien A. LeachOliver OttDamir VareslijaOlivia Francis-BoyleDamu TangOlivia PaganiDan CanaaniOlivia SgarburaDan ChenOlivier BindaDan G. DudaOlivier CalvayracDan GranbergOlivier DarbinDan LiOlivier DormondDan QiOlivier GadalDan YanOlivier GiresDana CucuOlivier HuillardDana FerrarisOlivier PeyronyDana HartlOlivier RiouDana Lucia StănculeanuOlivier SaulnierDana Maria CopoloviciOlle RingdénDana R. CrawfordOlli M. J. DufvaDana Rodica TomescuOlli SilvennoinenDana StoianOluseye OgunmorotiDanai FimereliOmanma AdighibeDandan ZhengOmar FrancoDangdang LiOmar HamdyDanh D. TruongOmero Bendicto Poli-NetoDania MoviaOmid AzimzadehDaniel A. WeiserOmran SaifiDaniel Andrei IordanOon Ein ChernDaniel B. StovallOra IsraelDaniel BilbaoOrawit ThinnukoolDaniel BreadnerOreste GalloDaniel C. ChristophOriol De BarriosDaniel CatchpooleOrla SheilsDaniel E. FrigoOrlando Guntinas-LichiusDaniel EigerOrlando MussoDaniel EyraudOrsola Di MartinoDaniel FinkeOsagiede OsayandeDaniel GeorgeOsamu IshibashiDaniel GómezOsbaldo Lopez-CharcasDaniel GroenerOscar A. Lenis-RojasDaniel L. PouliquenÓscar Rapado-GonzálezDaniel LindsayÓscar S. GallegoDaniel LipkaOsman ÖcalDaniel LorenzoOta FuchsDaniel M. KirganOthman AkhtarDaniel MartinOttavia D'OriaDaniel MenendezOttavio De CobelliDaniel NeureiterOukseub LeeDaniel NowakOxana DobrovinskayaDaniel OhlundØyvind S. BrulandDaniel OliveOzgur KutukDaniel ParamythiotisPablo E. Vivas-MejiaDaniel Patiño-GarcíaPablo EngelDaniel PecsiPablo Hernansanz-AgustínDaniel Pedregal-MalloPablo MurielDaniel PinkPablo Parra-MembrivesDaniel PotaczekPablo Ramos-GarciaDaniel PremkumarPadmaraj HegdeDaniel ReimerPádraig D’arcyDaniel RolfPäivi PeltomäkiDaniel RuessPál PergeDaniel S. K. LiuPál RibaDaniel SascaPalmieri MichelleDaniel SitarPaloma Malagon LopezDaniel SmithPaloma Ordóñez-MoránDaniel SmržPam M. Van RyDaniel TurudićPamela AchaDaniel Xin ZhangPamela JacksonDaniel ZipsPamela L. BrockDaniel ZwahlenPamela PignateliDaniela AlterioPanagiota FilippouDaniela Amidžić KlarićPanagiota MikouDaniela BassoPanagiotis BalermpasDaniela BrünnertPanagiotis F. LiaparinosDaniela CabibiPanagiotis KarakaidosDaniela CalinaPanagiotis LaïnasDaniela CoccoPanagiotis MistriotisDaniela DamianiPanagiotis N. TsikourasDaniela De ToteroPanagiotis NtellasDaniela F. Duarte CamposPanagiotis PeitsidisDaniela FurlanPanagiotis SarantisDaniela GabbiaPanagiotis T. DiamantopoulosDaniela GasparottoPanagiotis TsikourasDaniela GradinaruPanagiotis TsirigotisDaniela ImperioPanagiotis ZikosDaniela LensPanda SantoshDaniela M. SousaPandurang KolekarDaniela MarzioniPankaj AhluwaliaDaniela MennerichPankaj ChaturvediDaniela MiricescuPankaj ChaudharyDaniela RegaPankaj GaurDaniela SpanoPankaj Kumar GargDaniela TerraccianoPankita H. PandyaDaniele DerudasPanos LoukopoulosDaniele FanalePanu M. JaakkolaDaniele Filippo CondorelliPao-Chi LiaoDaniele La ForgiaPaola AllavenaDaniele MarrelliPaola AntoniniDaniele PironiPaola CassoniDaniele ScartoniPaola CostelliDaniele TaurielloPaola DamaDaniele TibulloPaola FeracoDaniele Ugo TariPaola GrimaldiDaniele VergaraPaola IzzoDanielle E. DyePaola MartinganoDaniil N. BratashovPaola MutiDanijela Đukić-ĆosićPaola ParenteDanijela Vrdoljak MozeticPaola PatrignaniDanilo De NovellisPaola SenaDanilo MatassaPaola TombesiDanny MisiakPaola TucciDanuta Gąsior-PerczakPaola ZigrinoDaochun SunPaolo AntognoniDaojun LiuPaolo BelliDaotai NiePaolo CasadioDapeng YunPaolo CastellucciDaphne de JongPaolo ContieroDaping FanPaolo Della VignaDaria A. GaykalovaPaolo DelrioDaria KurzPaolo DorettoDaria MesselodiPaolo M. ComoglioDaria SicariPaolo MalatestaDario F. GiuffridaPaolo OrsariaDario LigrestiPaolo PalmiscianoDario MirabelliPaolo PassoniDario MizrachiPaolo PellegrinoDario SiniscalcoPaolo SammartinoDario TrapaniPaolo SolidoroDarius JuškevičiusPaolo SpinnatoDarius MehreganPaolo TrucilloDariusz Wojciech MazurkiewiczPaolo VincenziDariusz WołowiecParamita DasguptaDarren J. BakerParamita M. GhoshDarren RiddyParastou TizroDarrion Luther MitchellParasvi S. PatelDarryl B. JonesParham Jabbarzadeh KaboliDaryono Hadi TjahyonoParvathaneni Naga SrinivasuDaryoush Shahbazi-GahroueiParvez KhanDat HaPascal BelleauDavid A. ScheinbergPascal LambertDavid BotequimPascal PineauDavid BrindleyPascal PujolDavid Chi-Leung LamPascale JadoulDavid CranstonPashtoon KasiDavid DanforthPasquale AuricchioDavid DoraPasquale AvellaDavid E. SpanerPasquale CianciDavid EisenstatPasquale InnominatoDavid EscorsPasquale MarrazzoDavid FeithPasquale NiscolaDavid FuksPasquale SimeoneDavid G. JohnsonPasquina MarzolaDavid GerberPatcharawalai WhongsiriDavid HardissonPatrice DesmeulesDavid HawkesPatrice ForgetDavid J. CotePatricia BerthonDavid J. HernandezPatricia SanchoDavid J. L. JoskePatricia SilveyraDavid KerrPatricio Fernández-SilvaDavid L. BartlettPatricio Gonzalez-HormazabalDavid LibichPatrick ArveuxDavid MathieuPatrick AugusteDavid MorlandPatrick HaDavid MorrisPatrick J. WorthDavid MutchPatrick M. BolandDavid N. DanforthPatrick MayrDavid OlmedaPatrick MelchiorDavid P. CookPatrick MickeDavid Parada DominguezPatrick Ming Kuen TangDavid PérolPatrick NasarreDavid PuettPatrick NevensDavid RobergePatrick PauwelsDavid RouloisPatrick WuchterDavid S. BaskinPatrik PipkornDavid S. GutteryPatrizia AgrettiDavid S. UmbaughPatrizia BalleriniDavid SermerPatrizia BertoliniDavid SibonPatrizia CancemiDavid T. MiyamotoPatrizia DorangricchiaDavid TulasnePatrizia FerroniDavid VětvičkaPatrizia LeoneDavid Wai ChanPatrizia Paterlini-BréchotDavide BernasconiPatrizia SarogniDavide BorroniPatrizia StracciaDavide CitterioPatrycja PawlikowskaDavide De CiccoPatrycja Sosnowska-SienkiewiczDavide DonnerPatryk KrzemińskiDavide GiordanoPattama WongsirisinDavide LeardiniPaul A. TownsendDavide MauriPaul B. FisherDavide MoiPaul BuchananDavide RadicePaul CiclitiraDavide TosiPaul ConstanthinDavor StamenovicPaul CottuDawei JiangPaul D. TerryDawei XuPaul E. Van SchilDawid PrzystupskiPaul Friedrich EngelhardtDawid SigorskiPaul FrohnaDawit Kidane-MulatPaul GiraudDawn QuellePaul ImbachDawn R. CochranePaul L. CrispenDa-Yong HouPaul R. AndreassenDazhong XuPaul Raymond WalkerDean Y. HuangPaul RogowskiDebanjan BhattacharyaPaul SangheraDebanjan ChakrobortyPaul T. ThevenotDebasish BasakPaul Van Der ValkDebasish Kumar DeyPaul VasosDebayan DeyPaula Alexandra PostuDebjani PalPaula BoaventuraDebmalya BarhPaula Daza-NavarroDebora Aparecida Pires de Campos ZuccariPaula DoboszDebora MacisPaula Guedes De PinhoDeborah J. VestalPaula J. HurleyDeborah LangPaula LudovicoDeborah LanniganPaula OlaizolaDeborah MalviPaul-Andrei ȘtefanDeborah MeyranPaul-François GalletDeborah Pinson HyinkPaulina CeglaDebottam SinhaPaulina FortunaDebra RitzwollerPaulina MertowskaDe-Chen LinPaulina NastałyDeclan M. SodenPaulina Niedźwiedzka-RystwejDeepak BherePaulina TokarzDeepak DhamnetiyaPaulina WlasiukDeepak KumarPaulina ZielinskaDeepak Kumar KhajuriaPauline H. GoDeepak Kumar SinghPaulo De SepulvedaDeepak ParasharPaulo MartinsDeepak VangalaPaulo MatosDeepanjan PaulPavan Kumar DhanyamrajuDeependra Kumar SinghPavel KopelDeepesh GuptaPavel LobachevskyDeepika GargPavel SoucekDeepika JaiswalPavel ŠtarhaDeepika PrasadPavlos MsaouelDeepshikha MishraPavol DubinskýDeguan LvPavol GenzorDeirdre DonnellyPaweł DerlatkaDejan StojkovićPaweł DomagałaDejan VidovicPaweł GolusińskiDejie WangPaweł KowalczykDelfien BogaertPawel PietaDelphine HudryPaweł RobakDemetrios A. ArvanitisPawel SobczukDemin LiPayam BehzadiDemitrios VyniosPedro Augusto Carlos Magno FernandesDénes LőrinczyPedro FerreiraDengchao CaoPedro Gonzalez-MenendezDenis BourgeoisPedro J. BallesterDenis CochonneauPedro Miguel RodriguesDenis MalaisePedro Ojeda-MayDenis QuerleuPedro RoldánDenis SoulieresPeeyush LalaDenise PergolizziPei-Fung WuDenise ToscaniPei-Jung LuDennis A. EichenauerPeilin ChenDennis HarrerPeiman HabibollahiDennis HwangPei-Wei ShuengDennis ShungPeiwen FeiDeo SinghPei-Yi ChuDerek HargrovePei-Yuan SuDervla KellyPekka Keski-RahkonenDésirée WünschPekka RuusuvuoriDesmond AgboadaPellegrino MazzoneDespoina KalfakakouPenelope Dawn OttewellDevanand SarkarPeng GaoDevaraj BharathiPeng GuoDevi Prasan OjhaPeng HuangDevis PascutPeng LiaoDeyali ChatterjeePeng WeiDhananjay YadavPeng XueDharmendra ChoudharyPengbo ZhouDhaval T. PatelPengze YanDheva T. SetiaputraPer I. LoftåsDhirender KattiPere PlanellasDhruvajyoti RoyPernille HolmagerDi DongPer-Olof HasselgrenDi WuPer-Olof LundgrenDiana BordinPerry MaxwellDiana CenariuPersio Dello SbarbaDiana Drago-GarcíaPetar OzretićDiana MartinsPeter AbelDiana PinhoPeter ArgentaDiana RichterPeter BrossartDiana SavuPeter C. AmbeDiana SpiegelbergPeter C. GainesDiana VellutoPeter C. HartDianbao ZhangPeter De Nully BrownDidier DecaudinPeter DevileeDidier MutterPeter DownieDidier PicardPeter F. JohnsonDidier SurdezPeter FullerDiego Dos Santos FerreiraPeter GibbsDiego F. CalvisiPeter HauDiego SoveretoPeter HauserDiesch TamaraPeter HerseyDieter HaemmerichPeter HofmannDieter HoelzerPeter HohenbergerDietger NiederwieserPeter HollanderDietmar Herndler-BrandstetterPéter HollóDietmar ZaissPeter J. SiskaDijana DetelPeter KaatschDijana DjureinovicPeter KubiszDik C. van GentPeter KuessDima SabbahPeter M. AndersonDimitar EfremovPeter MeyerDimitra GkikaPeter MúdrýDimitra Sifaki-PistollaPeter NichollsDimitri AnzelliniPeter OefnerDimitri KakabadsePeter ParsonsDimitrios A. SkoufiasPeter Ping LinDimitrios HaidopoulosPeter S. ReinachDimitrios I. AnyfantakisPeter SandwallDimitrios J. StravopodisPeter SchemmerDimitrios KorentzelosPeter SchuDimitrios KotsirisPeter SimpsonDimitrios VlachodimitropoulosPeter StålbergDimitris GorpasPeter SternDimitris StellasPeter TschannDimitris TatsisPeter UjhǎzyDimitris ZacharoulisPeter VeldhuizenDina FarrakhovaPeter W. NagleDinesh K. ThekkinkattilPeter WesterweelDinesh PradhanPetera JiříDing-Wen (Roger) ChenPeter-Paul WillemseDionysios TafiadisPetr MlejnekDionysios VorosPetr O. IlyinskiiDipayan BosePetr SedláčekDipti KanabarPetr ŠimekDirk De RuysscherPetra HeffeterDirk HempelPetra HillmannDirk NettelbeckPetra KolencDirk SchultheissPetra KoraćDirk SpitzerPetra Petranović OvčaričekDirk TheilePetra Wilder-SmithDisha MalaniPetronila PenelaDisheet ShahPetros ChristopoulosDitta UngorPetros MouratidisDivanshu ShuklaPetter BrandalDivya BhagirathPey Yee LeeDivya GopinathPhilip D. BonomiDivya MurthyPhilip D. PoorvuDiwakar Bastihalli TukaramraoPhilip H. G. ItuarteDmitrii ShekPhilip MillerDmitrijs BliznuksPhilip OwensDmitry A. StetsenkoPhilipp E. HartrampfDmitry BordinPhilipp HarterDmitry D. ZhdanovPhilipp HorvathDmitry EnikeevPhilipp J. PoxleitnerDmitry GruzdevPhilipp KronDmitry KhochenkovPhilipp LenzDmitry N. ArtemyevPhilipp M. RoessnerDoan Duy Hai TranPhilipp RennerDoerte WichmannPhilippe D'AbadieDoina AzoicaiPhilippe FortDolores D. GuestPhilippe GarrigueDolors ColomerPhilippe GarteiserDomenica LoveroPhilippe GuilpainDomenica MangieriPhilippe LefrançoisDomenica RonchettiPhilippe PourquierDomenico AlbanoPhilippe RouanetDomenico D’UgoPhilippe SaiagDomenico GalettaPhilippe SoyerDomenico GriecoPhillip T. HawkinsDomenico Maria PagliaraPia C. SundgrenDomenico SalvatorePier Adelchi RuffiniDomenico SolariPier Luigi PalmaDomenico TripodiPier Nicola SergiDominic LeiserPier Paolo PiccalugaDominik DannehlPiera RizzoloDominik GeiselPierfrancesco Francesco BassiDominik PoradowskiPierfrancesco FrancoDominik RadzkiPiergiorgio SolliDominik SaulPiergiuseppe ColomboDominik SchneiderPierluigi ToniuttoDominique ModrowskiPiero NicolaiDominique S. MichaudPiero Vincenzo LippolisDominique SchererPierpaolo AlongiDonald CameronPierre A. RobeDonald F. BeckerPierre GiglioDonat AlparPierre GuermonprezDonat KögelPierre Martin-HirschDonatella AldinucciPierre P. RogerDonatella Delle CavePierre Schembri WismayerDonatella GambiniPierre TennstedtDonatella MalangaPierre ValDonatella PontiPierre Yves BondiauDonatella RomanielloPierre-Antoine BonnetDonato ColangeloPierre-Jean SouquetDong HanPierre-Marie GirardDong Hwan KimPieter J. TanisDong Wook ShinPietro CarotenutoDong ZhangPietro Di FazioDongbo XuPietro DianaDongchul KangPietro GattiDongheon LeePietro ImpellizzeriDong-Hoon JinPietro ManiscalcoDonghoon YoonPietro MazzeoDonghui LiPietro Paolo VitielloDonghwa KimPilar CarreraDong-Joo CheonPilar Fernández MateosDonna D'agostinoPilar GiraldoDonna WallPilar Sánchez GómezDóra ParóczaiPinar Uysal OnganerDoreen LauPing ChenDoreen WilliamPing WangDoria FilipponiPing-Chih HsuDoriana FruciPing-Hui TsengDoriano FabbroPingjun ChenDoris HendigPing-Li SunDoris KalteneckerPing-Shan LaiDoris M. BenbrookPinuccia FavianaDorota BoruszewskaPin-Wen ChenDorota Magdalena Radomska-LeśniewskaPinyuan ChenDorota OrtenburgerPiotr ArtiemjewDorota RybaczekPiotr BiałasDorota Słowińska-KlenckaPiotr CzaudernaDorota SulejczakPiotr DobrowolskiDorota SzczȩsnaPiotr DzięgielDorota WrześniokPiotr GabryelDorothy R. PathakPiotr GarnuszekDorry BollPiotr GlinickiDouglas AdamoskiPiotr J. WysockiDouglas L. HillPiotr L. RutkowskiDouglas McCloskeyPiotr Mirosław MaŁkowskiDouglas Scott ErnstPiotr OstaszewskiDouglas WardPiotr PopławskiDov HershkovitzPiotr RychahouDragana D. Vasić AnićijevićPiotr ZapalaDragana KopanjaPirkko HärkönenDragana NikitovicPirouz DaftarianDragana NikolicPiyanuch KongtimDragana Vasic AnicijevicPiyush SharmaDragos MedianPlacido BruzzanitiDragos NicaPlinio CirilloDrakoulis YannoukakosPo-Chen ChuDryden BaumfalkPo-Han LinDubravko RisovićPo-Lin LiaoDuc M. LePompiliu PisoDugald SeelyPooja MandkeDuilio DivisiPooja PopliDunfa PengPoonam YadavDupoiron DenisPoorva SandleshDurai SellegounderPooyan MakvandiDušan DimićPora KimDušan KlosPorumb-Andrese ElenaDušica PahorPothula SrinivasaDüßmann HeiköPo-Yuan ChenEason HildrethPrabakaran NagarajanEberhard UhlPrabhu RamamoorthyEbrahim Mohammed SenanPradeep CingaramEddy Wai Yeung WongPradip RaychaudhuriEdel Noriega-ÁlvarezPraful R. NairEdina A. Wappler-GuzzettaPrahlad HoEdio MaldonadoPrakash GangadaranEdmond RizcallahPrakash MohanEdoardo G. GianniniPranabananda DuttaEdoardo MuratorePranshu SahgalEdoardo PeroniPrantar ChakrabartiEduardo A. VegaPrantesh JainEduardo AlgrantiPrasad S. AdusumilliEduardo CostaPrasad TrivediEduardo Garcia-RicoPrasanna VaddiEduardo ListikPrasanth Saraswati AkellaEduardo NagorePrashant ChittiboinaEdvardas DanilaPrashant JhaEdward GabrielsonPrashanth N. SuravajhalaEdward HartsoughPrashanth NageshEdward J. PavlikPrashanth RavishankarEdward KimPrasun GuhaEdward LaiPrathap SomuEdward SauterPratibha PandeyEdwige VoissetPratip K. BhattacharyaEdyta Maria UrbanskaPratyusha GhantaEdyta Marta BorkowskaPraveen BarrodiaEelco de BreePraveen KogantiEetu HeerväPraveen KumarEfrat DotanPraveen Kumar NeeliEfstratia BailiPraveen Ramakrishnan GeethakumariEftekhari AzizPravin ParajuliEfthymia PapadopoulouPravin T. P. KaumayaEgle KatkeviciutePreeti ChauhanEhsan DowlatiPreety BajwaEhsan IrajizadPrem KushwahaEhsan Nazemalhosseini MojaradPremashis KarEidi ChristensenPremila LeiphrakpamEiichi SasakiPrerna DabralEiji KiyoharaPrimrose FreestoneEiji TakahashiPriscilla BrebiEileen BrantleyPriscilla GuglielmoEileen MorganPritam SadhukhanEiman AleemPritam TayshetyeEinas YousefPrithviraj BoseEirik MalinenPriti TewariEirini RigopoulouPriyanka GhoshEirini VelliouPrzemek KrawczykEishi AshiharaPrzemysław KołodziejEjung MoonPrzemysław KozminskiEkaterina DadachovaPrzemysław M. PłonkaEkaterina PoverennayaPuja ShahroukiEkaterini ChatzakiPuneet KhandelwalEladio A. VelascoPurushoth EthirajEladio Collado-BoiraPurva V. SharmaElaine DunlopPushpa Raj JoshiElaine HarknessPyung-Hwan KimElaine Yee Lin ChungQi CaiElaine Zayas Marcelino Da SilvaQi DaiEl-Anwar Mohammad WaheedQi ShaoElda TagliabueQian DuEldad ZacksenhausQian LiuEleanor J. CheadleQian ZhangEleftheria HatzimichaelQianchuan HeEleftherios ChatzellisQianfen WanEleftherios PappasQiang DingElena Adelina TomaQiang LiElena AndreucciQi-En WangElena BertelliQijia YuElena BonoraQin FengElena CastroQing ChenElena CiagliaQing CongElena CrisaQing Richard LuElena D'AvellaQinghui WangElena Di GennaroQingming FangElena E. PohlQingshu MengElena FountzilasQingyu ZhouElena Ivanovna ShramovaQiong ZhangElena LaakmannQiongyi ZhaoElena LastraioliQixiang ShaoElena LevantiniQi-Yong Hemis AiElena MariottoQize ZhangElena MasonQu DengElena MateiQuan ZhouElena MitsiQubo ZhuElena MontiQuentin SzaboElena OngaroQun TangElena PalmaQun ZhouElena PanettieriQunxiang OngElena RaineroRabii Ameziane El HassaniElena RampazzoRachael C. NatrajanElena RussoRachael E. GuenterElena S. IzmailovaRachael HachadorianElena SaccoRachel AbbottsElena SantonicoRachel Bar-ShalomElena ShramovaRachel BayleyElena TenediniRachel EllsworthElena TomsikRachel RiechelmannElena VigliarRachel S. Van LeeuwaardeEleni MavrogonatouRachele BigiEleni RettigRachele GarellaEleni TimotheadouRadhia M’kacherEleonora DuregonRadoslava KralevaEleonora LaiRadosław ChaberEleonora PintoRadosław KempińskiEleonora PorcuRadosław MaksymEleonora SalvoliniRadoslaw PachEleonore FröhlichRadu AlbulescuElhissi AbdelbaryRadu HristuElia RanzatoRadu MineaEliano CascardiRadu Razvan ScurtuElias KochRadu TamaianElias M. EliasRaduan Ahmed FrancaElíes Fuster-GarcíaRaelene EndersbyElif Damla ArisanRafael BernardesEligijus PoškusRafael Coveñas RodríguezElin IrestormRafael DavalosElin KjelleRafael Guillén BejaranoElin Synnøve Hadler-OlsenRafael Prado-GotorElisa AgostinettoRafael Ríos-TamayoElisa BaratellaRafael RoesslerElisa CamelaRafael Salido-VallejoElisa CarrettaRafael SamaniegoElisa CassinottiRafael Sanchez-SalasElisa Dalla PozzaRafał BielasElisa GiannettaRafal KretowskiElisa GiommoniRafal ZielinskiElisa GiovannettiRaffaele AddeoElisa HerraezRaffaele BaioElisa MeldolesiRaffaele FrazziElisa PlacidiRaffaele PalmirottaElisa Stellaria GrassiRaffaele ParrozzaniElisa Ten HackenRaffaele SerraElisa ZavattaroRaffaella CatalanoElisabete BorgesRaffaella ComitatoElisabete Cristina Nunes FernandesRaffaella MassafraElisabeth BloemenaRaffaella MessinaElisabeth C. BloemenaRaffaella PetruzzelliElisabeth J. M. Nieveen Van DijkumRaghunadharao DigumartiElisabeth LippertRaghupathy VengojiElisabeth SpringerRaghuveer KavarthapuElisabetta AbruzzeseRaghuveera Kumar GoelElisabetta AldieriRaghvendra DubeyElisabetta CatalaniRaghvendra KumarElisabetta CostantiniRaheleh RoudiElisabetta GiorginiRahul BanerjeeElisabetta LalumeraRahul BhowmickElisabetta MacerolaRahul DebnathElisabetta ManualiRahul KumarElisabetta MunzoneRahul LakhotiaElisabetta PennacchioliRahul MannanElisabetta RazzuoliRahul SanawarElisabetta SetolaRaimondo Di LielloElisabetta SieniRaj KumarElisabetta TabolacciRaj Kumar MongreElisabetta Verderio EdwardsRaj YadavElise C. KohnRaja Singh PaulrajElisenda Alsina-SanchisRajalakshmi SubramaniyamElitsa Y. DimovaRajani RaiElitza P. Markova-CarRajanikanth VangipurapuEliza Glodkowska-MrowkaRajeev PandeyElizabeth A. FialkowskiRajeev SinghElizabeth Graham-GuryshRajendran J. C. BoseElizabeth NeumannRajinder DawraElizabeth SokolRajko KušecElizabeth ThomasRaju K. PillaiElizabeth YehRakesh KumarElke HattingenRaksawan DeenonpoeElla EvronRaksha BhatEllen J. BeswickRakshamani TripathiEllen KapiteijnRalf GutzmerEllen Lori WeisbergRalf HussEllen M. WesterhoutRalf KetterElliot AndrophyRalf PaschkeElmina Mammadova-BachRalph BundschuhElona JuozaitytėRalph KotheElpidio Maria GarzilloRaluca EftimieEls VerhoeyenRaluca Simona CostacheElsa IannicelliRam Kishore SinghElske Christine GootjesRama KadambElton DajtiRamanjit KaurElvio G. RussiRamesh KumarElvire Pons-TostivintRamesh NarayananElżbieta CiporaRamesh UmmanniElżbieta GrzesiukRameswari ChilamakuriElżbieta SpeinaRami Al-RohilElżbieta Wałajtys-RodeRami R. HallacEmad A. AhmedRami RhaiemEman A. HamadRamin JaberiEman A. ToraihRamireddy BommireddyEmanuel RaschiRamona ClemenEmanuela BarisioneRamona Gabriela UrsuEmanuela BerrinoRamona PalomboEmanuela GadaletaRamona Schulz-HeddergottEmanuela GrassilliRamy AbdlatyEmanuela MinnaRamya BhatiaEmanuela SpagnoloRamya Lakshmi RajendranEmanuele D. L. UrsoRamya RamaswamiEmanuele GiurisatoRana FalahatEmanuele MonteleoneRandal N. JohnstonEmanuele ScifoniRanjana MitraEmel CanbayRanjith KumavathEmelie WallinRanran KongEmer GuinanRaphael CataneEmerson Y. ChenRaphael ItzyksonEmese MezõsiRaphael KochEmiel A. M. JanssenRaphaela FritscheEmil BulatovRaquel AlvesEmilia BiałopiotrowiczRaquel CervigónEmilia PiosikRaquel MoralEmiliano DallaRaquel SitcheranEmilie Alvarez AndresRasa ZarnegarÉmilie ChalayerRashi KalraEmilie Mamessier-BirnbaumRasika SamarasingheEmilien JeannotRasoul MirzaeiEmilio BertaniRatha MahendranEmilio LeconaRatheesh Kumar MeleppatEmilio RamosRatko PrstačićEmily GordonRatna K. VadlamudiEmily KeungRatnakar TripathiEmily TheisenRaveena RamphalEmine Gulsen GunesRavi Chakra TuragaEmine Zeynep Erson-OmayRavi CholiaEmma D'IppolitoRavi P. SahuEmma LidingtonRavi RamjeesinghEmma MontellaRavindra DeshpandeEmma ZattarinRavindra KumarEmmanouil GiorgakisRavindran Caspa GokulanEmmanouil KapetanakisRavindresh ChhabraEmmanouil ZacharioudakisRavins DohareEmmanuel AntonarakisRaya LeibowitzEmmanuel DonnadieuRaymond H. KimEmmanuel GabrielRaymond P. GoodrichEmmanuel JouanneauRaymond R. MattinglyEmmanuel KatsanisRazmik MirzayansEmmanuel N. KontomanolisRăzvan George RahotăEn MengRǎzvan HainǎroşieEnas Abu-ShahRăzvan-Ionuț PopescuEndika Prieto-FernándezRebeca SanzEngy ElekhnawyRebecca LeeEnnio CadumRebecca MuirheadEnoch A. MensahRebecca P. HugheyEnric I. CanelaRedha AliEnrica Teresa TandaRedi RahmaniEnrica UrciuoliReena V. SainiEnrico AlexandreReetobrata BasuEnrico CapobiancoRegina M. DayEnrico CortesiRegina MiftakhovaEnrico De SmaeleRegina PodlasinEnrico FioriReginald HillEnrico IaccinoRegis Resende PaulinelliEnrico MartinReika Kawabata-IwakawaEnrico MoisoReiki NishimuraEnrico OpocherReinaldo GeraldoEnrico RuggeriReinhard DeppingEnrique BerjanoReinhard DummerEnrique BoldoReinhard E. FriedrichEnrique GallardoReinhard KalbEnrique M. OcioReinhard UllmannEnrique Meléndez-HeviaReinhart A. SweeneyEnriqueta L. M. OchoaReinhold FüggerEntaz BaharReinhold J. MedinaEnxiang ZhangReinhold MunkerEnzo LalliReka TothEphraim ParentRémy KinjEphrem EngidaworkRenata DuchnowskaEran Van VeldhuisenRenata Jaskula-SztulErden AtillaRenata MikolajczakErdenebileg BatbaatarRenata Pacholczak-MadejErez Bar-HaimRenata VaraljaiEric AbenojarRenata ZajączkowskaEric AdriaenssensRenate KönigEric BrouzesRenate Maria WinkelsEric C. BeyerRenato CozziÉric ChastreRenato HeidorEric ChristensonRenato Mariani-CostantiniEric Gustavo Ramírez-SalazarRenato V. La RoccaEric KalkhovenRenato ZambelloEric LechevallierRene BuchetEric LippertRene GalindoEric MartinRené HägerlingEric MasRené In ’t ZandtEric MiralliéRenee CareyEric O. AboagyeRenee GardnerEric PasmantRenee VickmanEric Paul Wehrenberg-KleeRen-In YouEric QuemeneurRenwu ZhouEric Santoni-RugiuRenzo MazzarottoEric SmithRenzo VeraEric Suero-MolinaReuben AntonyErica PranziniReuben KapurErica SalvatiReuben ToozeErich PiovanReyad DadaErick SpearsReza MortazaviErik AarntzenRhian Noble‐JonesErik H. F. M. Van Der HeijdenRiad HaddadErik J. SchoonRicard Garcia-CarbonellErik KudelaRičardas RadišauskasErik OstermanRicardo FonsecaErik SchaddeRicardo HittErika A. EksiogluRicardo MarquesErika CioneRiccardo BientinesiErika CossetRiccardo BrunaErika Gabriella KisRiccardo CasadeiErika Rendón-HuertaRiccardo Dal BelloErika StaudacherRiccardo De CarlisErin LaingRiccardo De Robertis LombardiErlinda TheRiccardo FerroErnest Ramsay CampRiccardo GiampieriErnesto GargiuloRiccardo LombardoErnst-Michael JungRiccardo MastroianniErola Pairo-CastineiraRiccardo OrusaEros Di GiorgioRiccardo RosatiErshuai ZhangRiccardo RossiErwan GuyotRiccardo SoffiettiErwin BrosensRiccardo TelliniEshan KhanRiccardo VigneriEsteban C. GabazzaRicha RaiEstela Noguera-OrtegaRichard A. AmosEsther JuliánRichard BaylissEsther K. ElliotRichard DahlEsther Louise MossRichard DrakeEsther SanfeliuRichard E. BeatsonEswar ShankarRichard E. DavisEthan L. MorganRichard E. ZigeunerEthiraj SelvarajanRichard F. RiedelEtsuko KiyokawaRichard Inho JohEtsuro BandoRichard J. HoneywellEttore Di TrapaniRichard J. PayneEugen DivjakRichard J. RebelloEugene J. LengerichRichard KulmaczEugene ShkolyarRichard LebaronEugene TulchinskyRichard NuccitelliEugenia AllegraRichard O’KennedyEugenia BroudeRichard P. ViscontiEugenia LorenziniRichard PestellEugenia MatoRichard T. WaldronEumorphia G. KonstantakouRidhima JunejaEun Jeong ParkRiggs J KlikaEunike VelleuerRika OchiEunju ChoiRiki KurokawaEun-Ju LeeRiku KlénEun-Taex OhRimas V. LukasEva Ausó MonrealRina PlattnerEva BalaziovaRinat YerushalmiEva CulakovaRintaro HashimotoEva EllebækRio BoothelloEva Fischer-FodorRio Ryohichi SugimuraEva Katharina MaselRipon SarkarEva Krieghoff-HenningRishi Man ChughEva LietoRita AssiEva NovotnáRita CasadonteEva ObermayrRita De SanctisEva RettingerRitama PaulEva Rodriguez-AznarRitesh KumarEva SlabákováRitesh SrivastavaEva SzaboRitesh ThakareEvan R. DelgadoRitin SharmaEvangelia ChavdoulaRitsuko KomakiEvangelia KoukakiRitsuko OnukiEvangelia KoutelouRittika ShamsuddinEvangelos CholongitasRitu GuptaEvangelos KoustasRitu PandeyEvangelos S. FelekourasRizwan RomeeEve-Isabelle PécheurRizwan WahabEvelien DekkerRob A. GrutersEvelina MieleRobert A. BokEveline HeijnsdijkRobert A. CarrEvelyn MonninkhofRobert A. RamirezEvertine WesselinkRobert A. SmithEvgenia KarousouRobert Arthur KratzkeEvgenii BelykhRobert B. CameronEvgeniya BurkovaRobert BoydEvgeniya KaigorodovaRobert BuscagliaEvgeny G. RybakovRobert CardnellEvgeny ZatulovskiyRóbert DócziEvie H. CarchmanRobert DrillienEwa Bielczyk-MaczyńskaRobert E. ShielEwa BurchardtRobert EckenstalerEwa CiszkowiczRobert FlisiakEwa GniazdowskaRobert GniadeckiEwa Iżycka-ŚwieszewskaRobert GoldinEwa KalinkaRobert H. MachEwa KoscielniakRobert H. ShoemakerEwa Nowakowska-ZajdelRobert HarrodEwelina GrywalskaRobert IvkovEwert BengtssonRobert J. CanterEylem Kulkoyluoglu CotulRobert K. ChinEzio LanzaRobert L. DeBernardoEzra G. BarabanRobert L. FurlerFabian BartschRobert M. KyptaFabian Flores-BorjaRobert M. VerdijkFabian LangRobert Michael HermannFabian LohausRobert MorganFabian MedvedRobert N. NishimuraFabian PlaczekRobert NewtonFabiana GregucciRobert O. DillmanFabiana NapolitanoRóbert PókaFabienne LamballeRobert PytlikFabio BaroneRobert S. OhgamiFabio BottariRobert ShambergerFabio CarboneRobert SifrerFabio CorsiRobert SitarzFabio CrucianiRobert StawskiFabio Del BenRobert Steven EsworthyFabio GasparriRobert SutcliffeFabio GuoloRobert TroticFabio MaglittoRobert W. JackmanFabio MalavasiRobert Yung Liang WangFabio MendesRoberta AngelicoFabio MinutoliRoberta AngioniFabio MonzaniRoberta AntonelliFabio R. FauczRoberta BenettiFabio StossiRoberta Elisa RossiFabio ZattoniRoberta FerrariFabio ZugniRoberta GelminiFabrice ChatonnetRoberta IbbaFabrice DarcheRoberta MaestroFabrice Lucien-MatteoniRoberta MaselliFabrizio Carnevale-SchiancaRoberta MazzucchelliFabrizio Dal PiazRoberta ModicaFabrizio FabriziRoberta NoberiniFabrizio FontanaRobert-Alain ToillonFabrizio MarcucciRobertas DamaševičiusFabrizio SignoreRoberto AltieriFada GuanRoberto AquilaniFadi MouradRoberto Augusto Ferriz-MartínezFaezeh VakhshitehRoberto BenelliFahad KhanRoberto BiancoFahim AhmadRoberto Blanco SequeirosFaisal AlzahraniRoberto BrescianiFakhredin SabaRoberto CampagnaFalk RoederRoberto CasconeFalko FendRoberto CastiglioneFalko LangeRoberto CocchiFan ZhuRoberto FabianiFanfan ZhouRoberto LemoliFang LiRoberto Martínez BeamonteFang LiuRoberto MiragliaFang XieRoberto OrecchiaFangfang QiaoRoberto PacelliFang-Ming ChenRoberto ParedesFang-Shu OuRoberto PasseraFanzani AlessandroRoberto PetriFarhad KosariRoberto PiergentiliFarnoosh Abbas-AghababazadehRoberto PiñeiroFarrukh AfaqRoberto PiroFarzad PakdelRoberto RoncaFarzad Taghizadeh-HesaryRoberto RozasFarzaneh KordbachehRoberto SalviaFatemeh KhatamiRoberto ScatenaFatemeh MakoueiRoberto SciagràFatih DurmuşoǧluRoberto ValenteFatima De PalmaRoberto WürthFausto MaffiniRoberto ZefferinoFavil SinghRobin L. JonesFederica BarbieriRobin PariharFederica BrugnoliRobin SchmitzFederica CampoloRocchetti VincenzoFederica ConteRocco TrisoliniFederica FrezzatoRochelle DsouzaFederica Genovesi-EbertRochelle TixeiraFederica GrossoRocío Aguilar QuesadaFederica GuffantiRocío BenitoFederica IavaroneRodica Maricela AnghelFederica MaccarinelliRodis D. PaparodisFederica MagagnoliRodney Edwin ShackelfordFederica MoraniRodney InfanteFederica PaoliniRodolfo Chicas-SettFederica PisaneschiRodolfo IppolitiFederica RizziRodolfo IulianoFederica TomaoRodolfo MontironiFederico BozzettiRodolfo NegriFederico BrunoRodrigo Barderas-ManchadoFederico DaviniRodrigo FerreiraFederico Lo TortoRodrigo JoverFederico LussanaRodrigo ValenzuelaFederico MastroleoRodrigue AllodjiFederico MazzaRogelio González-GonzálezFederico N AucejoRogelio González-SarmientoFederico NichettiRoger DaleFederico Pio FabrizioRoger E. ThomasFederico PozzoRoger GrandFederico QuainiRoger LengFederico RavaioliRoger OveFederico RomanoRoger P. HarrieFederico SireciRogier P VoermansFedor KryukovRohan GarjeFedor V. MoiseenkoRohan RejFei WangRohit JainFei XingRoi GazitFei YangRokas RačkauskasFeiting HsuRola JaafarFelice BurnRoland B. WalterFelice CrocettoRoland FenkFelice Van ErningRoland GeisbergerFelicity C. StarkRoland Hans StauberFelipe Andrés Cordero Da LuzRoland HoubenFelipe CalvoRoland MertenFelipe Cesar Ferrarezi BeckedorffRolf SokildeFelipe F. Quezada-DiazRolf-Dieter KortmannFelipe ParedesRomain EychenneFelix A. RuizRomain LoyonFelix BehlingRomain ParentFelix EhretRomaine C. NicholsFelix GassertRoman BlahetaFelix HüttnerRoman HrstkaFelix K. H. ChunRoman KocianFelix KorellRoman MayrFelix SternbergRoman MetzgerFeng GengRoman MezencevFeng Liu-SmithRoman NawrothFeng TangRoman SankowskiFeng ZhaoRoman SmolarczykFeng ZikaiRomano DemicheliFengchao LangRomel SomwarFeng-Cheng ChouRomeo RomagnoliFengguo XuRomina MancinelliFenghua ZhangRomina NabieeFengHuang ZhanRon FiresteinFengjuan FanRon GarnerFengru TangRon SaulnierFengzhi LiRonald A. LubetFerdinand SeithRonald AndersonFerdinand Von EggelingRonald B. BrownFerdinando AgrestaRonald B. MooreFerdinando ChiaradonnaRonald Cheong Kin ChanFerdinando Di CuntoRonald de KrijgerFerdinando NicolettiRonald G. BlasbergFerdy Van GeestRonald J. WeigelFerenc PappRonald SrokaFerenc SiposRonald TaylorFernanda De Paula EduardoRonald Van DamFernanda Ferreira LopesRonan TanguyFernandez-Rozadilla CeresRong JiaFernando AriasRong XuFernando C. BaltanásRongjin SunFernando FerreiraRongkung TsaiFernando HernanzRongqin KeFernando LópezRony SegerFernando López CamposRosa Del CampoFernando M. RohanRosa Di CrescenzoFernando MendesRosa DivellaFernando Muñoz-HernándezRosa FalconeFernando RamosRosa KlotzFernando SalvadorRosa M. MartiFernando Sánchez LasherasRosa María AyalaFernando SchmittRosa Maria MorescoFernando Torres AndónRosa Maria N. MarcussoFernando Vázquez-AlonsoRosa Maria PascaleFerran Fece De La CruzRosa Marina MelilloFerruccio BoninoRosa P. CastilloFevzi Fırat YalnızRosa PeracaulaFiamma MantovaniRosalba ParentiFidson-Juarismy VesgaRosalba TorrisiFilip DabrowskiRosalía I. Cordo RussoFilip StomaRosalinda SorrentinoFilip Y. De VosRosalyn M. AdamFilipa Fernandes MendesRosaria GangemiFilippo AlongiRosaria VarìFilippo Flavio AngileriRosario AmatoFilippo MarianoRosario CaltabianoFilippo MiglioriniRosario MaugeriFilippo MinutoloRosario SarabiaFilippo OliveriRose Marie AminiFilippo RossignoliRoseline Oluwaseun OgundokunFilippo SeverinRosellina RussoFilippo TorrisiRoser López-AlemanyFilomena CetaniRoser VelascoFinian BannonRossita M. YunusFinn Edler von EybenRouba Ali-FehmiFiona LyngRowan T. ChlebowskiFiona SchulteRowland HanFiona SimpsonRoxana Maria NemeșFiras KobeissyRoxana SirliFirdos Alam KhanRoya NavabFlaminia FerriRoza ZakanyFlávia CastroRu LiFlavio MainaRuba Al-AbdullaFlavio MilanaRuben C. PetreacaFleming GudaguntiRuben J. EckFlorence Canouï‐PoitrineRuben MedinaFlorence HuguetRuby DawsonFlorence TatinRuchi RoyFlorent PuissetRuchi ShuklaFlorentia FostiraRüdiger KlapdorFlorian BürtinRüdiger RudolfFlorian GesslerRudolf BenzFlorian HallerRuei-Nian LiFlorian HuemerRuggero CadossiFlorian K. EbnerRui AlbuquerqueFlorian KühnRui BernardesFlorian PoullenotRui DangFlorian RenosiRui GaoFlorian RoghmannRui HenriqueFlorian RosarRui M. Gil da CostaFlorian SiebzehnrublRui SuFlorian T. GassertRui VitorinoFlorian WegwitzRui YanFlorian WürschmidtRuida HouFlorieke EggermontRuifang LiuFlorin BoteaRuifeng HuFlorin George HorhatRuitu LyuFlorin TriponRuiwu LiuFlorin-Andrei TaranRukmini MukherjeeFokhrul HossainRumiana TzonevaFong-Yu ChengRunchen ZhaoForest FabienRune MatthiesenForn-Chia LinRune SmaalandFoteini KalofonouRunpu ChenFoteinos-Ioannis DimitrakopoulosRunyu HongFotis TsetsosRupert HandgretingerFranca EspositoRupesh Kumar ChikaraFranca FagioliRushendhiran KesavanFranca M. A. MelfiRuslan B. AlikhanovFranca MarinoRūta UrbanavičiūtėFrance NguyenRuta ZukauskaiteFrances L. ByrneRuth HalabanFrances Mary JohnsonRuth M. RisueñoFrancesc CanalsRuth OratzFrancesc MirallesRuth ParksFrancesca AntonelliRutika MehtaFrancesca ArrugaRuud BekkersFrancesca BenedettiRuwei LinFrancesca CitronRuxandra Diana SinescuFrancesca ColoneseRyan David MohanFrancesca CottiniRyan McGrathFrancesca De FeliceRyan SukFrancesca De TerlizziRyan WilliamsFrancesca DegrassiRyan WoodallFrancesca Di SoleRyoichi YoshimuraFrancesca FarnetaniRyoji FukushimaFrancesca GallivanoneRyoko SaitoFrancesca GiunchiRyong Nam KimFrancesca IommelliRyota MasuzakiFrancesca MaccioniRyota NiikuraFrancesca MagnoniRyota ShizuFrancesca MilanoRyota TamuraFrancesca PennatiRyuichi MizunoFrancesca RappaRyuichi WasedaFrancesca ReggianiRyuji HayashiFrancesca RicciRyungsa KimFrancesca Romana MariottiRyunosuke KogoFrancesca RossiRyusho KariyaFrancesca SalaniRyuta WatanabeFrancesca SanguedolceSaba Ahmed BeighFrancesca SensiSaba TabasumFrancesco Alessandro MistrettaSabarish NarayanasamyFrancesco AsciSabata MartinoFrancesco BaschieriSabeeta KapoorFrancesco BertoliniSabela ParadelaFrancesco BiancoSabina ŠevčíkováFrancesco BoccalatteSabina ZuracFrancesco BussuSabine ColnotFrancesco CacciatoreSabine GroeschFrancesco CeciSabine HerblotFrancesco CiarleglioSabine MaiFrancesco ClapsSabine OldenborgFrancesco CorattiSabine StrehlFrancesco D'AlòSabine Taschner-MandlFrancesco De RosaSabino RussiFrancesco FacchinettiSabira Mohammed JazirFrancesco FanfaniSabrina BonichiniFrancesco FizSabrina BossioFrancesco ForconiSabrina Daniela da SilvaFrancesco FortarezzaSabrina LisiFrancesco GiudiciSabrina StranoFrancesco LeoSabyasachi MoulikFrancesco LongoSacha J. HowellFrancesco M. PacificoSachi HoribataFrancesco MaiuriSachi Nandan MohantyFrancesco ManfrediSachio FushidaFrancesco MannelliSadahisa OgasawaraFrancesco MerollaSadeq VallianFrancesco MocciaSadia AfrinFrancesco MongelliSadiya ParveenFrancesco MorraSaed KhaliliehFrancesco OnidaSaeid GhavamiFrancesco PanzutoSafwan AlomariFrancesco Paolo CammarataSagar RayamajhiFrancesco PasqualettiSagar SohoniFrancesco PepeSahdeo PrasadFrancesco PerriSahib ZadaFrancesco PetrellaSahil InamdarFrancesco RizzettoSahiti ChukkapalliFrancesco SabbatinoSahn-Ho KimFrancesco SanvitoSai Mun LeongFrancesco SessaSai Shilpa KommarajuFrancesco SparanoSaif AfatFrancesco TarantiniSaima ImranFrancesco Tomassoni ArdoriSaima ZameerFrancesco TonelliSainitin DonakondaFrancesco TovoliSajad KhazalFrancesco TramaSajjad AfrakhtehFrancesco VoltaSakthivel MuniyanFrancina Lozada NurSalam Adli AssiFrancis JacobSally TemrazFrancis LeviSalma Ben-SalemFrancis William LuscinskasSalman Afroze Azmi MohammedFrancisca DiasSalvador IborraFrancisco Alejandro Lagunas-RangelSalvatore CaponnettoFrancisco AlvesSalvatore CocuzzaFrancisco Cammarata-ScalisiSalvatore FeoFrancisco Eduardo MartinezSalvatore FiorenzaFrancisco ExpósitoSalvatore GrisantiFrancisco J. CimasSalvatore M. CardaliFrancisco Javier Otero-EspinarSalvatore Maria CorselloFrancisco Jose Bautista SirventSalvatore Nicola BertuccioFrancisco Lázaro-DiéguezSalvatore PassarellaFrancisco Nieto-EscamezSalvatore RaffaFrancisco RealSalvatore SacconeFrancisco Sánchez‐BuenoSalvatore SorrentiFrancisco Sanchez-VegaSalvatore TafutoFrancisco TustumiSam PayabvashFranck DenatSam Yeol ChangFranck GrilletSaman Maleki VarekiFranco Alchiede SimonatoSaman WarnakulasuriyaFranco BassettoSamantha CushenFranco LocatelliSamantha KendrickFranco MarinangeliSamantha MoraisFranco MusioSamantha PayneFrançois ChevalierSamantha PollardFrançois CornelisSamar AlsafadiFrancois FaitotSamar Kamal KassimFrancois GaaschtSamarpan MajumderFrançois GuilhotSamarpana ChakrabortyFrançois JamarSameer Farouk SaitFrançois LuciaSameer H. PatelFrancois MoisanSameh AttiaFrançois MontagneSamer EzziddinFrançois VergezSami Abd ElwahabFrançois-Régis FerrandSami AkbulutFrane PaicSami Alexander SafiFrank AboubakarSamikshan DuttaFrank CackowskiSamina AlamFrank EckerdtSamir BarghoutFrank EßmannSampurna ChatterjeeFrank GriesingerSamrita DograFrank GrünwaldSamson Mathews SamuelFrank J. JenkinsSamsul Ariffin Abdul KarimFrank KruytSamuel Aguiar JrFrank QianSamuel BuxtonFrank SiebenhaarSamuel E. AdunyahFrank SzulzewskySamuel MeignanFrank Vinholt SchiødtSamuel O. AntwiFrank WeberSamuel Peña-LlopisFranscisco X. RealSamuel S. StreeterFranz RödelSamuel SamnickFranz TrautingerSamuel T. OrangeFranz ZehentmayrSamuel William David MerrielFranziska SiegenthalerSamuele GhezzoFranz-Josef SchingaleSamuele MassarutFrederic CourbonSamuele VaccariFrédéric LézotSamuil UmanskyFrédéric MarchalSamy JambonFrédéric Rieux-LaucatSanae MidorikawaFrederic TriponezSanaullah SajibFrederick H. SilverSandeep K. MishraFrederik A. StuebsSandeep RanaFrederik DammSandi HayesFrederik De SmetSandip S. ShindeFrederik HoogwaterSandipan DattaFrederike DijkSandra A. DrigoFrédérique FrouinSandra BrosdaFrédérique Megnin-ChanetSandra CasimiroFrederique Ruf-ZamojskiSandra HöglerFredric D. GordonSandra LejnieceFredrik LehmannSandra SchmitzFredrik SchjesvoldSandra SchönigerFredrik WärnbergSandra SigalaFriedrich GraesserSandra SkujaFriedrich HerbstSandra V. FernandezFu LuoSandra WinningFuiboon KaiSandra ZampieriFulvia VascottoSandrine AspeslaghFulvio BasoloSandrine DabernatFulvio ChiacchieraSandrine Valsesia-WittmannFulvio ZaccagnaSandro Jose Ribeiro BonattoFulvio ZulloSandro KriegFuminori SakuraiSandro PasqualiFumitaka KogaSang Moo LimFumitaka OhnoSang Seong KimFung Fuh WongSang Wun KimFurlan AlessandroSanghee KangFuyang LiSang-Ho JeongFuyi WangSang-Hyun MinFuyuhiko MotoiSanguine ByunFu-Zong WuSanja DabelićG. Eric BlairSanja Lovric KojundzicGábor HalmosSanjay GuptaGábor LipositsSanjay MaraboyinaGábor LotzSanjay MishraGabor MikalaSanjay R. KharcheGabriel BenavidezSanjeev ShuklaGabriel GlockzinSanjit MukherjeeGabriel LiberaleSankar JagadeeshanGabriel SamascaSankar MaityGabriel WajnbergSankar SwaminathanGabriela AniteiSanne Lysbet De HaasGabriela Chavez-CalvilloSanta Di CataldoGabriela SchneiderSantanu BhattacharyaGabriela Torres-MejíaSantanu MajiGabriele CapursoSanthanasabapathy RajasekaranGabriele CarulloSanthosh ArulGabriele GrassiSanthosh Kumar PasupuletiGabriele PiccoSantiago CepedaGabriele ReinartzSantiago DomingoGabriele RomanoSantiago Gómez-RuizGabriele SaretzkiSantiago HaaseGabriele SpoletiniSantiago Ropero SalinasGabriele TodiscoSantos ManesGabriella CaminatiSantosh KarnewarGabriella Campadelli-FiumeSantosh Kumar SinghGabriella CsíkSantosh PeddiGabriella D’OraziSara CaratelliGabriella MacchiaSara FranziGabriella NicoliniSara Gutierrez-EnriquezGabriella PellegritiSara H. KassaniGabrielle Van RamshorstSara Huerta-YépezGaetano FinocchiaroSara JamesGaetano GalloSara LacerdaGaetano MarvertiSara MassironiGaetano PiccoloSara NavaGaetano RiemmaSara OrecchiaGagan D. GuptaSara PeriGaganpreet KaurSara PilottoGaia BarisioneSara R. Martins-NevesGaia PalminiSara RicardoGaia SpadarellaSara S. ReisGail A. BishopSara SergioGail L. MattersSara TrabanelliGalatea KallergiSara ZapicoGalaxia M. RodriguezSarada Preeta KalainayakanGanesh GunasekaranSarah A. WallGanesh RaoSarah DerksGang ChenSarah F. AdamsGang HuangSarah GehlertGang WeiSarah HolsteinGang WuSarah K. SchröderGang ZhouSarah KamelGangadhara SareddySarah L. SawyerGanji P. NagarajuSarah MartaindaleGaoussou TouréSarah Noémie DumontGareth EvansSarah PriceGareth MorganSarah SheltonGarima KaushikSarah TettamantiGarima SharmaSarah WalkerGarret A. FitzGeraldSarah WatsonGary D. SteinbergSarah WoodfieldGary GellermanSarang TarteyGary H. LymanSarantis GagosGary L. FrancisSaratchandra KhumukchamGary MartinezSarel HalachmiGary S. LeiserowitzSarfaraz IqbalGąsowska-Bajger BeataSari LiebermanGasparini PatriziaSarika GuptaGašper KlančarSarit AppelGatikrushna PanigrahiSarka NemeckovaGaurav JoshiSarv PriyaGaurav PandeySassan HafiziGaurav SharmaSatheesh Kumar SengodanGautam SondarvaSatheesh SengodanGaya Punnia-MoorthySathish K. R. PadiGeert RaesSathyavathi ChallaSivaKanakaGeeta UpadhyaySatoru KaseGelan AyanaSatoru MunakataGema Jiménez-GonzálezSatoru OsukaGemma Ramirez-VillarSatoshi MatsukumaGemma SkaczkowskiSatoshi NagayamaGeneviève C. DigbySatoshi NakasuGenevieve DallSatoshi NaraGennaro CormioSatoshi TakahashiGennaro DanieleSatoshi WashinoGeoff ChongSatoshi WatanabeGeoffrey J. ClarkSatya DasGeoffrey Yuet Mun WongSatya KotaGeorg FlügenSatya Ranjan MisraGeorg MellitzerSatyabrata AichGeorg SchlachtenbergerSatyendra C. TripathiGeorge A. BrooksSaulius CicènasGeorge AnsstasSaum GhodoussipourGeorge B. SmallfieldSaurabh AgarwalGeorge BlanckSaurav Kumar JhaGeorge Calin DindeleganSaveg YadavGeorge E. KarpetasSaverio AlbertiGeorge H. Au-YeungSaverio CandidoGeorge I. SaltiSavio BarretoGeorge JourSavio Domenico PandolfoGeorge KulikSavvas LampridisGeorge Mihai NitulescuSavvas PetanidisGeorge P. BaylissSawako SuzukiGeorge P. ParaskevasSawako Uchida-KobayashiGeorge PapaxoinisSawsan A. ZaitoneGeorge PotamiasSawsan MohammedGeorge RakovichSayan ChakrabortyGeorge SharbeenSayantani Sarkar BhattacharyaGeorge SharonovSayed K. GodaGeorge ShenoudaSayem MiahGeorge TherapondosSazan RasulGeorge TzanakakisScarfì FedericaGeorge ValasoulisSchelto KruijffGeorge ZanazziScott FisherGeorges LeclercqScott KehlerGeorgia GomatouScott M. SchuetzeGeorgia HalkettScott N. PenfoldGeorgia KarpathiouScott OkunoGeorgia RagiaScott Richard FloydGeorgia Zhuo ChenScott S. VerbridgeGeorgina Correia-da-SilvaSeamus O’ReillyGeorgios Antonios MargonisSean Chun Chang ChenGeorgios D. LianosSean HynesGeorgios D. MichailSebastià SabaterGeorgios GaitanisSebastian AsafteiGeorgios GermanidisSebastian DahleGeorgios K. GlantzounisSebastian DraackGeorgios LaliotisSebastian GassenmaierGeorgios MalietzisSebastian MakuchGeorgios S. MarkopoulosSebastian MedinaGeorgios VrakasSebastian O. WendelGeorgy MikhaylovSebastian ObrzutGerald McGwinSebastian PonsGerald V. DoyleSebastian RegneryGeraldine GueronSebastian SchölchGeraldo Célio BrandãoSebastian SchollGerard CumminsSebastian SzmitGerard JansenSebastian T. BalbachGerardo Blanco-FernandezSebastián Valverde-MartínezGerardo CarusoSebastian WendelGerardo FerbeyreSebastian ZahnreichGerardo MordojovichSebastiano RontauroliGerardo MusuracaSébastien PenninckxGerasimos P. SykiotisSébastien RogerGerassimos E. VoutsinasSébastien WälchliGerd FastnerSeckin AkgulGerda EggerSeddik HammadGergely KeszlerSégolène HescotGergely RónaSehime TemelGergely VargaSeigo NakamuraGerhard DahlSeigo OhbaGerhard GrobnerSeiichi IshidaGerit TheilSeiichi KitagawaGerlig WidmannSeiichi MatsumotoGermaine EscamesSeiichiro FukuharaGertjan Jan L. KaspersSeiichiro IshiharaGeta CârâcSeiichiro MitaniGhadah Nazar Al-jussaniSekyung OhGi Hyeon SeoSelcuk KeskinGiacomo BasadonnaSema K. AydedeGiacomo CanesinSemih Can AkıncılarGiacomo CorradoSengupta SagarGiacomo Maria PirolaSenthilkumar KalimuthuGiacomo MiserocchiSeo-hee ChoiGiacomo ZanusSeokchan EunGiada Maria VecchioSeokhyun YoonGiada PaulettoSeong Beom ChoGiamberto CasiniSeongyong ParkGiammaria FiorentiniSerafín M. MoralesGian Carlo MattiucciSerena BianchiGian Franco ZannoniSerena BoninGian Luca Rampioni VinciguerraSerena LucottiGian Marco ConteSerena MeravigliaGiancarlo PecorariSerena PellegattaGiandomenico MaggioreSerena PsoroulasGianluca BaldanziSerenella Eppenberger-CastoriGianluca CiardiSerenella PupaGianluca De MasiSergei E. TitovGianluca FeriniSergei KruglikGianluca GaidanoSergei NechaevGianluca IngrossoSergei TverdokhlebovGianluca MasiSergej PirkmajerGianluca SalaSergey A. RodinGianluca SpenaSergey BrezginGianluca TenoreSergey G. Afanas’evGianluca TrevisiSergey KazakovGianluigi CalifanoSergey KolomeichukGianmarco PallaviciniSergey KulemzinGianna MariottiSérgia VelhoGiannantonio SpenaSergio CortelazzoGianpaolo PapaccioSérgio DiasGianpaolo VaccarellaSergio García-GarcíaGianpaolo VidiliSergio Granados-PrincipalGianpietro SemenzatoSergio MalutaGiedre KrenciuteSergio MarchiniGiedre SmailyteSergio OcchipintiGiedrius BarauskasSergio Piñeiro HermidaGilbert LimSergio SalernoGilles GadeaSergiusz NawrockiGilles HouvenaeghelSeri JeongGilles RademakerSeth FrietzeGinés HernándezSeth PollackGin-Shin ChenSeung Eun LeeGiorgia CarecciaSeung-bae YoonGiorgia GarganeseSeung-Gu YeoGiorgia MangiliSeungil RoGiorgio BoganiSeungyeul YooGiorgio GinesuSevastita IordacheGiorgio LofreseSeverin DaumGiorgio MalpeliSevilay Tümkaya YilmazGiorgio ManginoSevin TurcanGiorgio RispoliSevinci PopGiorgio TregliaSeyed Javad MoghaddamGiou-Teng YiangSeyed Peyman ShariatpanahiGiovambattista PaniShadma W. AbdulwahabGiovana Tardin TorrezanShafat AliGiovanna CalabreseShafiq U. AhmedGiovanna LiguoriShahab AlizadehGiovanna MantiniShahab UddinGiovanna MasalaShahana MahajanGiovanna RigilloShaheen KhanGiovanna RussoShahneen K. SandhuGiovanna TranfoShahriar FaghaniGiovannella PalmieriShahrokh GhobadlooGiovanni AbatangeloShahzaib AhamadGiovanni BarillariShailendra GautamGiovanni BarosiShailendra Pratap SinghGiovanni Battista Levi SandriShaker El-SappaghGiovanni ButturiniShakir KhanGiovanni CannarozzoShakur MohibiGiovanni CazzanigaShama P. KabekkoduGiovanni ChiappettaShamir CassimGiovanni CochettiShamus CarrGiovanni Codacci-PisanelliShan AliGiovanni ColomboShane AustinGiovanni CorsoShang SuGiovanni CrisafulliShang-Ju WuGiovanni CristalliShankar Jaikishan EvaniGiovanni CugliariShanmuga MahalingamGiovanni D. TebalaShanmugarajan KrishnanGiovanni DurandoShannon J. OdelbergGiovanni GermanoShanshan TanGiovanni Guglielmo LaraccaShanshan WangGiovanni Luca CeresoliShantanu GuptaGiovanni MarconiShao LiGiovanni Maria ComacchioShaobo RuanGiovanni Maria IannantuonoShao-Hsuan KaoGiovanni N. RovielloSharad AwasthiGiovanni NassaSharanjot SainiGiovanni NatileSharda KumariGiovanni PaolinoSharda P. SinghGiovanni RossiSharmila FagooneeGiovanni RostiSharon A. O'TooleGiovanni SalzanoSharon E. BarrettGiovanni ScarzelloSharon HouGiovanni TossettaShasha YinGiovanni TuccariShashank S. GhantojiGiovanni VitaleShashi Shekhar SinghGiovanni ZoccaliShatadal GhoshGirdhari LalShatavisha DasguptaGirish BathlaShaun A. NguyenGirish ShuklaShaun BrothersGirolamo RanieriShawn C. ChafeGirum GetachewShayan ShamsGisberto EvangelistiShazia BanoGiseli KlassenShazia JamshedGiselle CerchiaroShazid Md. SharkerGiselle Y. LópezSheba R. DavidGiuditta ChiloiroShebli AtrashGiuditta MannelliSheeba SujitGiulia BerzeroSheeja Perumbil PathroseGiulia BorgheseSheetal ParidaGiulia CantiniSheida MoghadamradGiulia CarosiSheila SpadaGiulia FontanaShekhar SahaGiulia M. StellaShelley HooksGiulia MansuttiShelly LoewensteinGiulia PozziSheng Dean LuoGiulia SantinonSheng FuGiulia SantoSheng HoGiuliano ZabuchiSheng ZhuGiulio C. SpagnoliShengchieh ChanGiulio DinardoShenglin MaGiulio FerreroShenglin MeiGiulio FrancoliniShengmei ZhouGiulio IlluminatiSheng-Mou HsiaoGiulio MairaSheng-Po HaoGiulio PilusoSheng-Xing MaGiulio RossiSheren YounesGiulio TessarinSherif El‐MasryGiulio VaraSherine F. ElsawaGiuseppe AcriSherry S. CollawnGiuseppe Alberto PalumboSherry X. YangGiuseppe AmorusoShiang-Fu HuangGiuseppe BadalamentiShiao WooGiuseppe BardiShibo YuGiuseppe BroggiShidan WangGiuseppe Carlo IorioShigeki SugawaraGiuseppe CatanutoShigeki YamaguchGiuseppe CinalliShigeo ShimoseGiuseppe CirilloShigeru FujisakiGiuseppe ColucciShigeru MatsumuraGiuseppe DeleddaShigetaka ShimodairaGiuseppe Di MartinoShigetaka YoshinagaGiuseppe Emmanuele UmanaShigeyoshi FujiwaraGiuseppe FanciulliShigeyoshi SaitoGiuseppe FanettiShigeyuki TakamatsuGiuseppe G. LoscoccoShihao ShenGiuseppe GiacconeShi-He LiuGiuseppe GiudiceShih-Hsien HsuGiuseppe IatíShih-Hsuan ChanGiuseppe LucarelliShihlung ChenGiuseppe MigliarettiShih-Lung ChengGiuseppe MorgiaShihori TanabeGiuseppe NocentiniShih-Yen HsuGiuseppe PernaShijie HeGiuseppe RivaShikhar MehrotraGiuseppe RobertoShilpa BishtGiuseppe RossiShin HamadaGiuseppe SammarcoShin NishioGiuseppe SanguinetiShin-Cheh ChenGiuseppe SarpietroShindu ThomasGiuseppe SchepisiShingen NakamuraGiuseppe SimoneShingo HatakeyamaGiuseppe SprianoShingo TsujinakaGiuseppe StragliottoShin-Hyung ParkGiuseppe TisoneShinichi KinamiGiuseppe ToniniShin-Ichi OsadaGiuseppe VerlatoShinichi SuzukiGiuseppe ZardoShin-ichiro FujiiGiuseppe ZimmittiShining LooGiuseppina CamioloShinji ItohGiuseppina De SimoneShinji KawabataGiusy TornilloShinji TakamatsuGlauco Akelinghton Freire VitielloShinji TsukamotoGlauco ChisciShin-Ming HuangGlenn CruseShinnichi OkazumiGleyson Kleber Do Amaral-SilvaShintaro SukegawaGloria Álvarez-SolaShinya MaekawaGloria Gutiérrez-VenegasShinya MatsuzakiGloria ManzottiShipra AgarwalGloria PascualShirisha JonnalagaddaGloria RavegniniShirley D’SaGniewko WięckiewiczShiro UrayamaGobi Saravanan KaliarajShiu-Feng HuangGodbout RoselineShiv Prakash VermaGodelieve A. M. TytgatShiv Shankar VermaGokce TorunerShiv SrivastavaGokhan BahceciogluShiva KantGoki SudaShivanand M. PudakalakattiGokul DasShivanand PudakalakattiGolrokh MirzaeiShivani SoniGon Sup KimShivankar AgrawalGongqin SunShiyi YinGonzalo VarelaShi-Yong SunGoo LeeShizhao LiGopichand PendurtiShmuel AvitalGoran BrajuškovićShobana NavaneethabalakrishnanGoran GajskiShogo EhataGoran VujićShogo ShinoharaGorana GašljevićShohei KawaguchiGordana KocicShoichi FujiiGordon WinterShoji OuraGovind SrimathveeravalliShorook Na’araGraça SoveralShota NakamuraGrace S. LeeShota YamamotoGraciela Caire-JuveraShotaro NakajimaGraciela Martínez-PalliShowkat DarGracjan MichlewskiShravan GiradaGrady NelsonShreyas GaikwadGraeme KoelwynShreyas S. RaoGraham LeggattShrikant PradhanGraham R. WallaceShrikanth GadadGrazia AmbrosiniShriram VenkatesanGrazia FazioShruthi SriramkumarGrazia MennaShuai GaoGrazia PalombaShuai MaoGraziana GalloShuai WangGraziella CurtaleShu-Chi WangGraziella Di GreziaShu-Chun ChangGrazyna Kaminska – WinciorekShu-Fen PengGreg ZapotocznyShu-Fu LinGregg B. FieldsShuhei YoshidaGregor NorcicShuichi MitsunagaGregor SersaShuji MomoseGregor StavrouShuji OzakiGregorio ScerrinoShu-Ling TzengGregory A. LizeeShumei SongGregory KaltsasShun YamamotoGregory M. PalmerShuncong WangGregory N. GanShun-Fa YangGregory S. YochumShungang ZhangGregory W. AlbertShunichi TakedaGregory YochumShunqing LiangGreta AlìShuntaro ObiGreta CapraraShunya OhmuraGreta VarchiShu-Pin HuangGretel Mendoza-AlmanzaShusen ZhengGrzegorz BartoszShusuke TodenGrzegorz SzewczykShuta OharaGuadalupe Rojas-SanchezShuvashis DeyGuanbin SongShuvasree SenguptaGuangda PengShuyuan YehGuangfu LiShweta ArasGuangju ZhangShweta KukrejaGuangtao YuShweta Pankajkumar LavaniaGuanhui WuShweta TripathiGuankui WangShwetabh SinhaGuannan WangShyam Kumar GudeyGuanshu LiuShyam SharanGuanying XuShyng-Shiou F. YuanGudrun E. KoehlSibylle KranzGudrun KoehlSiddharth S. MatikondaGudrun KreyeSigne FrieslandGuendalina LucariniSigrid A. LanghansGuglielmo ManticaSigrun ThorsteinsdottirGuido Alberto Massimo TiberioSigurd F. LaxGuido BispingSijo MathewGuido Di DalmaziSik Kwan ChanGuilherme MacedoSilke HaerteisGuillaume Anthony OdriSilvana CanevariGuillaume FichesSilvana CiardoGuillaume GauchotteSilvana Del VecchioGuillermo De VelascoSilvano FerriniGuillermo Til-PerezSilvia BozzarelliGuiquan ZhuSilvia CataneseGuirong DingSilvia CatuognoGuishan XiaoSilvia ChichiarelliGuiyang HaoSilvia De SanjoséGulzhanat AimagambetovaSilvia DeaglioGun Oh ChongSilvia Di AgostinoGunnar KristensenSilvia FilippiGunnar WichmannSilvia FrancisciGünter KlöppelSilvia GalardiGünther A. RezniczekSilvia GalbiatiGüntülü AkSilvia GonellaGuodong FuSilvia GramolelliGuodong ZhangSilvia LiuGuo-Liang LuSilvia MarracciGuoqiao ZhengSilvia MarsoniGuowei KimSilvia MeziGuoying WangSilvia MolinelliGurdeep SinghSilvia NatoliGurpreet Kaur AroraSilvia PeppicelliGustavo Alberto ChiabrandoSilvia PesceGustavo GarciaSilvia RaveraGustavo PimentelSilvia Regina RogattoGustavo R. SarriaSilvia RiondinoGustavo TapiaSílvia Rocha-RodriguesGuy FroyenSilvia TyčiakováGuy G. PoirierSilvia VanniGuy LeclercqSilvio MonfardiniGwenaël PagéSimin JamalyGwendal LazennecSimin SharifiGwendolyn M. CramerSiming GongGwo-Tarng SheuSimon C. BakerGyeong Eun MinSimon FoxGyöngy MiklósSimon GarinetGyórgy ParaghSimon HaywardH. Denis AlexanderSimon HorslenH. GroebenSimon John Christoph SoerensenHabyeong KangSimon LangdonHadi A. Goubran MessihaSimon LawHadi HashemzadehSimon S. LoHadi MessihaSimon SauleHadith RastadSimon SpohnHae-Seong NamSimon VölklHafiz AhmedSimona CameroHaggi MazehSimona CimaHai HuSimona CitroHai WangSimona Delle MonacheHaibo YangSimona FerraroHaifa Kathrin Al-AliSimona GiuratrabocchettaHaifeng WangSimona GurzuHaifeng YangSimona MartinottiHailing ZongSimona MoldovanuHaining YangSimona PagliucaHaiqiu HuangSimona RaimoHaitham A. ElmarakebySimona-Roxana GeorgescuHaixun GuoSimone AnfossiHaiyan ChuSimone De LeoHaiyan XuSimone E. MuenstHajime IsomotoSimone KrebsHajime YanoSimone MorraHajra ZafarSimone MorselliHak ChangSimone PatergnaniHakim MahammediSimone ScarcellaHakim Ouled-HaddouSimone SerafiniHalilibrahim CiftciSimone TamburriHaluk DamgaciogluSimran VenkatramanHaluk TopalogluSinan DemircioğluHamed Mohebbi-KalkhoranSinghal RashiHamid AbdollahiSiobhán V. GlaveyHamid BandSiqi GuoHamid MorjaniSiqi WuHamidou KeitaSirish DharmapuriHamidreza ArdalaniSiro SimizuHan HanSitaram GayatriHan Naung TunSithembile MabilaHan ZhangSiu Wai ChoiHana StarobovaSiva Karthik VaranasiHana ŠtudentováSiva Lahiri Kanth NanduriHanbing SongSivakumar Vadivel GnanasundramHandoo RheeSivaraman NatarajanHanefí ÖzbekSiyi GuHang Fai KwokSiyong KimHani J. MarcusSiyuan SuHanley N. AbramsonSj ShenHannah StevensSjoerd T. SchettersHann-Chorng KuoSjors G. J. G. In’t VeldHanne LieSladjana ZagoracHanno M. WitteSmita MisraHans BrunnströmSmrithi PadmakumarHans ChristiansenSmriti PandaHans F. FuchsSmriti SinghHans Friedrich FuchsSneha SaxenaHans G. DrexlerSneha ShahHans H. HendriksSneha VivekanandhanHans HertzSnjezana JanjetovicHans KlingemannSo Jin ShinHans KnechtSo KwonHans LennernäsSobhi F. LamlomHans MorreauSofia ChatziioannouHans Peter LangSofia EdinHans-Christian KolbergSofia SousaHan-Sin JeongSohei SatoiHans-Joachim SchulzeSoheil Abbaspour-RavasjaniHans-Jonas MeyerSohinee BhattacharyyaHans-Jürgen BiersackSohini ChakrabortyHans-Michael HauSohyun HwangHans-Ulrich BenderSoichiro MurataHanwen WangSoichiro YoshidaHany I. SakrSokcheon PakHao ChenSokol TrunguHao NieSolange LandrevilleHao WangSoldano FerroneHao ZhangSolène M. EvrardHao ZhouSolli PiergiorgioHao-Lun LuoSolmaz Maleki DizajHaoqiang YingSolomon AfelikHaoSu ZhangSomaira NowsheenHarald PlattaSomchai ChutipongtanateHaralds PlaudisSomiranjan GhoshHardeep Singh TuliSomnath PandeyHardev PandhaSoňa ČiernikováHarikumar RajaguruSoňa LegartováHarinder GillSonal SukreetHarit PandaSonali P. BarweHarland NiklasSonam KumariHarleen ChelaSong YaoHarm Van MelickSongli ZhuHarm WestdorpSonia A. MeloHarold LauSonia FantoneHarold Trent SpencerSonia M. OliveiraHarold Y. LauSonia MissiroliHarpreet SinghSonia NathHarriët Jager-WittenaarSonia ValletHarrys Kishore Charles JacobSonja DragojevicHarsh Vardhan JainSonja Eriksson SteigenHarshani WijerathneSonpavde GuruHarshraj LeuvaSoomi KimHartmut GoldschmidtSoo-young KimHaruki KoikeSophia M. SchmitzHaruko HayasakaSophia W. M. BruggemanHaruko NumajiriSophie ChiavassaHaruko TashiroSophie ClementHaruto NishidaSophie DeguelteHasan MujtabaSophie DerchainHashem O. AlsaabSophie LelorainHassan A. KhalilSophie PilleronHassan RanjbarSophie VasseurHassan RasouliSoranobu NinomiyaHasserjian Robert PaulSoraya TalebHathal HaddadSøren MøllerHayato HikitaSøren Paludan SheikhHaydeé Rosas-VargasSotirios P. FortisHayel DeraniSotirios ZarogiannisHe LiSougata SahaHe ZhuSoumen SahaHeath Devin SkinnerSoumyashree GangopadhyayHeather HimburgSounak SahuHeather StuartSourav PanjaHeba SahyonSouvick RoyHedwig DeubzerSowjanya ThatikondaHedwig Sutterlüty-FallSowmiya A. MoorthieHee Jun ChoSpanier GerritHee Jung KimSpartaco SantiHee Man KimSpencer BehrHee Sue ParkSpiros VlahopoulosHee-Sung SongSpomenka ManojlovićHeeyoung LeeSpyridon GennatasHei KimSpyridon TsiourisHeidi FieglSpyros ManolopoulosHeidi KaljunenSrdjan NikolovskiHeidi SowterSreenivasulu ChintalaHeidrun BoztugSreeparna ChakrabortyHeike NiessnerSreyashree BoseHeike SchmidtSridevi ChallaHeiko BecherSridhar ArjunanHein Van PoppelSridhar MuthusamiHeiner SchäferSridhar RadhakrishnanHeinz FreislingSridurga MithraprabhuHelen E. RemottiSrikanth RapoleHelen Jane BoyleSriram GubbiHelen KaliraiSriram SundaravelHelena FerreiraSriram YennurajalingamHelga BergholtzSrividya SwaminathanHelge GadSriyani PadmalathaHella Anna BolckSruthi RavindranathanHelmut BeltraminelliStaci L. SudengaHelmut FriessStan B. SidhuHeloisa Sobreiro Selistre De AraujoStanislav MakhanovHéloïse BourienStanislav VasilyevHemant KocherStanislaw DejaHemanth NoothalapatiStankevičius VaidotasHemendra GhimireStanley LipkowitzHendrik UngefrorenStavroula BaritakiHenrik FalconerStavroula GiannouliHenrique Borges Da SilvaStefa WiemannHenry FechnerStefan AmbsHenry M. PrinceStefan BartzschHenry Miles PrinceStefan BuettnerHerbert LevineStefan BurdachHerbert PichlerStefan GrauHerbert Ryan MariniStefan HarsanyiHerbert YuStefan HohausHermann B. FrieboesStefan J. JanikHernando Lopez-BertoniStefan JanssenHeshan Sam ZhouStefan KnopHeshui WuStefan KrauseHibiki UdagawaStefan KurtenbachHideaki KawabataStefan P. HaiderHideaki NakajimaȘtefan PătrașcuHideaki OgiwaraStefan PuschHideaki ShimadaStefán SigurðssonHidehiko KikuchiStefan SimmHidehiko TakigawaStefan WelterHidehisa TakahashiStefan WinterHidehito HorinouchiStefan WölflHideki TokunagaStefania CanèHideki WanibuchiStefania CanovaHideki YokooStefania CrucittaHideo BabaStefania CuzzubboHideo SuzukiStefania GalimbertiHideo TsukadaStefania GobessiHidetaka OhnukiStefania MazziniHidetaka YamamotoStefania MitolaHidetatsu OutaniStefania TommasiHidewaki NakagawaStefania VolpeHideyuki J. MajimaStefanie AustHideyuki TakeuchiStefanie KleinHideyuki TerazonoStefanie LeschHilary A. KennyStefanie PlageHimadri BiswasStefanie R. BaileyHimangshu SonowalStefano BarbiHimanshi BhatiaStefano BrillantiHimanshu SoniStefano CacciatoreHinne A. RakhorstStefano CampanerHira Lal GoelStefano DiciottiHiroaki IwasaStefano FantiHiroaki KonishiStefano FerrettiHiroaki MiyoshiStefano FumagalliHiroaki TaniguchiStefano GarofaloHiroaki TobaStefano GentileschiHiroaki WakimotoStefano GranieriHirofumi JonoStefano IndraccoloHirofumi NakatomiStefano LafranceschinaHirokazu TakamiStefano LuzzagoHiroki SatoStefano MagnoHiroki TanabeStefano MarroneHiroki TanakaStefano MoalliHiroki YokotaStefano MolicaHiroko YamashitaStefano Piero Bernardo CioffiHirokuni TakanoStefano RauseiHiromi KurokawaStefano RealdonHiromichi ItoStefano RestainoHironao OkuboStefano RomagnoliHirono IriuchishimaStefano SettimiHiroshi DoiStefano SeveriHiroshi FujitaStefano SignoroniHiroshi HandaStefano SpieziaHiroshi KagamuStefano TaboniHiroshi KatoStefano TomassiHiroshi NishiokaStefano UgelHiroshi UreshinoStefano UrsinoHiroshi WakabayashiStefano VaggeHiroshi YoshidaSteffen HauptmannHirotaka MatsuiSteffen HeegaardHiroto OhguchiSteffen JunkerHirotoshi KikuchiSteffen OrmannsHiroya KuroyanagiSteffen SpoerlHiroyasu IwasakiSteffen ThomasHiroyoshi KunimotoSteffen WagnerHiroyoshi Y. TanakaStefka TanevaHiroyuki DaikoStephan AltmayerHiroyuki GodaStephan BernhartHiroyuki KatayamaStephan J. ReshkinHiroyuki NojimaStephan Joel ReshkinHiroyuki SuzukiStephan KerstingHiroyuki TakahashiStephan NilandHiroyuki TakamatsuStephan RogallaHiroyuki TakedaStephan ScheideggerHiroyuki TakeiStephane BolducHiroyuki TsuchieStéphane ChabaudHiroyuki TsuchiyaStephane ChauvieHiroyuki TsudaStephane R. GrossHiroyuki UetakeStephane ServaisHisako FujiharaStephane TerryHisashi IzasaStéphane VignesHisashi KosakaStephanei MayHisham BahmadStephanie B. DixonHisham FansaStephanie BolleHitendra Singh SolankiStephanie J. YaungHitler LouisStephanie KingHitoshi MaemotoStéphanie Leroy-LhezHitoshi TsudaStephanie MaHiu Chuen LokStephanie MangesiusHo TsoiStephanie McArdleHo-Hyung WooStéphanie Nguyen-QuocHoi Leong Xavier WongStephanie RoesslerHojin MoonStéphanie SimonciniHo-Jong ChunStephanie SivellHolger A. LindnerStephanie StanfordHolger AunerStephanie YoonHolger Hans Herman ErbStephen A. BernardHolger RumpoldStephen AhnHong DuanStephen FalkHong FangStephen Fernandes RodriguesHong Kwan KimStephen J. RichardsHong TruongStephen J. TerryHong WanStephen J. WilliamsHong ZanStephen M. ModellHong ZhaoStephen OpatHongbeom KimStephen P. FinkHongbin LuStephen StrumHongda ChenStephen T. SonisHong-Duk YounStephen YipHongjun JinStergios BoussiosHonglei ChenStergios KatsiougiannisHongliang HeSteve ShnyderHongran YinSteven BaumruckerHong-Shiue ChouSteven CenHong-Tao LiSteven CliffordHongyue ZhouSteven EsworthyHongzhang HeSteven F. GameiroHon-Yi ShiSteven FieringHop Tran CaoSteven FrischHosam O. ElansarySteven KnapperHo-Seong HanSteven LatosinskyHo-Shik KimSteven M. PascalHossain Mohammad Arif UllahSteven MarkwellHossein TaghizadehSteven NarodHouda BenhelliSteven PattersonHoward A. YoungSteven RosenHoward DonningerSteven StackerHoward E. BoudreauSteven W. RemmengaHoward H. YangSteven W. ThorpeHoyeon LeeSteven ZielskeHridaya ShresthaStig LinderHridayesh PrakashStijn J. De KeukeleireHsiao-Tien LiuStratigoula SakellariouHsin Ling HsuStrippoli SabinoHsin-Chen LeeStyliani GeronikolouHsin-Ell WangStyliani MantziariHsin-Pai LiStylianos KorasidisHsin-Yao WangStylianos TzedakisHsin-Ying Clair ChiouSu Hwan KimHsiyuan HuangSu Jung Oh-HohenhorstHsuan LiuSuba RajendrenHsuan-Ying HuangSubbaramiah SridharHsueh-Ju LuSubhash TripathiHsueh-Wei ChangSubhayan ChattopadhyayHsuheng YenSubhrajit SahaHu ChenSubin Thenkunnel SurendranHua JinSubramanian VenkatesanHua XiaoSubramanya PandruvadaHua ZhuSubramanyam DasariHuaitian LiuSubrat BhattamisraHua-Jun HeSubrato BharatiHuarong ChenSuchandrima SahaHua-Sheng ChiuSuchira GallageHubert Van den BerghSuchismita RahaHuei-Lung LiangSudha SharmaHuei-Tzu ChienSudha SundarHuey-Jen LinSudhir Gopal TattikotaHugh MaSudhir KumarHugo Arasanz EstebanSudip BanerjeeHugo Teixeira-FarinhaSudip MukherjeeHugues Allard-ChamardSueli Mieko Oba ShinjoHui DaiSugato HajraHui LyuSuguro KadamotoHui WangSuguru YamadaHuiching ChuangSuhas V. VasaikarHui-Ching ChuangSuisui HaoHui-Hua HsiaoSujit SuklabaidyaHuihui FanSukanya SahaHui-Li WongSukhbir KaurHui-Lin WuSukkum ChangHui-Ming LinŞükrü ÇağlarHuiming LuSule CanberkHui-Yi LinSulev KoksHunan L. JulhakyanSuman SamantrayHung ChangSumedha BaggaHungcheng LaiSumeet NayakHung-Chi ChengSumeet SolankiHunglung KeSumit MukherjeeHung-Pin TuSun Wook ChoHusam QanashSunali MehtaHwei OngSunao ShojiHye Jin YouSuncana Simonic-KocijanHye Seon KangSuncica BuljevicHyemin LeeSundeep SinghHyeong-Geug KimSung Hoon SimHyeongwon YuSung Jun MaHyo Jung ChoSung-Eun LeeHyo Jung ParkSung-Hoon KimHyock Ju KwonSungmin KangHyoeun ShimSung-Suk SuhHyo-Geun ChoiSung-Wuk JangHyo-Jong LeeSun-Hee LeemHyun ChangSun-Ho LeeHyun Ho YoonSunil KarhadkarHyun Jin KwunSunil LeeHyun Jo YounSunil MaloniaHyun Uk KimSunil S. BadveHyun-Cheol KangSunil SinghHyung Joon YimSunilgowda Sunnagatta NagarajaHyungju KwonSunit DasHyungseok JangSunitha NagrathHyung-Soo HanSunjung YoonHyunjin KimSun-Young KongHyun-jong JangSuprabhat MukherjeeHyunsuk ShimSupreeta AryaHyun-sun ParkSupriya ChakrabortyIakovos AmygdalosSupriya GuptaIan LianSuranganie F. DharmawardhaneIan R. D. JohnsonSurasak SangkhathatIan S. HagemannSuresh KalathilIbrahem KandelSuresh Kumar BalasubramanianIbrahim M. SayedSurya KantIbtissam AcemSusan A. KrumI-Cheng LeeSusan BellisIchidai TanakaSusan BrooksIchiro TakadaSusan BurchillIda ParisSusan C. ShortIdo WolfSusan E. LaFlammeIdoia GallegoSusan HagenIdoia Martín-GuerreroSusan L. NeuhausenIdris RajiSusan RazvanIeva StundienėSusan RichterIfeanyi Jude EzeonwumeluSusana B. BravoIgea D'AgnanoSusana García-SilvaIgnacija VlašićSusana Iraola-GuzmánIgnacio Campillo-MarcosSusana LechugaIgnacio RetamalSusanna GalloIgnacio Vazquez-GarciaSusanna NuvoliIgnazio CondelloSusanne FüsselIgnazio Gaspare VetranoSusanne HafnerIgnazio StanganelliSusanne MalanderIgor AurerSusanne MerkelIgor B. RoninsonSushant BangruIgor BadoSusheel Kumar NethiIgor KhatkovSushil DubeyIgor KovalchukSushma YadavIgor M. PaivaSushmita Bose NandyIgor PiotrowskiSusmita BarmanIgor SirákSusumu GoyamaIhar VolkauSusumu MatsukumaIhtisham BukhariSusumu OhyaII HormuthSuzanne Egyhazi BrageI-Ju YehSuzanne G. OrchardI-Jung TsaiSuzie ChenIkchan JeonSvein Olav BratlieIker UriarteSven Arke LangIkuo ShojiSven MånssonIkuya MiyamotoSven Marcel StefanIla PantSven StadlbauerIlaha IsaliSvetislav TatićIlana Miriam SchlamSvetlana S. DokudovskayaIlaria AttiliSvetlana TamkovichIlaria ColomboSvetlana V. SelivanovaIlaria Elena PalamàSvjetlana MohrmannIlaria GirolamiSvjetlana RausIlaria OttonelliSwanand KoliIlaria PergoliniSwapna ThotaIlaria SaltarellaSwapnil Ganesh SanmukhIlaria TamagnoSwaroop Kumar PandeyIldiko SzantoSwati GargIldikó UnkSwee Keong YeapIleana BaldiSyarida Hasnur SafiiIleana RubioSyed A. QuadriIlias MaglogiannisSyed Ahmad Chan BukhariIlias SkeparniasSyed Azmal AliIljin KimSyed Furqan QadriIlkka IlonenSyed MahmoodIlla TeaSylvain CarrasIlona ArgirionSylvain G. BourgoinIlona KovalszkySylvain GarciazI-Lun HsinSylvain LoricIlya KirgnerSylvain PeugetIlya YakavetsSylvia CrowerIlze ŠtrumfaSylvia L. AsaIman M. TalaatSylvia Le DévédecImtiaz A. SiddiquiSylvie AmuIn Ja ParkSylwia Dzięgielewska-GęsiakIn Rae ChoSylwia Wieder-HuszlaInas FarisSzu-Chia LiaoIndra D. SahuSzymon JanczarIndra J. DasSzymon SkoczeńInes BaroneSzymon SkoczyńskiInes MármolT. Jeroen N. HiltermannInês SantosT. M. De ReijkeIngalill HallbergT. Rinda SoongInge Marie SvaneTabito KinoIngeborg RežuchováTabussam TufailIngo B. RunnebaumTadakazu KondoIngo GanzlebenTadao IshidaIngrid GarajováTadas LatkauskasIngrid HerrTadashi SakaguchiIngrid HickmanTadashi WatabeIngrid Tonning OlssonTadeusz RobakInigo Martinez-ZubiaurreTae Hoon RohInna ChervonevaTae Hyun KimInnocenza PalaiaTae Keun YooInnokentii E. VishnyakovTae-Don KimIoanina ParlatescuTae-Hong KangIoanna NikitopoulouTae-Jung KimIoannis AnagnostopoulosTaeryool KooIoannis AnestopoulosTaghi KhoshgoftaarIoannis ApostolopoulosTaha Koray SahinIoannis D. BassukasTahereh JavaheriIoannis FlessasTahir YusufalyIoannis IakovouTai-long PanIoannis IntzesTainya ClarkeIoannis KonstantinidisTaishi HataIoannis MitroulisTajana PavicIoannis NikolakakisTa-Jen LeeIoannis Ntanasis-StathopoulosTakaaki HirotsuIoannis SanidasTakafumi SatoIoannis VamvakarisTakafumi TaguchiIon MîndrilăTakafumi YanagisawaIppokratis MessaritakisTakahide NejoIra JainTakahiro AnzaiIra S. WinerTakahiro EinamaIraia Muñoa HoyosTakahiro HasebeIrena GlažarTakahiro KimuraIrena IlicTakahiro NakajimaIrena ManovTakahiro NoharaIrena MladenovaTakahiro YamasakiIrene AppolloniTakahiro YasuiIrene BiasoliTakahito KatanoIrene BuvatTakamichi ItoIrene EspositoTakashi IwataIrene FerrerTakashi KokudoIrene Lo CignoTakashi MinegishiIrene Martínez-MartínezTakashi NiizekiIrene MinTakashi NomizoIrene NautaTakashi OginoIrene ScaleraTakashi OnoIrene SlavcTakashi SuzukiIrene SzijanTakashi TakedaIrfan A. RatherTakashi WatanabeIrina DruzhkovaTakayuki AnazawaIrina MäurerTakayuki AndoIrina ProninaTakayuki MurataIrina TsibulakTakayuki OhguriIrina V. BureTakayuki OhkuriIrina V. KondakovaTakayuki TakahashiIrina-Iuliana CostacheTakayuki TakedaIris E. EderTakeda TakashiIrit AviviTakefumi KimuraIrmgard Irminger-FingerTakehiro IwataIsaac BrownellTakehiro ShiinokiIsaac Yves Lopes MacêdoTakeji SakaeIsabel Legaz PérezTakemi OtsukiIsabel M. PiresTakeshi HatanakaIsabel MarzoTakeshi ImamuraIsabel PortilloTakeshi MasudaIsabel Relimpio-LópezTakeshi MurataIsabel Soto-CruzTakeshi NiinumaIsabella GuardamagnaTakeshi SakamotoIsabella ParoliniTakeshi SanoIsabella SaggioTakeshi UradeIsabelle Durand-ZaleskiTaketo YamadIsabelle Galy-FaurouxTaketoshi MaeharaIsadora RosaTaku KouroIsaia BarbieriTaku NaikiIsaku OkamotoTakuji OkusakaIsao OtsukaTakuji TanakaI-Shiang TzengTakuma HigurashiIshita ChatterjeeTakuma UoIshwarya SankaranarayananTakumi IshizukaIsidro MachadoTakumi TakeuchiIslam MohamedTakumi YamamotoIsmaheel O. LawalTakuro KoboriIsrael GannotTakuro NakamuraIsrael ManzanedoTakuro NishikawaIssei SaekiTakuto ShimizuIstván KissTakuya KoieItzhak OrionTakuya MaedaItziar AstigarragaTakuya YoshimuraIulia-Ioana Stanescu-SpinuTali FefermanIuliana MarinTally LevyIva KiracTamar C. BrandlerIva ŠkrinjarTamara ČačevIvan BratchenkoTamara Fernández CabadaIvan CundrleTamara GulicIván HenríquezTamara IusIvan IzoninTamás TornóczkyIván M. MoyaTamer AlshafieIvan PariseTamer S. KaoudIvan R. NabiTami CourseyIvan SabolTami RubinekIvan Shterev DonevTamim NiaziIvan VellaTammam AbboudIvan Y. IourovTania CapeloaIvan ZhovannikTania TriantafyllouIvana KholováTanja LangsenlehnerIvana PeranTanja MarinkoIvana SamarzijaTanja Nicole HartmannIvaylo B. MihaylovTanmoy MondalIveta SelingerovaTanpreet KaurIvonne NelTanuj PuriIvy ChungTanujit DeyIwona ChlebickaTanushree DangiIwona Homa-MlakTanya BisselingIwona ŁugowskaTanya N. SolimanIwona PałygaTao FuIwona Rospond-KubiakTao HuangIzabela ChmielewskaTao LiIzabela ŁasińskaTao YiIzabela ZakrockaTao YinIzabella Slezak-ProchazkaTao ZhangJ. Calvin KouokamTaobo HuJ. Mark SloanTara BaetzJ. Michael SorrellTara RobertsJ. Sven D. MieogTarek ElghetanyJaana OikkonenTariq BhatJacek GębickiTaro OkayamaJacek KiezunTarun UpadhyayJacek MalejczykTasleem ArifJacek R. WilczyńskiTat San LauJáchym RosendorfTatjana ArsenijevicJacinta SimmonsTatjana Crnogorac-JurcevicJack F. ShernTatjana MitrovićJack JiangTatsuhiko ArafuneJack LawlerTatsuhiko FurukawaJaco KraanTatsuhiro SatoJacoba Van Der ZeeTatsuji MizukamiJacopo FalcoTatsuki IkomaJacqueline CloosTatsuki KataokaJacqueline M. BentelTatsunori MinamideJacqueline Schoumans PouwTatsuo KandaJacqueline T. BrownTatsuo KidoJacques BarbetTatsuo OyakeJacques BernierTatsuo SawadaJacques GilloteauxTatsuo ShimosawaJacques RouanetTatsuya AbeJacques ZimmerTatsuya AbéJadwiga ManiewskaTatsuya ToyokawaJae ChoTatsuyuki ChiyodaJae Hee ChoTatu RojalinJae Hyuck ChangTavan JanvilisriJae Myoung NohTavarekere N. NagarajaJae Young HurTea Lanišnik RižnerJae-Ho LeeTed McDonaldJaehong KimTeddy Weifa YangJaehyeon ParkTeemu MurtolaJaejun LeeTeh-Ia HuoJaesang KimTeiji KuzuyaJaesok YuTeizo YoshimuraJaewoo KwonTejas KarhadkarJae-Yong KwakTejeshwar RaoJaeyun WangTeka KhanJaffer A. AjaniTe-Ling LuJagadheshwar BalanTemesgen SamuelJagadish HosmaniTengguo LiJaganmay SarkarTengteng YuJagathesh Chandra RajendranTeodora KolarovaJai Young ChoTeodora TelecanJaideep SandhuTerence K. W. LeeJaime EscalanteTeresa BrownJaime EscallonTeresa CatalanoJaime FeliuTeresa CejalvoJaime Santoyo-SalazarTeresa Lobo-JarneJaime Tomé-AmatTeresa MagnesJainil ShahTeresa PerraJakob IzbickiTeresa Rubio-TomásJakob TroppmairTeresa SommaJakoba J. EertinkTereza GrimmichováJakub DobruchTerrance G. JohnsJakub HofmanTerrence PivaJakub Kła̧czTerri S. ArmstrongJakub KryczkaTerry C. F. YipJakub NalepaTerry L. NgJakub PawlikowskiTerry LichtorJakub RokTeruki YanagiJames AngelastroTerumoto KoikeJames BeirneTessa Le LargeJames C. KnightTessho MaruyamaJames C. NicholsonTetiana SkrypetsJames Chung-Wai CheungTetsufumi TakahashiJames D. BrooksTetsuhiro YoshinamiJames HicksTetsuji OhyamaJames Janopaul-NaylorTetsuya IkedaJames Kumi-DiakaTetsuya OgawaJames LeighTetsuya TakikawaJames NagarajahTetsuya TsukamotoJames P. BasilionTeuta MuratiJames PhangTevhide Ozkaya-AhmadovJames Romero-MastersThagaard JeppeJames S. MiserThai AleshaJames SolomonThangaraju MuthusamyJameson ForsterThanh Hue DellingerJamey WeichertThanigaivelan KanagasabaiJamie Nicole AnastasTheo DemerathJamil MahmudTheo KrausJamila FaivreTheodora KatsilaJan B. VermorkenTheodora PapamitsouJan BewersdorfTheodora PappaJan BorggrefeTheodore LawrenceJan BouchalTheodore S. LawrenceJan Christian KaiserTheodoros KaralisJan CoburgerTheodoros KarantanosJan DouglassTheodoros RampiasJan EggerTheodoros TokasJan FrankoTheodossis A. TheodossiouJan GodzinskiTheresa FrisanJan HaukeTheresa GuoJan HrbacekTheresa SteebJan HrudkaTherese BeckerJan J. W. LagendijkTherese SeierstadJan KomorowskiTherese SörlieJan KorbeckiTherese WilhelmJan KotarskiTheresia ThalhammerJan Krzysztof NowakThibault MazardJan L. C. LoeffenThiemo DingerJán LakotaThierry AlcindorJan MejzlikThierry CapiodJan NovákThierry LecomteJan PilchThierry MagnaldoJan PlzakThierry PietersJan ŠevčíkThiha AungJan StȩpniakThinzar M. LwinJan TrnaThirumal RajJan Van MeerbeeckThomas BachelotJan Von Der ThüsenThomas BartlJan Willem B. de GrootThomas BottonJan ZauchaThomas BrogginiJan ZedeniusThomas CaspariJan ŽenkaThomas CunyJana L. FoxThomas CzechJana SmahelovaThomas DaubonJanaina FernandesThomas DeckerJanakiram R. VangalaThomas E. AhleringJaneen TrembleyThomas E. SchmidJanet OlsonThomas EggermannJanez RavnikThomas GreitherJani SilvaThomas HankJanielle P. MaynardThomas J. Fahey IIIJanik PuttemansThomas J. W. Klein NulentJanin ChandraThomas John MartinJanina Baranowska-KortylewiczThomas KerkhofsJanina MarkowskaThomas L. OlsonJan-jong HungThomas LichtJanković Miljuš JelenaThomas LiehrJános KappelmayerThomas ManningJanos MinarovitsThomas MatthesJanoš TerzićThomas Matthijs Van GulikJan-Paul GundlachThomas MawhinneyJan-Peter GrunzThomas MayrJanusz StrzelczykThomas MelendyJan-Willem BeenakkerThomas MohrJaques SimardThomas MürdterJaques Van HeerdenThomas OttoJared BarrottThomas PabstJared FischerThomas R. ChaunceyJared J. BarrottThomas ReinheckelJared T. AhrendsenThomas SchubertJari LouhelainenThomas SuttonJarlath Christopher BolgerThomas TschernigJarmila KruseovaThomas VielJaromir AstlThomas WeberJaroslav PejchalThorsten BraunJarosław ĆwikłaThorsten RosenbaumJarosław CzyżThorsten SimonJaroslaw MacIaczykTiago RodriguesJarosław PaluszczakTiancai LiuJaroslaw RegułaTianfang MaJar-Yi HoTianhui HuJasbir UpadhyayaTianjie PuJasjit S. SuriTian-Li WangJasmina KuvendjiskaTianpeng ZhangJasmina StojkovskaTiantian ZhangJasmine M. ZainTianyi QianJasna MetovicTiberiu Paul NeaguJasna MihailovićTibor HianikJason ChanTibor Valyi-NagyJason HannaTierry ConroyJason NortheyTiffany N. SeagrovesJason ParsonsTihana DžombetaJason RoszikTiit MathiesenJason TasoulasTijana Jovanović-TalismanJason X. ChengTijl VermassenJason Yongsheng ChanTill Jasper MeyerJasper NijkampTilmann BochtlerJaume GrauTim AhlesJaume MoraTim HilgenfeldJavad NazarianTim PrangemeierJaved AhmadTim R. GlowkaJaveed ShaikTim ReeseJavier AlonsoTim RippergerJavier C. AnguloTimofei ZatsepinJavier GarzónTimothée ZaragoriJavier LaverniaTimothy A. RitzmannJavier LeonTimothy C. HallstromJavier LouroTimothy CorsonJavier Martinez-UserosTimothy GershonJavier Menéndez-MenéndezTimothy HasenoehrlJavier Molina-CerrilloTimothy KwaJavier OsorioTimothy M. KuzelJavier P. GisbertTimothy M. NyweningJavier Redondo-MuñozTimotius Ivan HariyantoJavier Rodríguez RodríguezTin PhanJavier RosTina Batista NapotnikJaw-Yuan WangTina Di PalmaJay AnandTina Nørgaard MunchJaya Prakash GollaTina Tičinović KurirJayakumar NairTineke E. BuffartJayakumar PerumalTing Martin MaJayalakshmi KrishnanTing ZhongJayapal Reddy MallareddyTing-Feng WuJayasimha Rayalu DaddamTingjin ChenJayoung HanTingting ShiJean De la RosetteTing-Wen ChenJean Michel MauryTingxin ZhangJéan Pierre GérardTingyu ChangJean PinsonTing-Yu LinJean SeelyTithi BiswasJean-Baptiste OudartTiziana AngrisanoJean-Francois BeaulieuTiziana De CristofaroJean-François EmileTiziana FeolaJeanine RoodhartTiziana VenesioJean-Jacques TuechTiziano MarzoJean-Luc RaoulTobias BäuerleJean-Marc BarretTobias EttlJean-Marie RuysschaertTobias F. DreyerJeanne A. JordanTobias MaurerJeanny B. Aragon-ChingTobias PiegelerJean-Philippe FoyTobias ReiffJean-Philippe LambertTodd M. PittsJeanpierre DrozToh Tan BoonJean-Pierre GagnerTohru NakagawaJean-Pierre MazatTokameh MahmoudiJean-Yves BlayTom J. SnijdersJee Hyun KongTomar GhansahJeff SandsTomáš BüchlerJeffrey A. JonesTomas KronJeffrey A. ThompsonTomas NovotnyJeffrey BuchsbaumTomás Pascual MartínezJeffrey DamrauerTomáš TomášJeffrey E. RubnitzTomas VacikJeffrey F. ScottTomasz DzierżanowskiJeffrey GrahamTomasz GradalskiJeffrey MacdonaldTomasz GrzywaJeffrey SchlomTomasz HirnleJeffrey V. LeytonTomasz KluzJe-Kyung RyuTomasz KosmalskiJelena DinićTomasz KubrakJelena GrahovacTomasz LepionkaJelena KnezevicTomasz MarjanskiJelena Korac PrlicTomasz PowrózekJelena LazicTomasz RutkowskiJelena MusulinTomasz SkrzypekJenchih TsengTomasz W. RutkowskiJen-Chih TsengTomasz W. TurowskiJenifer ProsperiTomasz WentaJenna SopfeTomasz WróbelJennifer B. ValerinTomaž ŠtupnikJennifer D. WuTomer FeigenbergJennifer Ellen LeeTomio NakayamaJennifer FazzariTommaso De MarchiJennifer H. ChoeTommaso FabrizioJennifer HaynesTommaso GrecoJennifer L. MeitzlerTommaso Lorenzo ParigiJennifer L. WestTommy AlainJennifer M. KalishTomoaki UedaJennifer MytychTomoaki YohJennifer PursleyTomoharu YoshizumiJennifer SugaTomohide TsukaharaJennifer WangTomohiro ChibaJenn-Ming YangTomohiro EnokidaJenny BulgarelliTomohiro F. NishijimaJenny L. PerssonTomokazu OhishiJenny SherriffTomoki NakamuraJenny-Maria JönssonTomoki NaoeJens HahneTomomitsu MiyagakiJens JakobTomonori OkaJens KleesiekTomoshige KinoJens Thomas Fredrik OsterkampTomotaka UgaiJenu ChackoTomoya MaedaJen-Yin ChenTomoya YokotaJeon LeeTomoyo OkadaJeong A. ParkTomoyuki KoikeJeong Eun KimTongbao LiuJeong-An GimTongtong LuJeong‐Hoon LeeToni SeppalaJeong-Hwa WooTony L. T. NgJeongmin LeeToos DaemenJeong-yeon LeeTorben GlatzJeremie VitteTorben RedmerJeremy ChienTorstein R. MelingJer-Hao ChangTorsten KesslerJeroen KrijgsveldTorta RiccardoJeroen T. BuijsToru HiragaJerome LacombeToru HirozaneJérôme MoreauxToru IshikawaJerome TannerToru SugiyamaJérôme TorrisaniToru TatenoJeronimo BravoToshiaki OharaJerry XiaoToshihiko MatsumotoJerson L. SilvaToshihiro NishizawaJer-Yiing HoungToshimitsu YamaokaJerzy ChudekToshinori SoejimaJerzy MosiewiczToshio OhashiJes-Niels BoeckelToshiro OguraJesse PlascakToshiya TanakaJessica CarlssonToshiyuki HoriJessica Da Gama DuarteToshiyuki OkumuraJessica Dal ColToshiyuki SakamakiJessica IorioToshiyuki ToshitoJessica KarlssonToshiyuki YonedaJessica LangToti LucaJessica LockeryTou-Pin ChangJessica PollardToyonori TsuzukiJesús Campos-GarcíaTracey Di SipioJesus Delgado-CalleTracy G. LivelyJesus Duque-AfonsoTraian DumitraşcuJesús García-CanoTrang NguyenJesus Garcia-FoncillasTravis C. SalzilloJesús Jiménez-LópezTrinidad Montero-VilchezJesús María Hernández RivasTrisha BansalJesús Miguel García-FoncillasTrynda JustynaJesus S. JimenezTsai-Ching HsuJet BliekTse-Ching ChenJi Hoon JungTsuguru HayashiJi WangTsukasa IkeuraJia GuoTsung-Hua HsiehJia LiuTsung-I HsuJia WuTsung-Jung LiangJia Xian LawTsung-Min HungJiabao XuTsutomu NakazawaJiachen ZhuoTsutomu NishidaJia-Ching ShiehTsutomu ShimuraJiafen HuTsu-Yi ChaoJia-Feng ChangTsuyoshi KojimaJiahao ChenTsuyoshi OhtaJiajia WangTsuyoshi TajimaJiajia ZhouTsvetelina VelikovaJian LiTsz-Lun YeungJian WuTuba Çiğdem OğuzoğluJian XuTuba GideJian ZhangTudor PopJian ZhouTullio FlorioJianbo CaoTullio PiardiJianfeng HuangTung Yu TsuiJianfeng LiTung-Hung SuJian-Hua MaoTuo ShaoJianhua YuTuoen LiuJianing XiTvrtko HudolinJianjian LiTze-Chien ChenJianjun RenTzeon-Jye ChiouJianli TaoTze-Ta HuangJianling BiTzipora S. Karin Eisinger-MathasonJianling XieTzu-Ting HuangJianming WangTzu-Yen HuangJianping LuUberto Fumagalli RomarioJian-Qiang LiuUdaiyappan JanakiramanJianqiang XuUdayan GuhaJianqin LuUdesh DhawanJianru XiaoUdo KontnyJianting ShengUdo SchumacherJianyu RaoUffe BødtgerJianyuan ChaiUffe Heide-JørgensenJianzhou CuiUgo MarcheseJiao WangUgo MoensJiaquan YuUgo TestaJia-Shiun LeuUhi TohJiawei WangUiju ChoJiayue QinUjjal Kumar BhawalJibran Sualeh MuhammadUlf D. KahlertJie GaoUlf DettmerJie JiangUlf TitzeJie LeeUlises UrzúaJifke VeenlandUlla Birgitte VogelJihe ZhaoUlla KlaiberJiheum PaekUlrich PfefferJi-Hong LimUlrich PilatusJihwan HaUlrich SackJijun HaoUlrich WellnerJill Marie KolesarUlrich WirthJill P. SmithUlrike BrüningJim HoltUlrike HegerJim Jinn-Chyuan SheuUlrike WielandJimin ShaoUmang SwamiJimma LenjisaUmberto BaccaraniJin GanUmberto MalapelleJin Hyoung KimUmesh Tharehalli MathadaJin Sung JangUn Yung ChoiJin WangUpasana RayJin Woo ChoiUrban ŠvajgerJin Wook ChungUri NirJin Wook KimUroš BumbaširevićJin ZhangUrsula Maria VoglJin ZhongUrsula QuittererJinae LeeUrszula HohmannJinbiao ChenUrszula OleksiewiczJinbin XuUrszula SmyczyńskaJinchao LouUthayashanker R. EzekielJindong XieUttam SatyalJineta BanerjeeUttara BasuJing ChenUwe KlingeJing ChengUwe SchlegelJing FangVadanasundari VedarethinamJing HuangVadim ByvaltsevJing LiuVadim GaponenkoJing MaVahid Afshar-KharghanJing YuanVahid HaghpanahJinglin ZhangVaidotas StankevičiusJingting YuVaishali AggarwalJing-Wen ShihVaishnavi MuralikrishnanJingyi ChiVakul MohantyJingying ZhouValentin BarthetJingyu FanValentin Nicolae VarlasJin-Hwi KimValentina AmbrosiniJinqiu LuValentina BrancaleoniJin-Tung LiangValentina CalòJin‐Wook KimValentina CirelloJinxing YuValentina ComunanzaJin-Yih LowValentina CossigaJinzhou ZhuValentina De FalcoJiong LiValentina De NunzioJiong ZhouValentina DiniJiří KillerValentina DoldiJiri PolivkaValentina Elisabetta BounousJiri VachtenheimValentina FaustiJiřina BartůňkováValentina GianniniJiro KikuchiValentina MelocchiJit Kong CheongValentina PileczkiJiten JaipuriaValentina PozziJitka MalčíkováValentina S. CaputoJiunn-Liang KoValentina TodorovaJiun-Wen GuoValentine ComaillsJiwang ZhangValentine SuteauJiyoung LeeValeria BarresiJiyue ZhuValeria BertagnoloJi-Yun LeeValeria De PasqualeJo Erling Riise WaageValeria LucariniJoachim HohmannValeria LucchinoJoachim LupbergerValeria MerzJoachim NoldusValeria NaimJoachim WiestValeria PecceJoan GilValeria RomeoJoan HowValeria SebriJoan SeguraValérie BillardJoana CaetanoValerie Bracchi-RicardJoana CarvalhoValerie CopiéJoanna A. MusialikValérie CoronasJoanna BarankiewiczValerie G. BruntonJoanna BialekValerie McLaughlin CrabtreeJoanna Darck Carola Correia LimaValerie SpeirsJoanna Domagala-KulawikValerio CecinatiJoanna Drozd-SokołowskaValerio CostaJoanna Katarzyna StrzelczykValerio Di PaolaJoanna Klećkowska-NawrotValerio D'OraziJoanna KozakValerio NardoneJoanna L. FoxValerio PazienzaJoanna ŁudzikValeriu LupuJoanna PrzybylValeriu SurlinJoanna SochaValéry SalleJoanna StefanowiczValter GatteiJoanna WietrzykVanathi PerumalJoanna ZawitkowskaVanda Maria AndradeJoanne KotsopoulosVanesa Fernández-SáizJoão Agostinho Machado-NetoVanessa D'AntongiovanniJoão Carlos MacedoVanessa PanettieriJoão LoboVanna Chiarion-SileniJoão M. C. TeixeiraVarsha JainJoão Pinto-De-SousaVartika TomarJoão RodriguesVarun KumarJoao Santos-AntunesVarvara TrachanaJoaquim CarrerasVarvara ValotassiouJoaquim Miguel OliveiraVasile DrugJoaquim PeñaVasileios Panagiotis E. LenisJoaquim Soares Do BritoVasiliki BravouJoaquín María CamposVasiliki DanilatouJoaquín V. Arribas-LopezVasiliki MollakiJob De LangeVasiliki SiozopoulouJocelyn GalVasily YakovlevJocelyn ReaderVasko VasylJochen BaßlerVassili SoumelisJochen H. M. PrehnVassiliki LyraJochen HillerVassilis GeorgouliasJochen MaurerVasu SahJochen PrehnVasyl VaskoJochen WillnerVeedamali S. SubramanianJoe S. MymrykVeena KochatJoe ZhuVeit RohdeJoel NaftalinVenija CeroveckiJoël RaingeaudVenkata Narasimha KadaliJoel YisraeliVenkatakrishnan RengarajanJoëlle RiondVenkataswarup TiriveedhiJoëlle WielsVenkateshwar MadkaJoellen M. SchildkrautVenkateswarlu KanamarlapudiJoerg LindenmannVenkatraman GaneshJohan BengzonVenturina StagniJohan BotlingVera CapelozziJohan NilvebrantVera CappellettiJohan O. PaulssonVera Ulrike BacherJohann GoutVerdiana TrappettiJohann Von FeldenVerena BoschertJohanna KirchbergVerena Keitel-AnselminoJohanna Lena TheruvathVerena TischlerJohanne PoissonVerena WiegeringJohanneke PortieljeVernell WilliamsonJohannes BoettcherVeronica BoyleJohannes CrezeeVeronica Di CarloJohannes F. CoyVeronica GhiniJohannes KraftVeronica MartiniJohannes LaengleVerónica Moreno-ViedmaJohannes LinxweilerVeronika BorutinskaiteJohannes LubkeVeronika Buxhofer-AuschJohannes MeilerVeronika HolubekovaJohannes SchobelVeronika HuntosovaJohannes SchumacherVeronika Lukacs-KornekJohannes VölkerVéronique DiérasJohannes WachVéronique GirouxJohannes WoitzikVéronique Maguer-sattaJohannes ZenkVéronique Serre-BeinierJohari Yap AbdullahVeronique VendrelyJohn A. PetrosVesna I. KesićJohn AlexanderVibha TandonJohn AsaraVic VelanovichJohn AshtonVicente Escamilla-RiveraJohn B. KisielVicente Javier Clemente-SuárezJohn BuolamwiniVicente Seco-RoviraJohn C. RichesVicki ButenschoenJohn ChirgwinVicki Suzanne KlimbergJohn F. NewmanVicki WhitehallJohn H. PyneVictor A. ArrietaJohn HaganVictor B. LoschenovJohn HegdeVictor BanerjeeJohn HeissVictor E. NavaJohn Henry RossmeislVictor G. PrietoJohn J. NemunaitisVictor J. YusteJohn Kim ChoiVictor M. Bolanos-GarciaJohn L ReaganVictor RomanovJohn LaiVictor SierpinaJohn LaterraVictor TreviñoJohn LenehanVictor V. PleshkanJohn M. KaczmarVictor Vlad CostanJohn M. LamarVictor Y. UmanskyJohn M. PerryVictoria Da Silva-DizJohn Maringa GithakaVictoria L. SeewaldtJohn Michael VarlottoVidhi ChandraJohn MiuraVidya GanapathyJohn MonganVidyalakshmi ChandramohanJohn NewellViet-Khoa Tran-NguyenJohn Paul PlazzerVignesh SundararajanJohn R. HawseVignesh VudathaJohn RaskoVijay BoggaramJohn S. MitchellVijay KotraJohn ThomsVijay KumarJohn W. SteeleVijay P. S. RawatJohn W. BirkVijay PandeyJohn W. CrabbVijay ParasharJohn WattsVijaya VaikariJolanta B. ZawilskaVijayachitra ModhukurJolanta DyniewiczVijaysaradhi SetaluriJolanta KiewiszViji ShridharJolanta RzymowskaVikas JhawatJon GlassVikas MalojiraoJon HoutmanVikas YadavJon Salmanton-GarcíaVikash KansalJon WadsleyVikash SinghJonas Amstrup FunderVikram AdhikarlaJonas RistauVikrant RaiJonathan Barroso-GonzálezViktor ViglaskyJonathan BenzaquenViktória KoroknaiJonathan D. HumphriesVilas NasareJonathan ElliottVille PimenoffJonathan GarnierVilma Yuzbasiyan-GurkanJonathan LopezVinay MandatiJonathan MartinelliVincent BourbonneJonathan NoujaimVincent CamusJonathan Robert WeitzVincent H. J. Van Der VeldenJonathan RothVincent KellyJonathan SoVincent MocquetJonathon Edward MohlVincent NjarJone TrovikVincenza BarresiJong Ho YoonVincenzo AbbateJong-Han KimVincenzo AdamoJonghan YuVincenzo CondelloJony Van HilstVincenzo DesiderioJoo Hee YoonVincenzo FiorentinoJoo Ho LeeVincenzo FormicaJoo Hyun OVincenzo LizziJoohee JungVincenzo Manuel MarzulloJoon Hyuk ChoiVincenzo MarottaJoon Young ChoiVincenzo Nicola TalesaJoon-Khim LohVincenzo VillanacciJoost KluiverVineet K. GuptaJoost M. KlaaseVinit ShanbhagJoost Van GriethuysenVinod VijayakurupJordi RiberaVinodh KakkasseryJörg GroßhansViola Maria PopovJörg HaierViolaine RandrianJörg HoheiselVirag VasJörg KleeffVirginia AmadorJörg MüllerVirginia BrancatoJörg Thomas BittenbringVirginie JorisJörg WischhusenVirginie MarcelJorge A. MoralesVirginie MonceauJorge BarbazánVirginie NeirinckxJorge CervantesVirtudes Pérez-JoverJorge LabradorVisalini Nair-ShallikerJorge MartinezVisanu WanchaiJorma A. MäättäVishakha Anand PawarJörn LausenVishal ChandraJos L. EerselsVishal ChavdaJosanne VassalloVishal KhivansaraJosé A. CarriónVishalkumar G. ShelatJosé Andrés YunesVishnu Muthuraj KumarasamyJosé Ángel Díaz-PérezVishnu Priya MuraliJosé Antonio SanchesVishnu RevuriJose Antonio UrangaVita GolubovskayaJosé Bañuls-RocaVita PašukonienėJose Barreto CarvalheiraVitalii LunovJose G. MantillaVitaliy GarginJosé HardilloVito Carlo Alberto CaponioJosé Luis MansurVito Giuseppe D'AgostinoJosé Luis Muñoz De NovaVítor H. AlvesJosé Luis Pardal-RefoyoVitor ParolaJose Luis Perez-GraciaVittoria ColottaJose Luis Rodriguez PeraltoVittoria D’EspositoJosé Luis Romero BéjarVittoria DisciglioJose Luis ZugazaVittorio AprileJosé M. CasanovaVittorio FuscoJosé Manuel Cameselle-TeijeiroVittorio GebbiaJose Manuel LopesVittorio Lorenzo QuagliuoloJose Manuel RamirezVivek AnandJosé María Enríquez-NavascuésVivek YedavalliJosé Maria LassoVivian BarakJosé Miguel Sánchez-TorresViviana BarraJose MonzonVlad PorumbJose PalaciosVlad SandulacheJosé SchneiderVladimir B. AnikinJosé Tapia-RamírezVladimir BabenkoJosé YélamosVladimir BedekovićJosé-Ángel Hernández-RivasVladimir DrozdovitchJosee Guirouilh-BarbatVladimir JurisicJosef Paul KovaříkVladimír KryštofJosef SmolleVladimir KublanovJosefa DomenechVladimir L. SukhorukovJosep J. CentellesVladimir LazarevićJosep Maria Ramon TorrellVladimir MilosavljevicJoseph (Sifis) PapamatheakisVladimir PorochJoseph Daniel ButnerVladimir RogalewiczJoseph KauerVladimir SukhorukovJoseph KwongVladislav YakovlevJoseph M. HermanVlad-Olimpiu ButiurcaJoseph R. PetersonVlasios SotirchosJoseph SiaVolkan ErginJoseph SottnikVolker NeuschmeltingJoseph W. FransesVolkhard HelmsJoseph W. LandryVolkmar MüllerJosephina SampsonVrinda GoteJosephine DorsmanVuyolwethu KhwazaJosé-Tomás NavarroW. Martin KastJoshua BarzilayW. Paul Farquhar-SmithJoshua C. BlackWade IamsJoshua LevyWael HegazyJoshua R. MenkeWael TraboulsiJoshua TobiasWai C. ChongJosip TomićWai Chin ChongJosko BozicWaikin ChanJosue Ballesteros-AlvarezWaisse WaissiJoszef DudasWaku HattaJoy PerrierWaldemar GoldemanJoyce Taylor-PapadimitriouWaldemar ReichJozsef DudasWaldemar WagnerJózsef PiffkóWalee ChamulitratJózsef VirgaWalid Ibn EssayedJr. SmetanaWalid S. AyoubJuan Antonio Fafián LaboraWalid ShalataJuan Carlos De VicenteWalter BergerJuan David Ospina-VillaWalter HanelJuan DuWalter L. StrohmaierJuán Fernando GarcíaWancai YangJuan Gómez RivasWan-Chi TsaiJuan Jesús CruzWangxiao HeJuán Jiménez-SánchezWanjuan FengJuan José Moreno AznárezWanting HanJuan Luis Garcia HernandezWantong YaoJuan Manuel Germán-AcacioWanwen ZengJuan Manuel Muriel-SánchezWanying WuJuan Manuel O'ConnorWaqas BangyalJuan Manuel PovedanoWard De SpiegelaereJuan Manuel Sepúlveda SánchezWaseem LoneJuan MelchorWasim DarJuan P. RodrigoWasim H. ChowdhuryJuan Pablo AlderuccioWayne Owen MilesJuan Pablo NicolaWee Loon OngJuan Pablo RigalliWei DaiJuan Pedro Martínez-RamónWei GuJuan PerdomoWei JiangJuan QinWei KangJuan R. De Los ToyosWei NiJuan Rodríguez-VitaWei TengJuan-Pablo Cerapio ArroyoWei Wen LimJubina VenghateriWei WuJudhajeet RayWei YanJudith DavieWei YangJudith E. Raber-DurlacherWeichao ChangJudith FischerWei-Chi KuJudith HoningWei-Chien HuangJudith KarpWei-Chih ChenJudith OffmanWei-Chyou WeiJudith Raber-DurlacherWeihong JiangJudith SchwartzbaumWei-Hsiung YangJudith Stangl-KremserWei-Hsuan YuJudy M. CoulsonWeihua TianJuei-tang ChengWeijia SuJuergen ReichardtWei‐Jiunn LeeJui-Chieh ChenWeijun QinJukka KanervaWeikun XiaoJukka WestermarckWei-Lun ChangJules DerksWeilun TsaiJules Tianyu Zhang-yinWeimer JörgJulia BarWei-Min ChangJulia ChisholmWeimin HuangJulia FurtnerWeisi LiuJulia Newton-BishopWeisi XieJulia SchuelerWei-Wei DengJulia TietzeWeiwei ZhangJulia WelzelWeiyi XuJulian Freen-van HeerenWeiyu JiangJulian JacobWeiyu KaoJulian KünzelWellington Pinheiro Dos SantosJulian LumWen LiJulian S. RechbergerWen LiuJulian TarandaWen Sy TsaiJuliane Daßler-PlenkerWen Tsan ChangJuliane HoyerWen WangJuliane PutzWen ZhouJuliano D. PaccezWen-Chi YangJulie DardareWen-Chien TingJulie HelftWen-Chun ChangJulien BecWenchun ChenJulien EdelineWendell G. YarbroughJulien ForestierWendell YarbroughJulien VignardWendy Demark-WahnefriedJuliet GrayWeng-Onn LuiJuliet Haarbauer-KrupaWen-Hao YangJulieta Alexandra Pereira AfonsoWen-Hua ZhengJūlija VoicehovskaWenjie WangJulius HöhneWenjun WangJun ChenWenjun WuJun ChengWen-Kai WengJun GuWen-Kuan HuangJun ImaiWen-Ling KuoJun ItamiWenning WangJun MaWensi TaoJun NakamuraWen-Wei SungJun NishikawaWenxin TongJun SunWenyan XuJun WangWenyi LuoJun WeiWenyue SunJun WuWerner T. W. De RieseJun XiaWeronika RatajczakJuneko E. Grilley-OlsonWiebke SolaßJunfeng MaWiesława LeśniakJung Su RyuWilfried De NeveJung Sun YooWilli SauerbreiJung Woo EunWilliam A. DennyJung-ho YoonWilliam C. FaquinJung-Soo PyoWilliam F. BlakelyJun-ichi HanaiWilliam FiggJun-ichi SawadaWilliam H. GmeinerJunjiang SunWilliam J. BrackenburyJunjie WangWilliam J. MurphyJunko YamamotoWilliam JacotJunlei ChangWilliam JiaJunli ZhaoWilliam L. IrvingJunling ZhuangWilliam RaoulJunpei YamaguchiWilliam T. BeckJunqi HeWilliam TanseyJunsheng PengWilliam VermiJun-Te HsuWillie HamiltonJunxuan LuWim P. CeelenJunyan TaoWinald GerritsenJunying MaWing Kee LeeJuraj PrejacWing Lok ChanJure KnezWing-Keen YapJure UrbancicWioleta KowalskaJürgen BeckerWitold GertychJurgen DittmerWładysław JanuszewiczJürgen DunstWojciech BalJürgen GeislerWojciech CytawaJürgen GrünbergWojciech JelskiJürgen SchlegelWojciech KrajewskiJürgen ThomaleWojciech LeppertJurjen VersluisWolfgang E. BerdelJurriaan TuynmanWolfgang FreysingerJustin HwangWolfgang LinkJustin T. MoyersWolfgang Michael BruecklJusto Lorenzo BermejoWolfgang MikulitsJustus BaumgartenWolfgang WeberJustyna CzaplaWolfram Trudo KnoefelJustyna HermanowiczWonjoong CheonJustyna KowalskaWonmo SungJustyna MiszczykWonsuck YoonJustyna Niderla-BielińskaWonsuk YooJustyna Walczak-SkierskaWoo Kyung JeongJutta HübnerWoo LeeJuuso PaajanenWoohyung LeeJyoti Bala KaushalWoojin KimJyoti SrivastavaWoosung KwonK. J. Senthil KumarWouter R. P. H. Van De WorpK. S. Clifford ChaoWyanne A. NoortmanK. Scott WeberWytske Van WeerdenKa YangXanthopoulos KonstantinosKa Yu TseXavier Sastre-GarauKaarel AdambergXavier ThomasKah-Hui WongXavier ThuruKai DobersteinXavier TroussardKai GuoXavier X. Sastre-GarauKai KrögerXenia PenateKai TaoXia WeizhongKai WangXiaming LiuKai Wen HuangXianda ZhaoKai YuXiandong LengKaidong WangXiang Da Eric Da DongKai-Jun LuoXiang Qian SuKai-Lee WangXiang RenKaisa SunelaXiang ShuKai-Wen HsuXiang WangKajal BiswasXiang ZhaoKalaiselvi SenthilXiang ZhouKaliński PawełXiang-Ping ChuKalkunte SrivenugopalXiangping YangKalle MattilaXiangsheng ZuoKallesh D. JayappaXianjun FangKalliopi PlatoniXianlong GaoKam C. YeungXianwen WangKamal JoshiXiao Fei LiKamal Kant SahuXiao NiKamal MandalXiaobing WangKamariah IbrahimXiaobo ZhongKamijo TakehikoXiaodong ZhangKamil GrubczakXiaofeng Allen SuKamil KrawczyńskiXiaofeng QiKamil M. FramXiaofeng TaoKamil NelkeXiaohe YangKamil WdowiakXiaohong LiKamila Maliszewska-OlejniczakXiaohong ShuKamila PolgarovaXiaohu HuangKamila Środa-PomianekXiaohui LiuKanae MureXiaohui ZhangKanae TarunoXiaokang LunKanakaraju ManupatiXiao-Kun OuyangKang Hao CheongXiaolan DengKankadeb MishraXiaoli PingKapil SaxenaXiaoli TangKapil SirohiXiaoli ZhangKara GavinXiaolin NanKarel PacakXiaolin ZhuKarem A. CourtXiaolin ZiKaren A. BoehmeXiaolong YanKaren ChiamXiaoming ZhengKaren CooperXiaona YouKaren KopciukXiaoping PuKaren LibyXiaoqi LinKaren McLeanXiaoqian ChengKaren TanXiaoqiang WangKarijn SuijkerbuijkXiaoting HongKarim GasmiXiaoting ZhangKarim Thomas SadakXiaoxiao HuangKarin Müller-DeckerXiaoxiao SunKarin StålbergXiaoyan DuKarin StrigårdXiaoyan WangKarin SundfeldtXiaoyin LiKarin WallanderXimena P. VergaraKarina KulangaraXin LaiKarina VargovaXin LiKarine MichaudXin SunKarisa C. SchreckXin WangKarita PeltonenXin ZhangKarl FöhrXin ZhouKarl MungerXinan WangKarl Reinhard AignerXinbin GuKarl Wilhelm W. EckerXinchen WuKarlena Lara-OteroXinfang YuKarl-Heinrich LinkXingchen PengKarl-Michael SchebeschXingyu ShenKarmela BarisicXingyu WuKarmele ValenciaXinmin ZhangKarol Rawicz-PruszyńskiXinran LiuKarola Vorauer-UhlXinyu WangKarolien GoffinXiuhua WengKarolina GołąbekXiuhui ChenKarolina Gregorczyk-ZborochXiuning LeKarolina H. Czarnecka-ChrebelskaXiuwei YangKarolina KędraXiyuan LuKarolina KowalskaXuan ZhangKarolina ŁuczkowskaXuan-Mei PiaoKarolina MarkietXue WangKarolina OsowieckaXuefei TanKarolina PavicXuefeng LiuKaroly SzuhaiXuehui PangKarsten GeletnekyXuekui ZhangKarsten SchöllerXuelin LouKartika Afrida FauziaXue-Song LiuKashi Raj BhattaraiXuewen DengKashif ShamimXuezhao LiuKaspar BurgerXuyao ZhangKasper M. RouschopY. O. DavydovaKasturi BanerjeeYa-Ching ChouKasturi RangannaYafei WangKata JuhaszYafeng LiKatalin DobraYafeng MaKatalin MeszarosYahong WuKatalin SzásziYa-Ju HsiehKatarina TrajkovićYa-Ling HsuKatarzyna ArczewskaYamei TangKatarzyna BanachYamin LiKatarzyna DerwichYan DongKatarzyna Dobruch-SobczakYan DuKatarzyna HojanYan LiKatarzyna KoślaYan YangKatarzyna Kucwaj-BryszYan YuanKatarzyna KuśnierzYan ZhuKatarzyna KwiatkowskaYana G. NajjarKatarzyna MnichYanfeng GaoKatarzyna OwczarczykYang ChenKatarzyna PierzchalaYang KailuKatarzyna Pietraszek-GremplewiczYang LiKatarzyna PogodaYang MeiKatarzyna SędłakYang TongKatarzyna WiktorskaYang YangKate AndersonYang Yang-HartwichKate McBrideYang ZhaoKate VandykeYang-Gun SuhKaterina GiotiYangpeiwei HuangKaterina GrafanakiYangsean ChoiKaterina StratiYanguang CaoKaterina-Marina PilalaYangyiran XieKatharina EffenbergerYan-Hong CuiKatharina Harms-EffenbergerYanhong DengKatherine ElferYanina Jeanne Leona JansenKatherine KarakoulaYanis Toledano-MagañaKatherine T. MorrisYanji XuKathleen (Katie) DorrisYanjie ZhangKathleen LeemansYan-Jye ShyongKathleen M. SakamotoYanling LiaoKathleen PishasYan-Ming XuKathleen W. ZhangYann GuérardelKathryn AlsopYannick ViossatKathryn E. BanfillYanqiang LiKathryn E. RoyseYan-Ruide LiKathryn KnoopYanyu HuangKathryn LeyvaYanyun GaoKathy GatelyYao JiangKathy TaylorYao-Ching HungKati ErdmannYaohua YangKatia GagneYaowu HeKatia MesquitaYara M. MichelacciKatia PagnanoYariswamy ManjunathKatia PinelloYaroslav O. MezhuevKatie DevineYaroslav TeperKatie F. LovesonYaroslava KarpovaKatie SnapeYasmin CypelKatja GoričarYasser GedKatja HarbstYasser HeakalKatja SeipelYasser MajeedKatja SteigerYasuaki TamuraKatja VetvikYasufumi MurakamiKatrin ScheinemannYasufumi OmoriKatrina O'HalloranYasuhiko SugawaraKatrina VanuraYasuhiko TachibanaKatrine RiklundYasuhiro FujisawaKatsuhiro ItoYasuhiro KuroiKatsuki TsuchiyamaYasuhiro OkumuraKatsutoshi OdaYasuhisa FujiiKatsuya YamadaYasumasa OkazakiKatsuyuki MiyabeYasunari MatsuzakaKausik ChakrabartiYasuni NakanumaKavindra K. KesariYasunori MinamiKavita RawatYasunori OtowaKavya AnnuYasunori TomeKa-won NohYasunori YaoitaKayani KayaniYasuo IwadateKazimierz KarolewskiYasushi HasegawaKazimierz KuliczkowskiYasushi HiraokaKazuhide OhtaYasushi TsujiKazuhiko HashimotoYasutaka KakinokiKazuhiko KotaniYasuto KinoseKazuhiro IkedaYasutoshi KimuraKazuhito SuzukiYasuyuki S. KidaKazuko Kaneda-NakashimaYatao RenKazuma OgawaYawei LiKazumasa MatsumotoYe TianKazumichi KawakuboYegor S. VassetzkyKazunari SasakiYen Chien LeeKazunori NagasakaYen Y. TanKazunori TobinoYen‐Chein LaiKazuo KoyanagiYen-Chien LeeKazuo KubotaYen-Chun PengKazuo NakanishiYenkai LimKazutoshi FujitaYen-Lin LiuKazuya InokiYeong Chan LeeKazuyasu FujiiYeonjung HaKazuyuki ItohYeon-Sun SeongKazuyuki MatsushitaYeu SuKe ChenYeul Hong KimKe-Cheng ChenYeun Kyu JangKee Siang LimYevgeniy R. SemenovKeechilat PavithranYevgeniya IoffeKe-Hung TsuiYi HuKei EndoYi Hui WuKei ShibuyaYi LuanKeiji HanadaYi QiuKeiko IwaisakoYi ShanKeita KaiYi SunKeith LauYi XuKekoa TaparraYi ZhaoKelly Lisa Bowlt BlacklockYichen LiKelly P. A. ByrneYicheng MaoKelly Yi Ping LiuYifan ZhouKelsey L. PomykalaYifei LiaoKemal SümserYigang YangKemmyo SugiyamaYihenew Simegniew BirhanKen ItoYih-Shou HsiehKen KatoYi-Hsien HsiehKen KuduraYi-Hsuan HsiaoKen MillsYi-Hung LiaoKendrick KooYi-Jang LeeKenei FurukawaYi-Jen LiaoKengo SasakiYiji LiaoKenichi SudaYi-Jou TaiKen-ichi YoshiokaYimeng KongKenji FujiwaraYiming ZhangKenji HayashidaYin SunKenji NakanoYing Bin YanKenji TagoYing Chih ChangKenk MiranYing HuangKenneth P. NephewYing LiuKenneth S. HettieYing QingKenneth SimbiriYingbo LinKennosuke KarubeYing-Cheng ChiangKenny ChitcholtanYing-Chin KoKenry KenryYingchun HouKenshi SuzukiYin-Hwa ShihKenshiro ShiraishiYinling HuKentaro ItoYinting ChenKentaro YoshiokaYi-Ren HongKenya KamimuraYirong ChenKenza E. BenzeroualYi-Ting WangKepeng WangYixiang LiaoKermit L. CarrawayYixuan GuoKersti OselinYizhi SongKerstin BorgmannYoav D. ShaulKesava ReddyYoelsis García-MayeaKeshav Raj PaudelYohei KitamuraKęstutis RimaitisYohei MiyagiKeun Soo AhnYohei SekinoKevin D. BoydYoichi MatsuoKevin HarringtonYoichi ShimizuKevin M. SullivanYoji KishiKevin MartellYoji NagashimaKevin StephansYok-Lam KwongKevin Tak Pan NgYoko TarumiKevin X. LiuYona KeisariKeyla Vargas-RománYong Beom KimKeyoumars AshkanYong Jung SongKhac-Minh ThaiYong LiKhaled ElsayadYong TengKhalid I. BzeiziYong Weon YiKhalil AhmedYong-Chan KimKhara SauroYong-Chen ChenKheira HirecheYongde LuoKhoja LeilaYongfeng ZhaoKhosrow RezvaniYongji ZengKhushminder RaiYongjun SuiKi Hyun NamYongkang YangKian KaniYong-Moon LeeKiattawee ChoowongkomonYongping BaoKibeom KimYongqiang ChenKi-cheong ParkYong-Wha MoonKim BlenmanYong-Xin LiuKim Tae HyunYongzhang LuoKimberly HoangYongzhen LiuKimberly PerezYonis GulzarKin Yin CheungYoon-young ChoiKinan Drak AlsibaiYoo-Seok YoonKiran Kumar MangalaparthiYosef ShamayKiran SandhuYosef ShaulKirill RosenYoshi OdakaKirsi KetolaYoshiaki YuraKirsten LindnerYoshihiko YanoKishor PantYoshihiro KomoharaKishu RanjanYoshihiro NoguchiKishwor PoudelYoshihiro OnoKi-Tae HaYoshihiro ShidojiKitty PavlakisYoshikane YamauchiKiyomi HoriuchiYoshikazu NakamuraKiyong NaYoshinobu KariyaKiyoshi MaedaYoshinori InagakiKiyoshi MasudaYoshiro ItataniKiyoshi TakagiYoshishiro HiramatsuKlaas KokYoshitaro ShindoKlaudia BanachYoshito NishimuraKlaudia Izabela KocurekYoshiyuki NakamuraKlaudija ViškovićYoshiyuki ShioyamaKlaus EmmanuelYoshiyuki SueharaKlaus FortscheggerYosuke MatsuuraKlaus WeberYosuke MiuraKlaus-Martin SchulteYosuke TakakusagiKo OkumuraYosuke ToyodaKoen J. HarteminkYou WuKohei OkuyamaYoug Raj ThakerKoji KomoriYoung ChoiKoji MitaYoung Eun YoonKoji MiyanishiYoung KimKoji OtaniYoung Shin SongKoji SawadaYoung Wha KohKojiro TauraYoung-Chang AraiKok Siong ChenYoung-Hye SongKoldo Garcia-EtxebarriaYoung-Youn ChoKolja ThierfelderYoun-Jin ChoiKomal AroraYouping DengKomuraiah MyakalaYousuke NakaiKonda Mani SaravananYousuke TsujiKondapa Naidu BobbaYu Jin LimKondareddy CherukulaYu Kyung ChoKonosuke NakajiYu ShenKonrad A. SzychowskiYu TakahashiKonrad HuppiYu ZhangKonstantin OkonechnikovYu ZhuKonstantin PanovYuan LiKonstantina PanoutsopoulouYuan LiuKonstantinos ChatzipapasYuan-Chi LinKonstantinos ChronisYuan-Hung WangKonstantinos EvangelouYuanhung WuKonstantinos LasithiotakisYuanjie NiuKonstantinos SapalidisYuanyan XiongKonstantinos StamatakisYubin GeKonstantinos TosiosYu-Chan ChangKonstantinos VlachosYu-Chen S. H. YangKoos HovingaYu-Chieh ChenKoraljka Gall TrošeljYu-Chieh SuKoraljka Rajković-MolekYu-Chih ChenKorinna JöhrensYu-Ching ChouKosei MatsueYu-Ching LinKoshi AkahaneYu-De ChuKosho YamanouchiYudong CaiKosuke MiyaiYu'e LiuKosuke YoshidaYue ShengKoteswara Yerra RaoYue WangKoteswararao GarikapatiYueh ChienKouji IzumiYueh Z. LeeKourosh HooshmandYuemeng JiaKousalya PrabaharYu-Fang HuangKoushiro OhtsuboYufei ShiKousik KeshYuh BabaKousik SundararajanYuhan HaoKowichi JimbowYuhei GotoKrešimir RotimYuhei MatsudaKrinio GiannikouYuhei MiyasakaKrishna Mohan ParsiYuh-Seog JungKrishnendu PalYu-Hung ChenKristen Donelle BrantleyYuichi MushimotoKristiaan J. LenosYuichi SaitoKristian T. SchafernakYuichiro HatanoKristijan SkokYuji MurakamiKristin Katharina RallYuji NishikawaKristin LangYuji ShimizuKristina AndrijauskaiteYujiro HayashiKrisztina KőhalmyYujiro NishiokaKriti MittalYuki HagaKruthi SuvarnaYuki KatoKrutika PatelYuki NakamuraKrzysztof FornalskiYuki SaitoKrzysztof GorynskiYuki ShinyaKrzysztof KaliszewskiYuki YamashitaKrzysztof LetachowiczYukie YoshiiKrzysztof SztankeYukihide WatanabeKrzysztof TomasiewiczYukiko NakaharaKrzysztof TupikowskiYukinori OzakiKrzysztof ZieniewiczYukio NayaKseniya Petrova-DrusYukio SekiKu Youn BaikYuko TashimaKuan Yu HsiehYuliang WuKuan-Fu LiaoYu-Ling LinKuang-Hung ChengYuma FuseKuang-Tai KuoYu-Min ShenKuei-Yang HsiaoYun ShiKuen-Cheh YangYun-Chung CheungKui ZhangYunfei YuanKuikun YangYunfeng ZhaoKuldeep GuptaYung-Kuan TsouKu-Lung HsuYung-Luen YuKumar S. BishnupuriYung-Shin SunKumiko KarasawaYung-Shuo KaoKun HeYung-Yu HsiehKun SongYunjin BaiKun YangYun-Kee JoKunal AjmeraYunpeng YeKundan MishraYuntao BingKun-Eek KilYun-Wen ZhengKunihiro TsukasakiYunxiang DaiKunka Mohanram RamkumarYunxiang MuKunwei ShenYu-Peng LiuKun-Yun YehYu-Peng ZhouKuo-Ching MeiYuqi ChenKurian JosephYuqing ZhangKushal KandhariYuri DubrovaKwaku Dad Abu-BonsrahYuriy AlekseyevKwang-sig LeeYuriy L. OrlovKwok-Wai LoYurong SongKwon-Ho HongYury GlazyrinKyeong-Man HongYury KistenevKyle B. WilliamsYu-Sheng ShenKyle VanKoeveringYushi SuzukiKylynda C. BauerYusuf TutarKyoung Sik ParkYusuke KawamuraKyu Young ChoiYusuke KuritaKyuBum KwackYusuke OmeKyung Sik ParkYusuke SatoLabib G. DebianeYusuke SuenagaLadislav BatalikYusuke TomitaLaetitia GuevelYusuke YamamotoLaetitia VercellinoYuta EndoLaffranchi MattiaYuta ShibamotoLafon-Hughes LauraYutaka KishidaLaia DomingoYutaka MatsudaLaila ZikoYutaka MidorikawaLaith AlzubaidYutaka NaitoLajos GergelyYutaka YasuiLakmini SenavirathnaYuting YuanLakshminarayan Reddy TeegalaYuto YasudaLalu IrhamYuuya KasaharaLamprou MargaritaYu-Wei LiuLan ZhouYuyang JiangLara DavisYu-Yun ShaoLara K. AbramowitzYves DelnesteLara R. HeijYves HenryLarisa IvascuYves JacquotLarisa LitovchickYves St-PierreLarisa RyskalinYvette AnderssonLars BöckmannYvonne LiLars C. HankerYvonne M. H. Versleijen-JonkersLars Henrik JensenYvonne SchrageLars Joachim LindbergZabit HameedLars MichelZachary F. BurtonLars NyZachary HunterLars SchwettmannZachary SmithLashan PeirisZahi I. MitriLászló MangelZahid UllahLászló ŐrfiZahra GhanianLászló PávicsZahra RattrayLászló SzilákZaid SiddiquiLaszlo TretterZaili LuoLatifa BakiriZakaria Y. Abd ElmageedLatifa Rbah-VidalZaki A. SherifLaukhtina EkaterinaZakia AkterLaura BerettaZbigniew AdamczewskiLaura BergantiniZbigniew J. WitczakLaura BukavinaZdenek KejikLaura C. Hernández-RamírezZecchin DavideLaura ColeZecchini SilviaLaura EadieZeenat FarooqLaura EvangelistaZefang RenLaura Francesca PisaniZekiye AltunLaura GianottiŽeljko AntićLaura GiordanoZeng-Quan YangLaura HüserZenichi MoriseLaura J. KlesseZenon PidsudkoLaura KasmanZenon PogorelićLaura La PagliaZhang LiLaura ManciniZhao ZhangLaura MarchettiZhaohui FengLaura MartinZhaohui XiongLaura MaziluZhe JinLaura Mora-LópezZhe YangLaura MoroZhen LuLaura PatrussiZhen ShuLaura PopZheng ChenLaura RidolfiZheng FuLaura S. Hiemcke-JiwaZheng XiaLaura S. SchmidtZheng ZengLaura SalvadoriZheng ZhongLaura SanzZhenghong LeeLaura Sterian WardZhengnian LiLaura TackZheng-Xin WangLaura V. KlotzZheng-Yuan SuLaura VizkeletiZhenwen ZhaoLaura-Nanna LohkampZhenzhen ZhangLauréline RogerZheqi LiLauren GollahonZhi ShiLauren HurwitzZhibo MaLaurence BianchiniZhichuan ZhuLaurence Bresson-BepoldinZhifan GaoLaurence ChampionZhihai QinLaurence LafanechereZhijian HeLaurence LagneauxZhijie ChangLaurens A. Van MeeterenZhijun ZhouLaurent CronierZhila Semnani-AzadLaurent GuyonZhi-Min YuanLaurent MachetZhipeng TaoLaurent QueroZhiping FengLaurent VilleneuveZhiqiang ZhangLaurenția Nicoleta GaleșZhishan ChenLaurentiu M. PopZhiwei WenLaurentiu PirteaZhiyong WangLaurie M. ElitZhiyu Amy YangLawrence SowersZhiyuan XuLaychiluh MekonnenZhiyun GuoLayth Mula-HussainZhonghua SunLazaros TzelvesZhongqiu XieLe Nguyen Quoc KhanhZhongwei LiuLe Xuan Truong NguyenZhouwei ZhangLe YingZhubo WeiLéa DoussetZhuqing LiLea M. MondayZiad ZaatariLea ScherschinskiZijun Yidan Xu-MonetteLeandro M. O. LourençoZili GuLeandro SantiagoZiliang HuangLeandro SastreZilvinas DambrauskasLechuang ChenŽilvinas SaladžinskasLeendert H. J. LooijengaZiqing ChenLee-Yung ShihZiv RadisavljevicLefort SylvainZivar YousefipourLei ChangZixin ChenLei DuanZiyue LiLei GaoZlatin ZlatevLei HuangZlatko DembicLei JiangZoe WaltersLei ShiZofia MazerskaLei WanZohaib KhurshidLei ZhangZohar DotanLeif C. AnderssonZohar LeviLeire PedrosaZohreh AmoozgarLekh DahalZoltan AntalLena ConradiZoltán BalajthyLena HaeberleZoltan HeroldLena HieggelkeZoltán PethőLenka StixovaZoltán WienerLeny JoseZonghua LuoLeo MassariZsófia BognárLeonard Christopher SchmeelZsolt TothLeonardo CentonzeZsuzsa SchaffLeonardo FranzZsuzsanna Bagó-HorváthLeonardo MessiasZsuzsanna BirkóLeonardo RestaZsuzsanna KahánLeonardo Saúl Lino-SilvaZulfiqar HabibLeonardo TariciottiZulqurnain SabirLeonardo V. M. de AssisZuoren YuLeonhard GruberZuoxu XieLeonidas N. DiamantopoulosZu-Quan HuLeonie KonczallaZuzana Macek JilkovaLéonor BenhaïmZuzanna Urban-WojciukLeopoldo CostarelliZvia AgurLesley Smith


